# A novel lncRNA linc-AhRA negatively regulates innate antiviral response in murine microglia upon neurotropic herpesvirus infection

**DOI:** 10.7150/thno.64880

**Published:** 2021-09-21

**Authors:** Yiliang Wang, Weisheng Luo, Lianzhou Huang, Ji Xiao, Xiaowei Song, Feng Li, Yuying Ma, Xiaohui Wang, Fujun Jin, Ping Liu, Yexuan Zhu, Kaio Kitazato, Yifei Wang, Zhe Ren

**Affiliations:** 1 Guangzhou Jinan Biomedicine Research and Development Center, National Engineering Research Center of Genetic Medicine, Institute of Biomedicine, College of Life Science and Technology, Jinan University, Guangzhou, China.; 2 Key Laboratory of Virology of Guangdong province, Jinan University, Guangzhou, China.; 3 Guangdong Province Key Laboratory of Bioengineering Medicine, Jinan University, Guangzhou, China.; 4 Guangdong Provincial biotechnology drug & Engineering Technology Research Center, Jinan University, Guangzhou, China.; 5 Department of Clinical Research Pharmacy, Graduate School of Biomedical Sciences, Nagasaki University, 1-14 Bunkyo-machi, Nagasaki 852-8521, Japan.

**Keywords:** Microglia, neurotropic virus, long non-coding RNA, conserved fragment, TBK1, TRIM27, aryl hydrocarbon receptor (AhR)

## Abstract

Microglia are the primary cellular source of type I interferons (I-IFNs) in the brain upon neurotropic virus infection. Although the I-IFN-based antiviral innate immune response is crucial for eliminating viruses, overproduction led to immune disorders. Therefore, the relatively long-lasting I-IFNs must be precisely controlled, but the regulatory mechanism for the innate antiviral response in microglia remains largely unknown. Long non-coding RNAs (lncRNAs) are being recognized as crucial factors in numerous diseases, but their regulatory roles in the innate antiviral response in microglia are undefined.

**Methods:** The high-throughput RNA sequencing was performed to obtain differentially expressed lncRNAs (DELs) in primary microglia infected with or without the neurotropic herpes simplex virus type 1 (HSV-1). We selected four DELs ranked in the top 15 in basic level and their fold change induced by HSV-1, i.e., FPKM_HSV-1_/FPKM_Cells_.We subsequently found a key lncRNA affecting the innate antiviral response of microglia significantly. We next used dual-luciferase reporter assays, bioinformatical tools, and truncation mutants of both lncRNA and targeted proteins to elucidate the downstream and upstream mechanism of action of lncRNA. Further, we established microglia-specific knock-in (KI) mice to investigate the role of lncRNA* in vivo*.

**Results:** We identified a long intergenic non-coding RNA, linc-AhRA, involved in regulating the innate antiviral response in murine microglia. *linc-AhRA* is activated by aryl hydrocarbon receptor (AhR) and restricts I-IFN production in microglia upon neurotropic herpesvirus infection and innate immune stimulation. Mechanistically, linc-AhRA binds to both tripartite motif-containing 27 (TRIM27) and TANK-binding kinase 1 (TBK1) through its conserved 117nt fragment as a molecular scaffold to enhance TRIM27-TBK1 interaction. This interaction facilitates the TRIM27-mediated ubiquitination of TBK1 and results in ubiquitin-proteasome-dependent degradation of TBK1. Consequently, linc-AhRA suppresses I-IFN production through facilitating TBK1 degradation and limits the microglial innate immune response against neurotropic herpesvirus infection. Microglia-specific KI of linc-AhRA mice shows a weakened antiviral immune response upon neurotropic herpesvirus challenge due to a reduction of TBK1 in microglia.

**Conclusion:** Our findings indicate that linc-AhRA is a negative regulator of I-IFN production in microglia to avoid excessive autoimmune responses. These findings uncover a previously unappreciated role for lncRNA conserved fragments in the innate antiviral response, providing a strong foundation for developing nucleotide drugs based on conserved functional fragments within lncRNAs.

## Introduction

Virus-derived components can be recognized by pattern-recognition receptors (PRRs) within host cells, triggering the expression of type I interferons (I-IFNs) (IFN-α/β) via activation of the interferon regulatory factor 3 (IRF3) and nuclear factor-κB (NF-κB) signalling 1, 2. I-IFNs act as autocrine and paracrine activators of I-IFN receptors to induce the expressions of numerous interferon-stimulated genes (ISGs) encoding a subset of antiviral proteins 3. Microglia are the brain's main cellular source of I-IFNs upon neurotropic virus infection 2, 4-6. I-IFNs from microglia also orchestrate the antiviral defence to other cells in the CNS 1, 2, 7. However, the excessive expression of I-IFNs has an overall detrimental effect on the CNS 1, 4, 6, 8. Therefore, a systematic but flexible regulatory mechanism is required for microglia to balance I-IFN production and efficiently eliminate invading viruses while avoiding immunopathology. TANK-binding kinase 1 (TBK1) is a crucial factor that mediates the activation of IRF3, leading to the induction of IFN-α/β following viral infections 9. TBK1 activity is tightly regulated by a variety of post-translational modifications (PTMs), such as phosphorylation, ubiquitination (Ubi), and the prevention of functional TBK1-containing complex formation 10-12. The E3 ubiquitin ligase TRIM27 interacts with TBK1 and leads to Ubi-mediated degradation of TBK1, resulting in the suppression of IRF3 activation and IFN-α/β production 10, 13. However, the intrinsic inhibition pathway for the IFN response may be leveraged by viruses. Therefore, a balance between the host innate antiviral response and virus immune evasion is pivotal to virus pathogenesis. Identification of the key molecules involved in microglia-neurotropic virus interactions contributes to a better understanding of the innate immune homeostasis in the CNS. Previous studies focused on the regulatory network in non-CNS macrophages but provided a limited understanding of the regulatory mechanism for the innate antiviral response in CNS. Herpes simplex virus type 1 (HSV-1) is the most common human neurotropic viruses, infection of which causes numerous diseases, including herpes simplex encephalitis (HSE) that has a high mortality rate if left untreated, and herpetic stromal keratitis (HSK) is the leading cause of infectious blindness 14, 15. Moreover, the close association between HSV-1 and Alzheimer's disease has been gradually recognized 16, although the underlying mechanism remains obscure. A comprehensive investigation of the microglial immune response against HSV-1 infection would be beneficial for understanding the brain pathogenesis induced by HSV-1 infection.

Long non-coding RNAs (lncRNAs) are gradually recognized as significant components of the innate antiviral response 17-19, but functional lncRNAs in the microglial innate antiviral response remain unknown. Indeed, due to the lack of a translation process, non-coding transcripts would be more efficient for regulating the response to stress such as virus infection, which requires a rapid turn-over. In particular, lncRNAs participate in the regulation of numerous PTMs 19. Moreover, although most lncRNAs are not conserved, a few lncRNAs have been found to harbour conserved fragments 20-22. Besides, we cannot ignore the side effects generated by full-length lncRNAs *in vivo*. We report a novel AhR-activated long intergenic non-coding RNA, linc-AhRA, which negatively regulates the microglial innate antiviral response. linc-AhRA serves as a molecular scaffold to enhance TRIM27-TBK1 interactions via its conserved 117nt fragment at the late phase of the antiviral response. This interaction facilitates TRIM27-mediated ubiquitination of TBK1 and leads to TBK1 degradation. As a result, linc-AhRA suppresses I-IFNs production and limits the microglial innate immune response against neurotropic HSV-1 infection, which HSV-1 exploits for immune escape. This study provides new insights into the regulation mechanism in the microglial I-IFN response against neurotropic herpesvirus infection.

## Results

### linc-AhRA is associated with neurotropic herpesvirus infection and innate antiviral response in microglia

**Figure 1:**
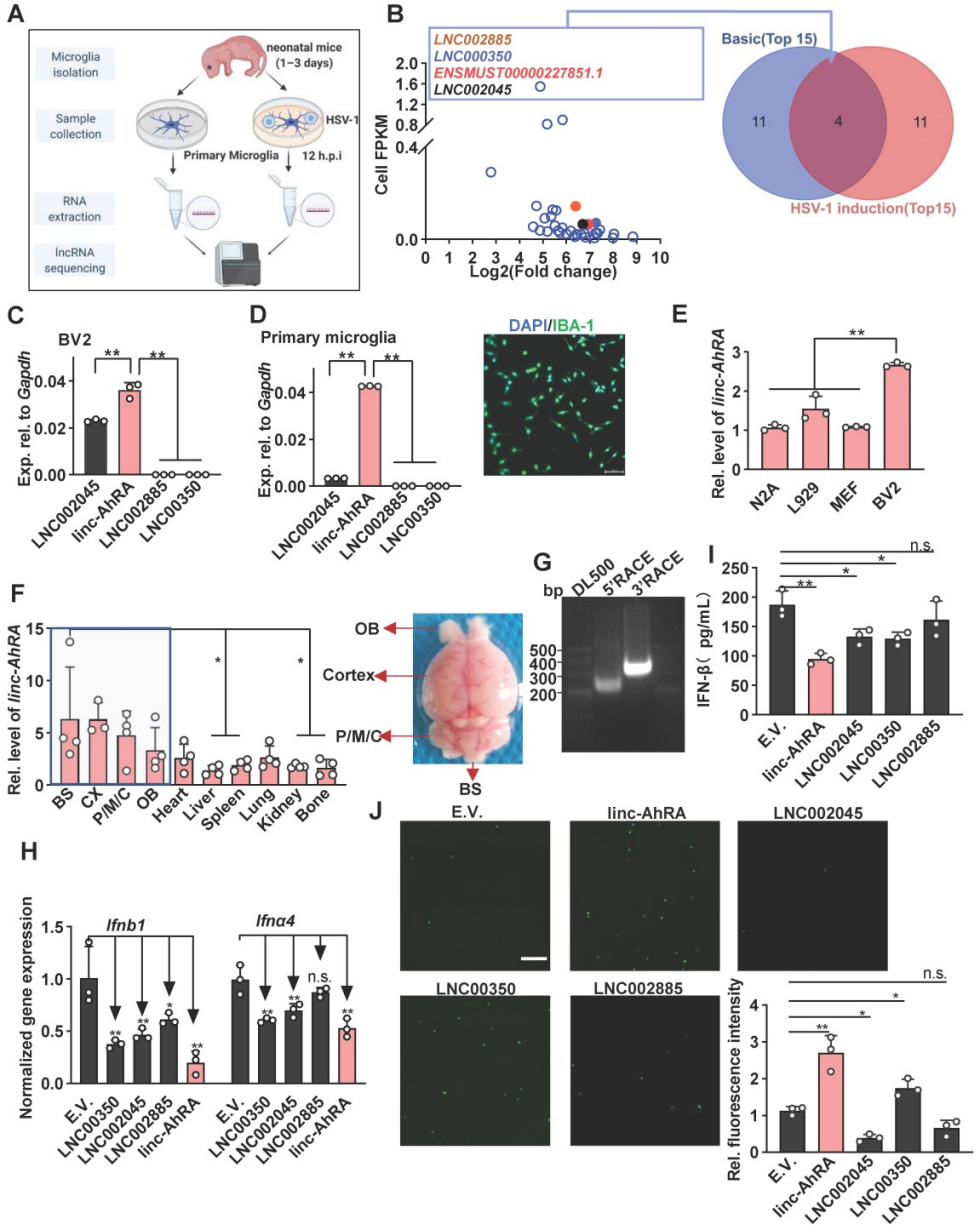
** linc-AhRA was identified as a lncRNA involved in the innate antiviral response in microglia upon neurotropic herpesvirus infection. A,** Schematic illustration of lncRNA-sequencing workflow for total RNA samples from primary microglia with or without HSV-1 infection (multiplicity of infection (MOI) 1).** B,** Left: Scatter plot for all upregulated DELs presenting their FPKM values in cells and the fold change induced by HSV-1 (novel lncRNA with the prefix LNC and annotated lncRNA with the prefix ENSMUST). Right: Venn diagram analysis of the overlapped DELs ranking in the top 15 in both basic abundance and fold change upon HSV-1 infection. **C,** qPCR analysis of the expression of the indicated lncRNAs in BV2 cells. **D,** Left: qPCR analysis of the expression of the indicated lncRNAs in primary microglia. Right: Fluorescence microscopy images of primary microglia to determine the purification of microglia by staining IBA-1 (green). Scale bar, 100 μm. **E,** qPCR analysis of *linc-AhRA* expression in the indicated cell lines. **F,** qPCR analysis of linc-AhRA expression in mice tissues (normalized relative to the liver) (left). Only the statically comparison result between BS and other non-CNS tissues was labelled. A representative mouse brain with a label of respective region was also provided (right). **G,** 5'RACE and 3'RACE results for RNA from primary microglia infected with HSV-1 for 12 h to obtain the 5' and 3' end sequences of linc-AhRA. The normalized gene expressions were present. **H,** qPCR analysis of* Ifnα4* and *Ifnb1* mRNA levels in BV2 cells transfected with plasmids expressing the indicated lncRNAs for 48 h followed by HSV-1 infection (MOI 1) for 6 h (empty vector, E.V.). **I,** ELISA of IFN-β in the supernatants of BV2 cells transfected with plasmids expressing the indicated lncRNAs for 48 h followed by HSV-1 infection (MOI 1) for 12 h. **J,** Left: Fluorescence microscopy images of viral replication (green) in BV2 cells infected with EGFP-HSV-1 (MOI 1) for 24 h, following a transfection of plasmids expressing the indicated lncRNAs for 48 h. Scale bar, 500 μm. Right: The quantitative result for relative EGFP fluorescence intensity obtained from three independent experiments. Data are representative of three independent experiments (G, J), three independent experiments with n = 3 technical replicates (C-E, H-I), the average of three technical replicates as a mixture (B), each symbol represents an individual technical replicate or a mouse (C-F, H-I) or one independent experiment (J) (shown as mean and s.d. in C-F, H-I), two-tailed unpaired Student's t-test (C-E, H-J).

We first performed the dynamic analysis of IFN mRNA expression in microglia upon neurotropic herpes simplex virus type 1 (HSV-1) infection at different hours post-infection (h.p.i). We found that I-IFNs appeared to decrease in the late stage of HSV-1 infection (**Figure S1A**). To investigate whether lncRNAs were involved in this process, we performed high-throughput RNA sequencing of primary microglia infected with or without HSV-1 and obtained differentially expressed lncRNAs (DELs) (**Figure 1A** and **Figure S1B**). The upregulated DELs were ranked according to their fragments per kilo-base of exons per million fragments (FPKM) in the steady-state and their fold change induced by HSV-1, i.e., FPKM_HSV-1_/FPKM_Cells_ (**Table S1** and **Figure 1B**). Only four upregulated DELs ranked in the top 15 in both contexts, namely LNC002885, LNC000350, ENSMUST00000227851.1 (linc-AhRA), and LNC002045 (**Figure 1B**). ENSMUST00000227851.1 is an aryl hydrocarbon receptor (AhR) activated-long intergenic non-coding RNA (lincRNA) and hereafter referred to it as linc-AhRA. linc-AhRA attracted our attention for the following reasons: (1) Among those four lncRNAs, linc-AhRA is the most abundant lncRNA in microglia-like BV2 cells (**Figure 1C**) and primary microglia (**Figure 1D** and **Figure S1C**); (2) BV2 cells have a higher abundance of linc-AhRA than neuron-like Neuro-2a, MEF, and L929 cells (**Figure 1E**); (3) An enrichment of linc-AhRA was observed in CNS tissues, including in the brain stem (BS), cerebral cortex, pons, medulla, and cerebellum (P/M/C), and olfactory bulb (OB) (**Figure 1F**). We performed 5' and 3' rapid amplification of complementary DNA ends (RACE) assays to determine the full length of linc-AhRA in primary microglia (**Figure 1G**). We found that linc-AhRA contains 682nt without a poly-adenylated (poly-A) tail (**Table S2**). Both the coverage tracks from the RNA-seq and Ensemble annotation indicated that the gene locus of linc-AhRA was located on chromosome 15, nucleotides 25, 414, 192-25, 414, 873 that linc-AhRA did not overlap with known protein-coding genes (**Figure S1D-E**). Our subsequent screen assay indicated that linc-AhRA significantly decreased the mRNA level for the I-IFNs, *Ifnb1* and *Ifna4*, and the protein level for IFN-β, in response to HSV-1 infection (**Figure 1H-I**) and facilitated EGFP-HSV-1 infection in microglia-like BV2 cells (**Figure 1J**). Overexpression of linc-AhRA also led to an upregulation of virus titer (**Figure S1F**).

Next, we tested whether linc-AhRA acts as non-coding RNA in the innate antiviral response. Bioinformatics analysis using the Coding Potential Calculator showed that linc-AhRA lacks coding potential 23 (**Figure S2A**), with a PhyloCSF score < 0 according to UCSC Genome Browser analysis (**Figure S2B**). However, the NCBI Open Reading Frame (ORF) Finder showed three ORFs with more than 70 amino acids in linc-AhRA (**Figure S2C**)**.** To investigate whether these ORFs of linc-AhRA can stably translate to peptides, we generated several plasmids encoding the ORFs fused with an enhanced green fluorescent protein (EGFP) at their N terminus. The immunoblotting results indicated that EGFP-fused ORF1 and ORF4 can generate EGFP-tagged peptides in HEK 293T cells, but not EGFP-fused ORF3 or the full-length linc-AhRA (**Figure S2D**). However, neither of these ORF-coding small peptides reduced the expression of *Ifnb1* and *Ifna4* upon HSV-1 infection (**Figure S2E**)**.** Consistently, only the full length of linc-AhRA, but not these ORF-coding small peptides, can result in an increment of HSV-1 titer (**Figure S2F**). Indeed, linc-AhRA was cloned into the plasmids using frameshift mutation, given its non-coding ability. These results indicated that the function of linc-AhRA in the innate antiviral response in microglia is not due to its encoding small peptides.

**Figure 2:**
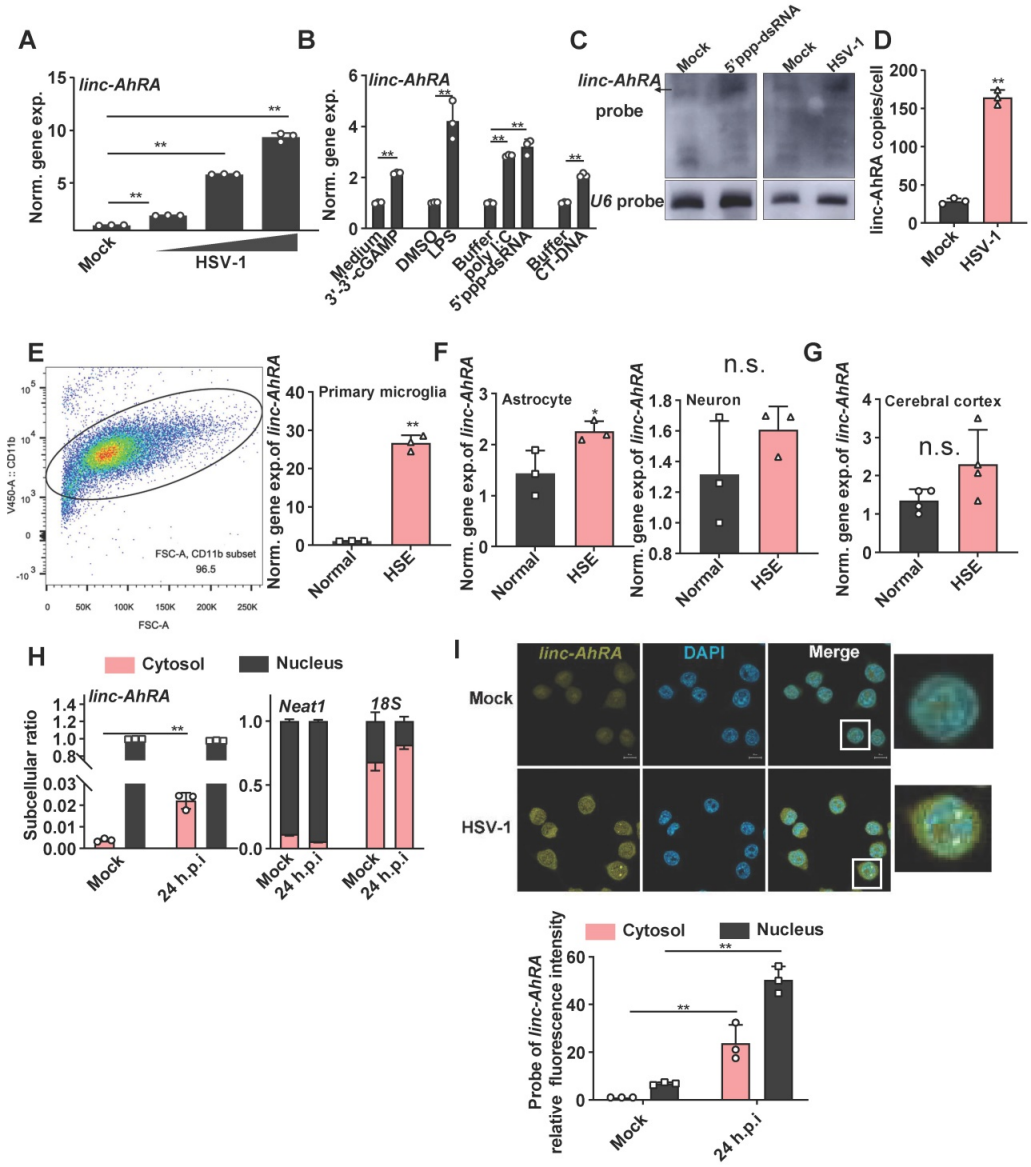
** linc-AhRA is upregulated in response to numerous innate stimuli and relocated into the cytosol upon HSV-1 infection. A,** qPCR analysis of linc-AhRA level following a 12 h infection with HSV-1 in BV2 cells with MOI as labeled. The “normalized gene expression” was abbreviated into “norm. gene exp.”. **B,** qPCR analysis of linc-AhRA level in BV2 cells with the indicated stimulation for 6 h (3'-3'-cGAMP, 5'ppp-dsRNA, and CT-DNA, 1 μg/ml; LPS, 100 ng/ml).** C,** Northern blotting analysis for RNA from BV2 cells with or without HSV-1 (MOI 1) or 5'ppp-dsRNA stimulation (1 μg/ml) for 12 h. **D,** Copy-number analysis of linc-AhRA in BV2 cells infected with HSV-1 (MOI 1) for 9 h using qPCR. **E, Left**: Flow cytometry analysis of microglia following acute isolation from mice using CD11b-coupled magnetic beads. The ratio of the CD11b^+^ subset is also presented. **Right**: qPCR analysis of linc-AhRA level in microglia acutely isolated from normal and HSE mice at 9 d.p.i. **F,** qPCR analysis of linc-AhRA level in primary astrocyte (**left**) and neuron (**right**) isolated from normal and HSE mice at 9 d.p.i. **G,** qPCR analysis of linc-AhRA level in cerebral cortex from normal and HSE mice at 9 d.p.i.** H,** qPCR analysis of cytoplasmic and nuclear ratios of *linc-AhRA*, *Neat1*, and *18s* in BV2 cells after HSV-1 infection for 24 h. **I, Top**: RNA FISH showing *linc-AhRA* (yellow) in BV2 with HSV-1 (MOI 1) infection for 24 h. The nuclei were stained with DAPI (cyan). Scale bar, 10 μm. **Bottom**: The relative fluorescence intensity of *linc-AhRA* in the cytoplasm and nucleus from three random regions was determined using the ImageJ program. Data are representative of three independent experiments (**C, I[top]**), three independent experiments with n =3 technical replicates** (A-B, D-E[right], F, H-I)** or with n = 4 individuals (**G**), each symbol represents an individual or technical replicate or one region **(A-B, D-E[right], F-I[bottom])** (shown as mean and s.d. in **A-B, D-E[right], F-I[bottom]**), two-tailed unpaired Student's t-test (**B, D-E[right], F-I[bottom]**), One-way ANOVA (and nonparametric analysis) (**A**).

We next explored the association between linc-AhRA and virus infection in microglia. linc-AhRA exhibited a virus dose-dependent upregulation in response to HSV-1 (**Figure 2A**). linc-AhRA could also be induced by numerous innate stimuli, including cyclic guanosine monophosphate-adenosine monophosphate (cGAMP, a stimulator of interferon gene (STING) ligand), toll-like receptor 4 (TLR4) ligand lipopolysaccharide (LPS), TLR3 ligand poly (I: C), 5'ppp-dsRNA, and calf thymus DNA (CT-DNA) (**Figure 2B**). Consistently, RNA blotting assays indicated that linc-AhRA was remarkably upregulated in BV2 cells in response to 5'ppp-dsRNA stimulation and HSV-1 infection (**Figure 2C**). Absolute copy number analysis revealed that linc-AhRA was expressed at relatively low levels with ~25 transcript copies per microglia, which increased to ~174 copies per cell upon HSV-1 infection (**Figure 2D**), with an abundance similar to that of other functional lncRNAs 24. We next established the herpes simplex encephalitis (HSE) mice model. The microglia acutely isolated from the HSE mice had a higher abundance of linc-AhRA than those from normal mice (microglia purification: 96.5%) (**Figure 2E**). However, linc-AhRA did not upregulate significantly in astrocytes and neurons isolated from HSE mice (**Figure 2F**). The level of linc-AhRA in the cerebral cortex tissue from HSE mice was upregulated minorly compared to those from normal mice (**Figure 2G**). Interestingly, the qRT-PCR results for the subcellular fraction of RNAs indicated that linc-AhRA was mainly localized in the nucleus in resting microglia and that HSV-1 infection led to an increment of linc-AhRA in the cytoplasm (**Figure 2H**). Similarly, the RNA fluorescence *in situ* hybridization (FISH) assay demonstrated that HSV-1 infection increased linc-AhRA puncta and enhanced the nuclear export of linc-AhRA at the late phase of infection (**Figure 2I**). Together, linc-AhRA was significantly upregulated and translocated to the cytoplasm from the nucleus of the microglia in the late stage of neurotropic herpesvirus infection.

### linc-AhRA is mainly regulated by AhR, which is activated by HSV-1 infection

**Figure 3:**
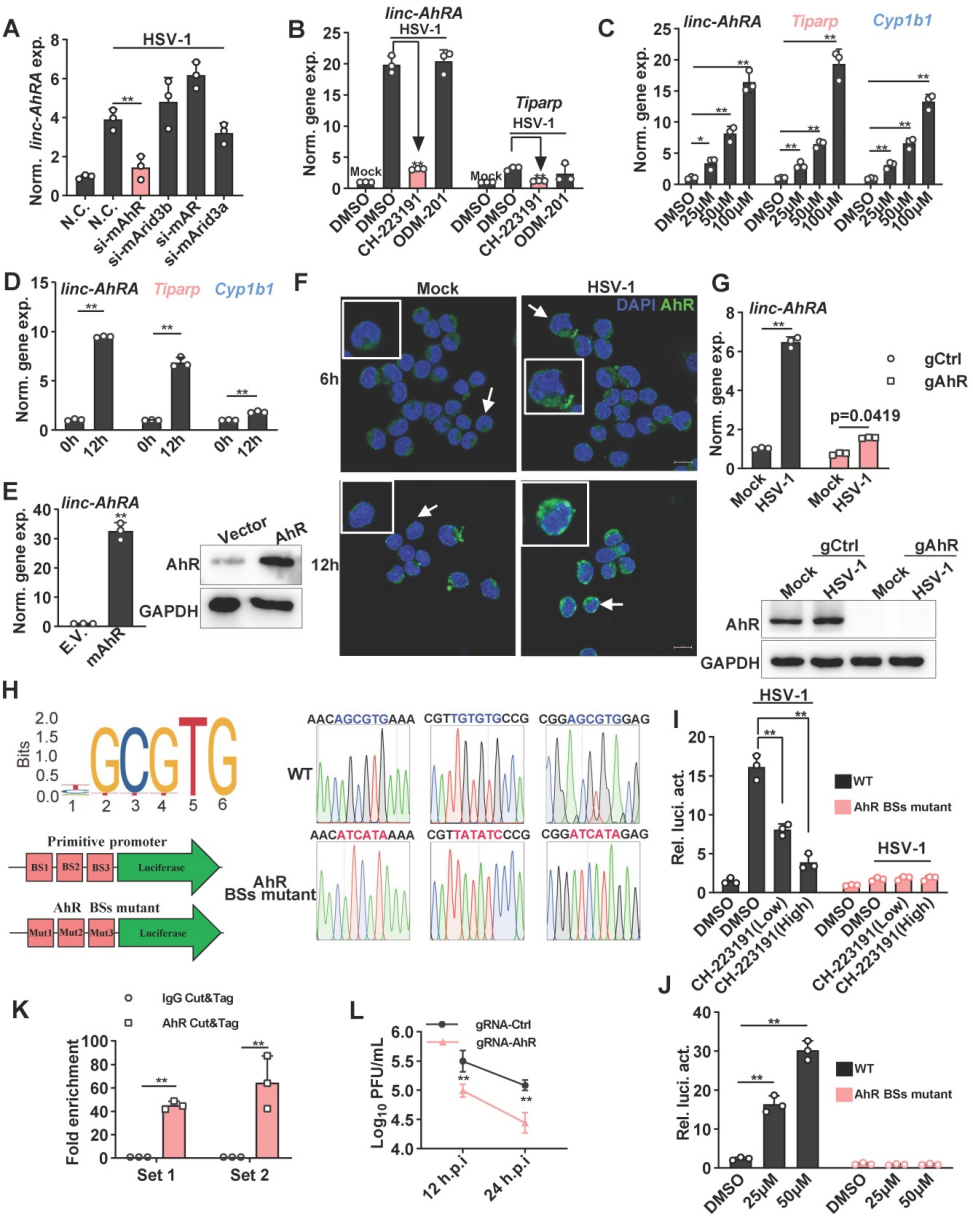
** The induction of linc-AhRA by HSV-1 infection is mainly mediated by AhR signaling. A,** qPCR analysis of *linc-AhRA* expression in BV2 cells 48 h after transfecting the indicated siRNA and HSV-1 infection (MOI 1) for 9 h.** B,** qPCR analysis of the expression of the indicated genes in BV2 cells infected with HSV-1 (MOI 1) for 12 h in the presence of CH-223191 (50 nM) and ODM-201 (100 nM).** C,** qPCR analysis of the expression of the indicated genes in BV2 cells 9 h after stimulation with indirubin with the indicated concentration. **D,** qPCR analysis of the expression of the indicated genes in BV2 cells with HSV-1 infection (MOI 1) for 12 h.** E, Left**: qPCR analysis of *linc-AhRA* expression in BV2 cells 48 h after the transfection of AhR-expressing plasmids; **right**: Immunoblot analysis of AhR level in BV2 cells 48 h after the transfection of AhR-expressing plasmids.** F,** Confocal microscope-based fluorescence analysis of the nuclear localization of AhR (green) in BV2 cells with HSV-1 infection (MOI 1) for the indicated hours. Nuclei were labeled with DAPI (blue). Scale bars, 10 μm.** G, Top**: qPCR analysis of *linc-AhRA* expression in gRNA-based AhR-deficient or control BV2 cells with HSV-1 infection (MOI 1) for 9 h; **bottom**: Immunoblot analysis of AhR level in gRNA-based AhR-deficient or control BV2 cells with HSV-1 infection (MOI 1) for 9 h. **H, Left (top):** The sequence logo recognized and bound by AhR; **left (bottom)**: Schematic illustration for the construction of a firefly luciferase reporter driven by the primitive promoter or AhR binding site (BS) mutant promoter of linc-AhRA; **right**: The Sanger sequencing peak for the AhR-binding site mutant and primitive promoter-driven luciferase plasmids. **I,** Dual luciferase analysis of linc-AhRA primitive and AhR-binding site mutant promoter activity in BV2 cells 24 h after co-transfection of a firefly luciferase reporter (linc-AhRA pro.-luc) and TK-renilla, followed by HSV-1 infection (MOI 1) for another 12 h.** J,** Dual luciferase analysis of linc-AhRA primitive and AhR-binding site mutant promoter activity in BV2 cells 24 h after co-transfection of a linc-AhRA pro.-luc and TK-renilla, followed by the treatment of indirubin at the indicated concentration for another 6 h. **K,** Determination of HSV-1 titers in culture medium supernatants of gRNA-based AhR-deficient or control BV2 cells with HSV-1 infection (MOI 1) for the indicated number of hours using a plaque formation assay. **L,** AhR CUT&Tag-qPCR analysis of the enrichment of the fragment containing AhR binding sites within the linc-AhRA promoter using two primer sets for microglia acutely isolated from C57BL/6J mice with HSV-1 infection for 9 days. Data are representative of three independent experiments with n = 3 technical replicates (**A-E[left], G[top], I-L**) or n = 3 mice (**K**), three independent experiments (**E[right]-G[bottom]**), each symbol represents an individual or technical replicate(**A-E[left], G[top], I-J**) or an individual mouse (**K**) (shown as mean and s.d. in **A-D, E[right], G[top], I-L**), two-tailed unpaired Student's t-test (**A-B, D-E, G[top], K**), One-way ANOVA (and nonparametric analysis) (**C, I-J**), two-way ANOVA (**L**).

To elucidate the transcription factors (TFs) that regulate the expression of linc-AhRA, we predicted the TFs by using JASPAR to analyze the* linc-AhRA* promoter (-2,000 bps upstream of the transcription start site) 25. The potential TFs included AhR, AT-rich interacting domain-containing 3B (ARID3B), androgen receptor (AR), and ARID3A, while AhR had the highest probability score (**Table S3**). There were three AhR binding sites within the promoter of linc-AhRA. Next, we used validated small interfering RNAs (siRNAs) to knock down these TFs and found that only knockdown of AhR significantly reduced the upregulation of linc-AhRA upon HSV-1 infection (**Figure 3A**). Consistently, pre-treatment with the AhR inhibitor CH-223191, but not the AR inhibitor ODM-201, significantly suppressed the upregulation of linc-AhRA induced by HSV-1 infection (**Figure 3B**). Furthermore, treatment with an AhR agonist such as indirubin (**Figure 3C**) and L-kynurenine (**Figure S3A**) induced the expression of linc-AhRA, and other AhR targeted genes, such as *Cyp1b1* (encoding cytochrome P4501B1) and 2,3,7,8-tetrachlorodibenzo-p-dioxin (TCDD)-inducible poly (ADP-ribose) polymerase (*Tiparp*) in a dose-dependent manner. *Cyp1b1* and *Tiparp* were also induced by HSV-1 infection *in vitro* (**Figure 3D**) and *in vivo* (**Figure S3B**). The overexpression of AhR remarkably stimulated the expression of linc-AhRA (**Figure 3E**)**.** Immunofluorescence results further demonstrated that HSV-1 infection facilitated the nuclear import of AhRs, more evident in the late stage of infection (**Figure 3F**). We next generated AhR-deficient cells using CRISPR-Cas9 and found that AhR knockout significantly reduced the upregulation of linc-AhRA induced by HSV-1 infection (**Figure 3G**). Indeed, the published single-cell RNA sequence data indicated that microglia highly express *Ahr* compared to other cell types in the mouse CNS (**Figure S3C**)^26^, suggesting the involvement of AhR in the expression of linc-AhRA in microglia. An AhR-binding positive peak could also be observed in the linc-AhRA promoter as showed by the chromatin immunoprecipitation (ChIP) data for LPS-stimulated mouse macrophages as a present by the Cistrome Data Browser 27 (**Figure S3D**). Next, we cloned the original and AhR binding-site mutant linc-AhRA promoter into pGL4.11[luc2CP] to obtain a linc-AhRA-promoter-reporter luciferase and an AhR binding-site mutant promoter (**Figure 3H**). The dual-luciferase assay indicated that HSV-1 infection enhanced the linc-AhRA promoter activity, which was suppressed by the AhR inhibitor CH-223191 (**Figure 3I**). However, this behaviour was lost in linc-AhRA-promoter with the AhR-binding sites mutant (**Figure 3I**). Treatment with indirubin significantly activated the linc-AhRA promoter in a dose-dependent manner, which was also lost in the promoter with the AhR-binding sites mutant (**Figure 3J**). Furthermore, cleavage under targets and tagmentation (CUT&Tag) (**Figure S4**) performed on the primary microglia isolated from HSV-1 infected mice brain further confirmed that AhR binds to the promoter of linc-AhRA *in vivo* (**Figure 3K**). The titer of the progeny virus from HSV-1-infected AhR-deficient BV2 cells was lower than that from WT cells (**Figure 3L**).

### linc-AhRA negatively regulates the innate antiviral response of microglia

**Figure 4:**
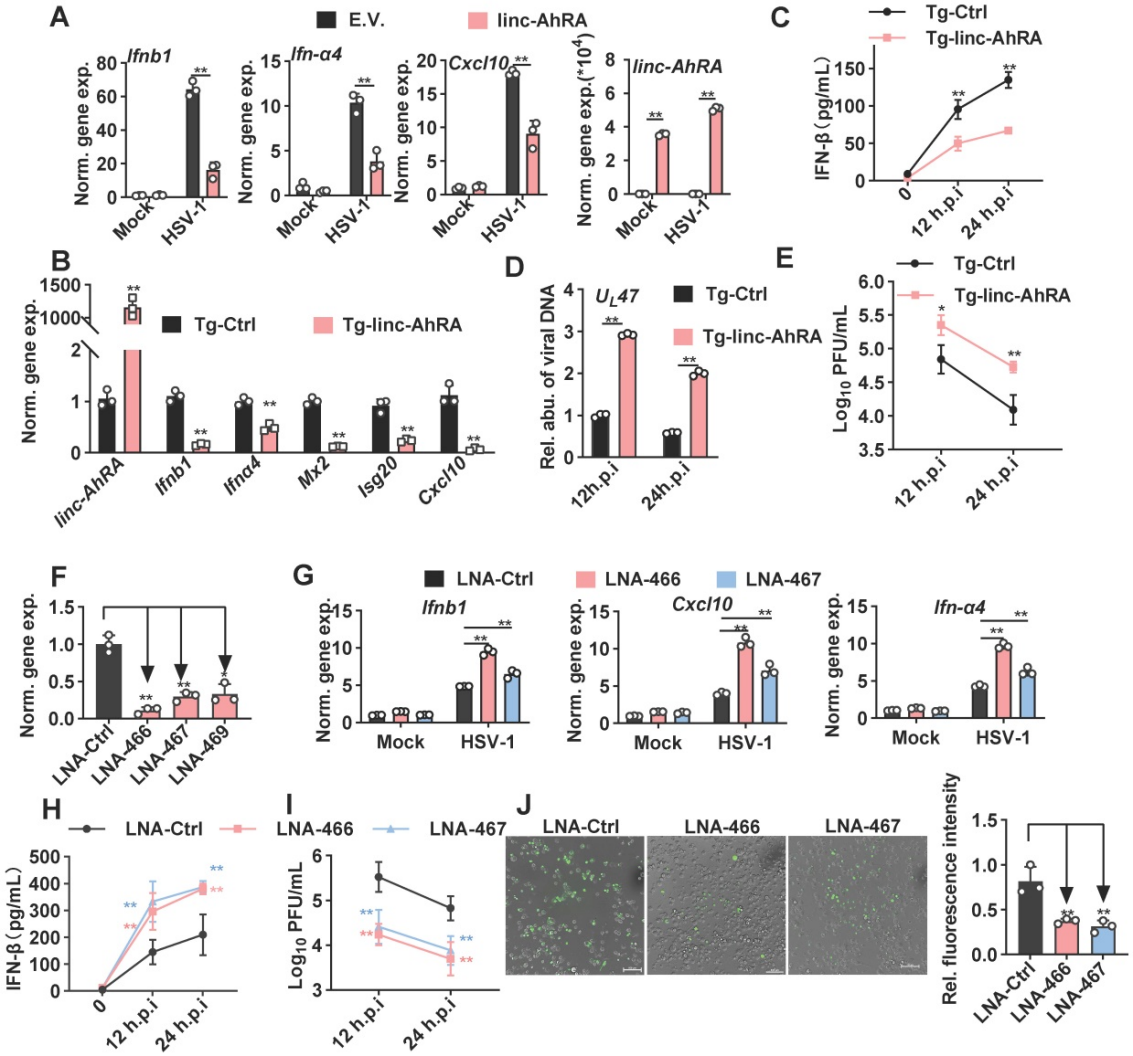
** linc-AhRA negatively regulates microglial innate antiviral response. A,** qPCR analysis of the expression of the indicated genes in BV2 cells transfected with linc-AhRA-expressing and E.V. plasmids, followed 48 h later by HSV-1 infection (MOI 1) for 12 h. **B,** qPCR analysis of the expression of the indicated genes in BV2 cells stably expressing linc-AhRA, followed by HSV-1 infection (MOI 1) for 12 h.** C,** ELISA of IFN-β in the supernatants of BV2 cells stably expressing linc-AhRA, followed by HSV-1 infection (MOI 1) for the indicated hours. **D,** Determination of viral DNA copies in BV2 cells stably expressing linc-AhRA with HSV-1 infection (MOI 1) for 24 h using qPCR. **E,** Determination of HSV-1 titers in culture medium supernatants of BV2 cells stably expressing linc-AhRA with HSV-1 infection (MOI 1) for the indicated duration using a plaque formation assay. **F,** qPCR analysis of the expression of linc-AhRA in BV2 cells transfected with LNA targeting linc-AhRA at different sites (LNA-466, LNA-467, and LNA-469) or control LNA for 24 h. **G,** qPCR analysis of the expression of the indicated genes in BV2 cells transfected with LNA targeting linc-AhRA at different sites (LNA-466 and LNA-467) or control LNA, followed 24 h later by HSV-1 infection (MOI 1) for 12 h. **H,** ELISA of IFN-β in the supernatants of BV2 cells transfected with LNA targeting linc-AhRA at different sites (LNA-466 and LNA-467) or control LNA, followed 24 h later by HSV-1 infection (MOI 1) for the indicated duration**. I,** Determination of HSV-1 titers in culture medium supernatants of BV2 cells transfected with LNA targeting linc-AhRA at different sites (LNA-466 and LNA-467) or control LNA, followed 24 h later by HSV-1 infection (MOI 1) for the indicated duration using a plaque formation assay. **J, Left,** Fluorescence microscopy images of viral replication (green) in BV2 cells with EGFP-HSV-1 (MOI 1) infection for 24 h, following the transfection of LNA targeting linc-AhRA (LNA-466 and LNA-467) or control LNA for 24 h. Scale bar, 100 μm**. right,** The quantitative result for relative EGFP fluorescence intensity obtained from three independent experiments. Data are representative of three independent experiments with n = 3 technical replicates (**A-I**), three independent experiments (**J**), each symbol represents an individual technical replicate (**A-D, F-G**) or one independent experiment (**J[right]**) (shown as mean and s.d. in **A-J[right]**), two-tailed unpaired Student's t-test (**A-B, D, F-G, J[right]**), two-way ANOVA (**C, E, H-I**).

We next investigated the effect of linc-AhRA on the innate antiviral response in BV2 cells and primary microglia, using expression plasmids and a lentiviral vector of linc-AhRA. We observed a significant reduction in the I-IFN* Ifnα4* and *Ifnb1* mRNAs in HSV-1-infected primary microglia with linc-AhRA overexpression at different times post-infection (**Figure S5A**). We also found that linc-AhRA significantly reduced the mRNA level of *Ifnα4*, *Ifnb1*, and *Cxcl10* during HSV-1 infection in BV2 cells (**Figure 4A**). Also, overexpression of linc-AhRA in BV2 cells led to a reduced level of the inflammatory factors including *Il-6* and *Tnf* by HSV-1 infection (**Figure S5B**)*.* Next, *linc-AhRA* was stably overexpressed in BV2 cells through lentiviral infection. The BV2 cells stably expressing linc-AhRA in the context of HSV-1 infection showed a reduced level of I-IFN and ISG mRNAs (**Figure 4B**). The RNA sequencing data also suggested that the FPKM value of the I-IFNs and several ISGs were significantly reduced in HSV-1-infected BV2 cells with a stable expression of linc-AhRA (**Figure S5C**)**.** Overexpression of linc-AhRA resulted in a remarkable reduction in IFN-β production upon HSV-1 infection as indicated by the enzyme-linked immunosorbent assay (ELISA) results (**Figure 4C**). Moreover, dual-luciferase assays indicated that linc-AhRA suppressed the HSV-1-mediated activation of IFN-β-stimulated interferon-stimulated response element (ISRE) luciferase activity but not the IFN-β-activated ISRE luciferase activity (**Figure S5D**), suggesting linc-AhRA only affected the expression of I-IFN but not its downstream signalling transduction. Consequently, linc-AhRA overexpression facilitated HSV-1 replication in BV2 cells at different times post-infection, as supported by HSV-1 genomic DNA copy quantification (**Figure 4D**) and the viral titer (**Figure 4E**). To rigorously examine the role of linc-AhRA in the innate antiviral response, we used antisense locked nucleic acid (LNA) GapmeRs against linc-AhRA. Endogenous linc-AhRA expression was successfully reduced by LNA GapmeRs No.466 and No.467 (**Figure 4F**). The knockdown of linc-AhRA by antisense LNA GapmeRs in BV2 cells resulted in a remarkable increment of I-IFN *Ifnα4*, *Ifnb1*, and *Cxc10* expression induced by HSV-1 infection, as compared to the LNA GapmeRs negative control group (**Figure 4G**)**.** The ELISA result indicated that the knockdown of linc-AhRA facilitated the production of IFN-β (**Figure 4H**). Moreover, the knockdown of linc-AhR by LNA GapmeRs significantly reduced the HSV-1 titer (**Figure 4I**). EGFP-HSV-1 infection in BV2 cells with linc-AhRA knockdown showed reduced fluorescence intensity (**Figure 4J**). Two LNA GapmeRs yielded similar results, demonstrating that the effect was highly unlikely to have been produced by a nonspecific LNA-dependent mechanism.

However, overexpression of linc-AhRA in Neuro-2a cells failed to affect the innate antiviral response upon HSV-1 infection (**Figure S6A**). Additionally, linc-AhRA showed no effect on viral genomic DNA copies (**Figure S6B**) and EGFP intensity (**Figure S6C**) in EGFP-HSV-1-infected Neuro-2a cells. Together, these results indicate that linc-AhRA mainly functions in the innate antiviral response in microglia but not in neurons. Given that microglial I-IFNs bestow antiviral capabilities to neurons, we next tested the effect of microglial linc-AhRA on the antiviral activity of neuron-like Neuro-2a cells against HSV-1 infection using microglia conditioned medium (MCM) (**Figure S6D**) Pre-treatment with MCM from CT-DNA-stimulated BV2 cells that stably expressed linc-AhRA led to a remarkable increase of the HSV-1 load in Neuro-2a cells (**Figure S6E**). By contrast, pre-treatment with MCM from CT-DNA-stimulated BV2 cells with LNAs targeting linc-AhRA conferred stronger antiviral activity to Neuro-2a cells than that from cells with control LNA (**Figure S6F**).

### linc-AhRA facilitates the degradation of TBK1 in a proteasome-dependent manner, resulting in the suppression of I-IFN production

**Figure 5:**
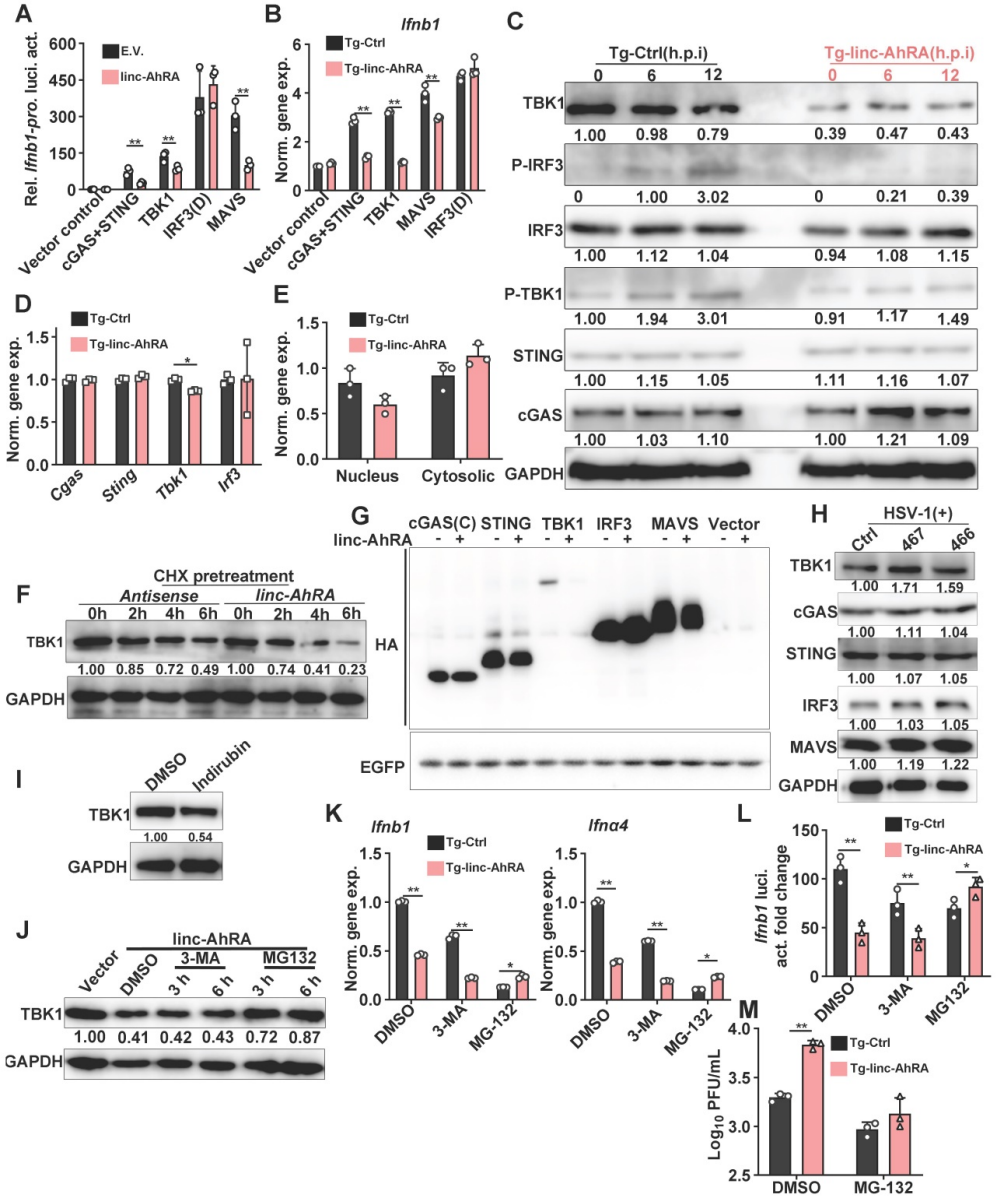
** linc-AhRA facilitates TBK1 degradation in a proteasome-dependent manner. A,** Dual luciferase analysis of IFN-β promoter activity in HEK 293T cells 36 h after co-transfecting *linc-AhRA* expressing plasmids (or E.V.), a firefly luciferase reporter (IFN-β-Luc), and TK-renilla with the indicated plasmids encoding cGAS and STING, TBK1, MAVS, and IRF3(D). **B,** qPCR analysis of *Ifnb1* expression in BV2 cells stably expressing linc-AhRA 36 h after transfection with the indicated plasmids encoding cGAS and STING, TBK1, MAVS, or IRF3(D). **C**, Immunoblot analysis of cGAS-STING signaling pathways in BV2 cells stably expressing linc-AhRA and control cells infected with HSV-1 (MOI 1) for the indicated hours. **D,** qPCR analysis of the expression of the indicated genes in BV2 cells stably expressing linc-AhRA and the corresponding control cells. **E,** qPCR analysis of the cytoplasmic and nuclear *Tbk1* level in BV2 cells stably expressing linc-AhRA. **F**, Immunoblot analysis of TBK1 level in BV2 cells transfected with linc-AhRA transcripts (2 μg) and antisense transcripts (2 μg) for the indicated number of hours, following pretreatment with CHX (10 μg/ml) for 6 h. **G,** Immunoblot analysis of HA in HEK 293T cells co-transfected with an equal amount of HA-fusion indicated factors expressing plasmids and pEGFP-C1 plasmids for 48 h. EGFP was present as a control for the transfection efficiency. **H**, Immunoblot analysis of cGAS-STING signaling pathways in BV2 cells with HSV-1 infection (MOI 1) for 24 h, following a transfection of the LNA control or LNA targeting linc-AhRA at different sites for 24 h. **I**, Immunoblot analysis of TBK1 level in BV2 cells with indirubin (50 μM) treatment for 9 h. **J**, Immunoblot analysis of TBK1 level in BV2 cells stably expressing linc-AhRA and treated with 3-MA (5 mM) and MG-132 (10 μM) for the indicated hours. **K,** qPCR analysis of *Ifnb1* and* Ifnα4* expression in BV2 cells stably expressing linc-AhRA or control cells treated with 3-MA (5 mM) and MG-132 (10 μM) in the context of HSV-1 (MOI 1) infection for 6 h. Data are normalized relative to the control group with the treatment of DMSO. **L,** Dual luciferase analysis of IFN-β promoter activity in control and linc-AhRA stably expressing BV2 cells 30 h after co-transfecting a firefly luciferase reporter (IFN-β-Luc) and TK-renilla, followed by treatment with 3-MA (5 mM) or MG-132 (10 μM) in the context of HSV-1 infection (MOI 1) for another 6 h. **M,** Determination of HSV-1 titers in culture medium supernatants of BV2 cells stably expressing linc-AhRA with HSV-1 infection (MOI 1) for 12 h in the presence of MG-132 (10 μM) for first 6 h. Data are representative of three independent experiments (**C, F, G-J**), three independent experiments with n = 3 technical replicates (**A-B, D-E, K-M**), each symbol represents an individual technical replicate (**A-B, D-E, K-M**) (shown as mean and s.d. in **A-B, D-E, K-M**), two-tailed unpaired Student's t-test (**A-B, D-E, K-M**).

Next, to investigate the mechanism of action of linc-AhRA in the modulation of the innate antiviral response, we examined the effect of linc-AhRA on viral entry and the amount of cyclic GMP-AMP synthase (cGAS)-binding viral DNA, which the cGAS-STING pathway uses to detect cytosolic DNA and induce an immune response. The flow cytometry results for reporter virus EGFP-HSV-1 and qPCR for viral DNA indicated that linc-AhRA did not reduce the viral entry amount (**Figure S7A-B**). Further, qPCR analysis for viral DNA isolated from cGAS immunoprecipitation demonstrated that the amount of DNA binding by cGAS was not affected by linc-AhRA (**Figure S7C-D**). Collectively, the inhibition of I-IFNs by linc-AhRA was not due to a reduction in the amount of the intracellular virus or cGAS-binding viral DNA. We next examined the level of linc-AhRA involvement in the molecular order of PRR-triggered signalling using co-transfected plasmids expressing the innate signalling components together with *Ifnb1* promoter-reporter plasmids. We found that the activation of *Ifnb1* promoter by cGAS+STING, mitochondrial antiviral signalling protein (MAVS), and TBK1 was significantly suppressed by linc-AhRA (**Figure 5A**). In contrast, linc-AhRA did not affect the constitutively active mutant IRF3(D)-mediated activation of *Ifnb1* promoter activity (**Figure 5A**). Consistently, linc-AhRA only reduced the induction of *Ifnb1* expression by cGAS+STING, MAVS, or TBK1, but not the constitutively active mutant IRF3(D) (**Figure 5B**). Indeed, the induction of *Ifnb1* in response to stimulation with CT-DNA and 5'ppp-dsRNA was also suppressed by linc-AhRA overexpression (**Figure S8A**), suggesting that linc-AhRA may regulate the cross-point of innate antiviral response against DNA and RNA virus. Moreover, the immunoblotting results suggested that the phosphorylation of IRF3 and TBK1 triggered by HSV-1 infection was attenuated in BV2 cells with stable expression of linc-AhRA (**Figure 5C**). Of note, a lower level of TBK1 protein was observed in BV2 cells with stable expression of linc-AhRA compared to the level in control cells, but did not significantly alter other factors except for cGAS (**Figure 5C**). Similar results were also observed in primary microglia (**Figure S8B**). These results suggest that linc-AhRA might limit I-IFN production through targeting TBK1.

To determine how linc-AhRA acts at the TBK1 level, we first investigated the effects of linc-AhRA on *Tbk1* mRNA expression. The results demonstrated that linc-AhRA had a minor effect on the expression of *Tbk1* (**Figure 5D**)**.** However, treatment with recombinant IFN-β largely restored the reduction of *Tbk1* mRNA induced by linc-AhRA (**Figure S8C**), implying that the effect of linc-AhRA on *Tbk1* mRNA may have resulted from the reduction of IFN-β. Nucleocytoplasmic trafficking is also an emerging manner for the modulation of targeted proteins 28. However, linc-AhRA did not disrupt the nucleocytoplasmic trafficking of *Tbk1* mRNA (**Figure 5E**). Together, there may be other mechanisms for TBK1 regulation by linc-AhRA. Interestingly, upon chlorhexidine (CHX) treatment to inhibit *de novo* protein synthesis, linc-AhRA transcripts obtained from *in vitro* transcription facilitated the degradation of TBK1 (**Figure 5F**), thereby supporting the regulatory role of linc-AhRA in TBK1 stability. Overexpression of linc-AhRA also reduced TBK1 expression from plasmids, with little effect on other IFN signaling factors, including cGAS, IRF3, and MAVS (**Figure 5G**). Of note, knockdown of linc-AhRA with LNA GapmeRs increased the protein level of TBK1 but had a minor effect on other factors (**Figure 5H**)**.** Treatment with the AhR agonist indirubin also induced the degradation of TBK1 in BV2 cells (**Figure 5I**). We next used the autophagy inhibitor 3-MA and proteasome inhibitor MG-132 to elucidate the pathway of TBK1 degradation induced by linc-AhRA. MG-132, but not 3-MA, blocked the loss of TBK1 protein induced by linc-AhRA overexpression (**Figure 5J**). Treatment with MG-132 also restored the linc-AhRA-mediated reduction of *Ifnb1* and* Ifnα4* mRNA expression (**Figure 5K**) and the impairment of *Ifnb1* promoter activity induced by HSV-1 infection (**Figure 5L**). Further, MG-132 treatment abolished the inhibition effect of linc-AhRA overexpression on HSV-1 replication (**Figure 5M**). Collectively, proteasome, but not autophagy, is involved in TBK1 degradation mediated by linc-AhRA.

**Figure 6:**
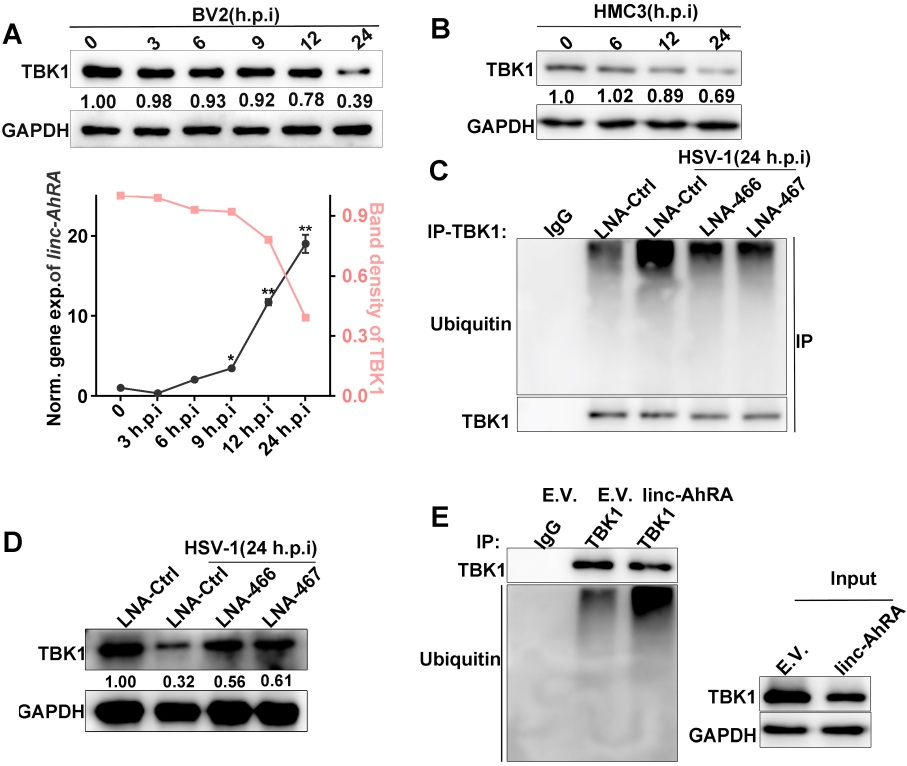
** HSV-1 infection led to a degradation of TBK1 at the late stage of infection, which were partially mediated by linc-AhRA. A, Top**: Immunoblot analysis of TBK1 level in BV2 cells with HSV-1 infection (MOI 1) for the indicated number of hours. **Bottom**: Band density of TBK1 in the immunoblot result presented on top and qPCR analysis of *linc-AhRA* expression in BV2 cells with HSV-1 infection (MOI 1) for the indicated number of hours. **B,** Immunoblot analysis of TBK1 level in HMC3 cells with HSV-1 infection (MOI 1) for the indicated number of hours. **C,** Immunoblot analysis of ubiquitinated TBK1 level using immunoprecipitation with anti-TBK1 antibody in BV2 cells with LNA-mediated knockdown of linc-AhRA followed by HSV-1 infection (MOI 1) for 24 h. **D,** Immunoblot analysis of TBK1 level in BV2 cells with the same treatment as in (**C**). **E,** The ubiquitination of TBK1 in linc-AhRA-overexpressing BV2 cells was assessed by immunoprecipitation with anti-TBK1 antibody. Data are representative of three independent experiments (**A[top]- E**), three independent experiments with n = 3 technical replicates (**E[bottom]**) (shown as mean and s.d. in **A[bottom]**), One-way ANOVA (and nonparametric analysis) (**A[bottom]**).

A reduced level of TBK1 protein was also observed at the late phase of HSV-1 infection when linc-AhRA was significantly upregulated (**Figure 6A**). A degradation of TBK1 was observed in HMC3 cells in the late stage of HSV-1 infection (**Figure 6B**). Moreover, the knockdown of linc-AhRA restored the HSV-1-enhanced ubiquitination and degradation of TBK1 (**Figure 6C-D**). Indeed, overexpression of linc-AhRA is sufficient to increase the ubiquitination of TBK1 in the absence of HSV-1 infection (**Figure 6E**). These results indicated that the accumulation of linc-AhRA leads to a degradation of TBK1 at the late stage of HSV-1 infection in microglia.

### linc-AhRA acts as a scaffold and enhances TRIM27-TBK1 interaction

**Figure 7:**
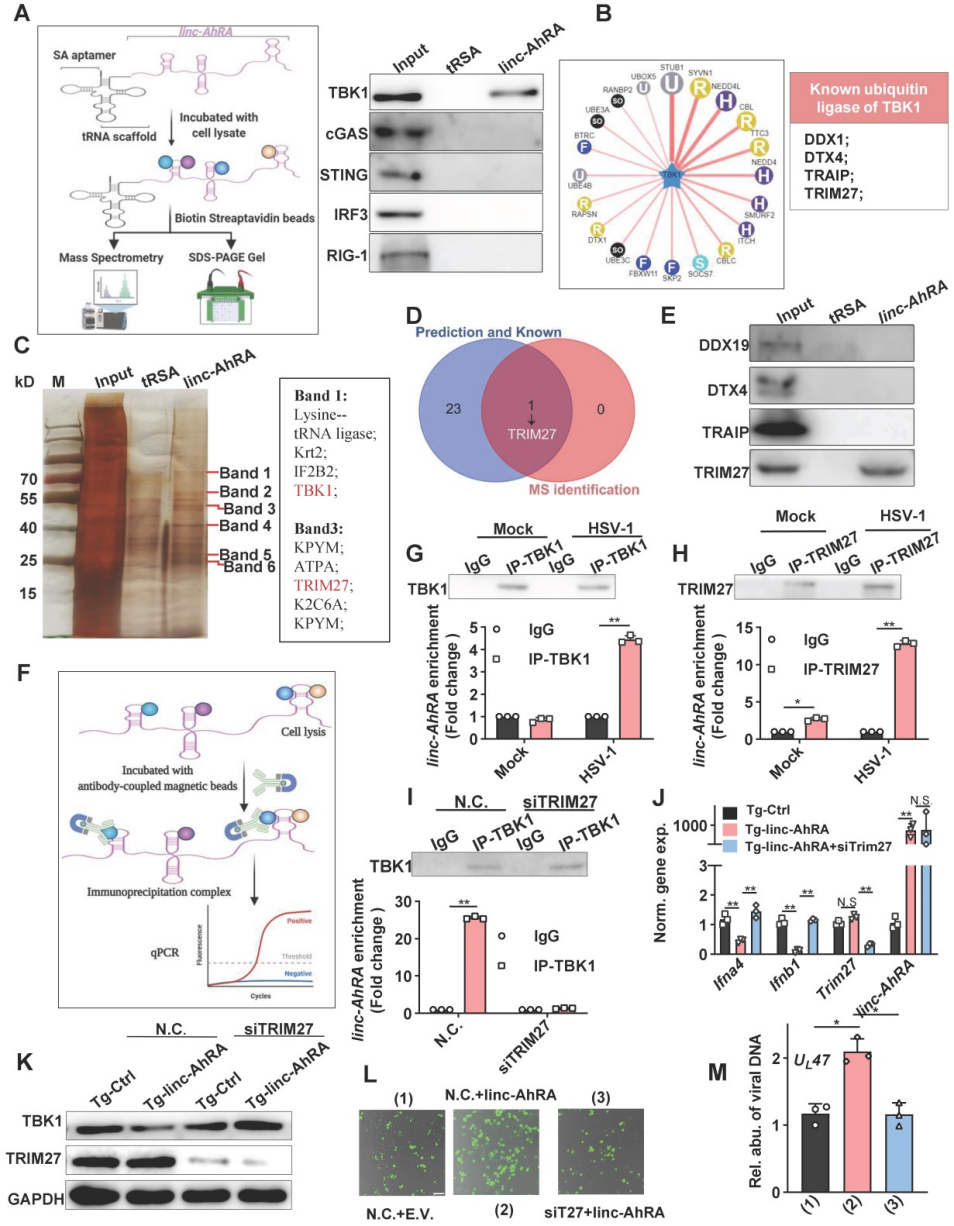
** linc-AhRA interacts with TRIM27 and HSV-1 infection induces the binding of linc-AhRA to TBK1. A, Left**: Schematic illustration of tRSA RNA pull-down assay; **right**: Immunoblot analysis of the indicated factors in tRSA and tRSA-*linc-AhRA* pull-down enrichment. **B, Left**: Flower plot of the predicted E3 ligases targeting TBK1, analyzed using Ubibrowser;** right**: List of known E3 ligases of TBK1. **C, Left**: SDS-PAGE with silver nitrate staining analysis of proteins co-purified with linc-AhRA. The specific bands (label in red) within the tRSA-linc-AhRA group were extracted for mass spectrometry (MS) analysis; **right:** The partial list of proteins identified by MS. **D,** Venn diagram analysis for the overlapped E3 ligase of TBK1 both in the list as presented in (**B**) and in MS-identified factors as in (**C**). **E,** Immunoblot analysis of the indicated E3 ligase in tRSA and tRSA-*linc-AhRA* pull-down enrichment. **F,** Schematic illustration of RNA immunoprecipitation assay. **G, Top**: Immunoblot analysis of TBK1 immunoprecipitated by TBK1-specific antibody or immunoglobulin G (IgG) from BV2 cells uninfected or infected with HSV-1 (MOI 1) for 24 h;** bottom**: RIP-qPCR analysis of linc-AhRA immunoprecipitated from same samples as in (**top**). **H, Top**: Immunoblot analysis of TRIM27 immunoprecipitated with TRIM27-specific antibody or IgG from BV2 cells with the same treatment as in (**G**);** bottom**: RIP-qPCR analysis of linc-AhRA immunoprecipitated from same samples as in (**H**). **I, Top**: Immunoblot analysis of TBK1 immunoprecipitated with TBK1-specific antibody or IgG from BV2 cells transfected with TRIM27 siRNA for 48 h then infected with HSV-1 (MOI 1) for 24 h;** bottom**: RIP-qPCR analysis of linc-AhRA immunoprecipitated by TBK1-specific antibody or IgG from samples as in (**top**). **J,** qPCR analysis of the expression of the indicated genes in BV2 cells stably expressing linc-AhRA and transfected with TRIM27 siRNA for 48 h then infected with HSV-1 (MOI 1) for 6 h. **K,** Immunoblot analysis of TBK1 and TRIM27 levels in BV2 cells stably expressing linc-AhRA and transfected with TRIM27 siRNA for 48 h. **L,** Fluorescence microscopy images of HSV-1 (green) replication in BV2 cells co-transfected with linc-AhRA and TRIM27 siRNA for 48 h, followed by EGFP-HSV-1 infection (MOI 2) for 24 h. Scale bar, 100 μm. **M,** Determination of viral DNA copies using qPCR in BV2 cells stably expressing linc-AhRA with a transfection of TRIM27 siRNA for 48 h infected with HSV-1 (MOI 2) for 24 h. Data are representative of three independent experiments (**A, C, E, G-I[top], K, L**), three independent experiments with n = 3 technical replicates (**G-I[bottom], J, M**), each symbol represents an individual technical replicate (**G-I[bottom], J, M**) (shown as mean and s.d. in **G-I[bottom], J, M**), two-tailed unpaired Student's t-test (**G-I[bottom], J, M**).

Next, we attempted to determine how linc-AhRA induces the degradation of TBK1 and first tested the possibility that linc-AhRA modulated the expression of its neighbour genes. The brain acid-soluble protein 1 (*Basp1*) was identified as the only neighbour coding gene with a distance of less than 100,000 bps. However, overexpression of linc-AhRA did not affect the mRNA expression of Basp1 in BV2 cells with or without HSV-1 infection (**Figure S9A-B**). Therefore, it is unlikely that linc-AhRA functions by regulating the expression of BASP1. Indeed, linc-AhRA still led to the degradation of TBK1 after blocking protein synthesis (above), suggesting the degradation of TBK1 may not be caused by the downstream genes of linc-AhRA. Therefore, we next focused on whether linc-AhRA interacts with TBK1. Given the addition of the tRNA scaffold to a streptavidin aptamer (tRSA) increased binding efficiency by ∼10-fold 29, tRSA-linc-AhRA or tRSA transcripts were incubated with BV2 cell lysates, and the enriched protein samples were analyzed with an immunoblotting assay (**Figure 7A, left**). The immunoblotting results showed that linc-AhRA interacted with TBK1, but not with other crucial factors, including MAVS, cGAS, STING, and IRF3 (**Figure 7A, right**). Further, considering that linc-AhRA enhanced the ubiquitination of TBK1 (above), we speculated that linc-AhRA might bind to the specific E3 ubiquitin ligase of TBK1. We predicted the E3 ubiquitin ligase using Ubibrowser and summarized the known E3 ligases of TBK1 (DEAD-box helicase 19A (DDX19A), Deltex E3 Ubiquitin Ligase 4 (DTX4), TRAF-interacting protein (TRIP), and TRIM27) 10, 11, 13, 30, 31 (**Figure 7B**). To identify the E3 ligases that interact linc-AhRA, the specific bands observed in the linc-AhRA pull-down enrichment indicated by silver staining were analyzed with mass spectrometry (MS) (**Figure 7C**). Among these known and predicted E3 ubiquitin ligases of TBK1, only TRIM27 was identified in tRSA-linc-AhRA enriched lysates using MS (**Figure 7C-D**). The result was further confirmed by immunoblotting (**Figure 7E**). Next, TRIM27-TBK1 interactions were confirmed in BV2 cells at the late phase of HSV-1 infection but not in the absence of HSV-1 infection (**Figure S10A**). Additionally, confocal microscopy imaging revealed a co-localization of TRIM27 and TBK1 in the cytoplasm and nuclear export of TRIM27 in the late stage of infection (**Figure S10B**). However, the RNA immunoprecipitation (RIP)-qPCR results (**Figure 7F**) indicated that TBK1-linc-AhRA interactions were only observed in microglia with HSV-1 infection at the late stage, but not in microglia without HSV-1 infection (**Figure 7G**). In contrast, TRIM27 interacted with linc-AhRA in an HSV-1-infection-independent manner, and HSV-1 infection strengthened the linc-AhRA-TRIM27 interaction (**Figure 7H**). Indeed, a time course matched expression profile indicated that *Trim27* and *linc-AhRA* upregulated significantly in the late phase of HSV-1 infection in primary microglia (**Figure S10C**). By contrast, both of which were not upregulated significantly in the neuron in the context of HSV-1 infection (**Figure S10D**). Apoptotic is the central manner of cell death during HSV-1 infection 32. The apoptotic Caspases were known to suppress I-IFN production in numerous manners 33-35. We next used Z-VAD-FMK, a general Caspase inhibitor, to investigate whether the inhibition effect of linc-AhRA on I-IFN expression were involved Caspase-mediated cell death. The qRT-PCR result indicated that the overexpression of linc-AhRA induced by HSV-1 or mediated by plasmids still reduced *Ifnb1* expression in the presence of Z-VAD-FMK (**Figure S10E-F**). Such a result excluded the possibility that the decrease in *Ifnb1* expression in the context of linc-AhRA overexpression or in the late phase of HSV-1 infection is due to cell death. Moreover, knockdown of TRIM27 using validated siRNA was sufficient to facilitate the expression of I-IFNs and ISGs under HSV-1 infection (**Figure S10G**). We next investigated whether linc-AhRA binds to TBK1 before interacting with TRIM27. Notably, the RIP assay demonstrated that linc-AhRA failed to bind to TBK1 in *Trim27*-knockdown BV2 cells at the late stage of HSV-1 infection (**Figure 7I**). Additionally, the knockdown of TRIM27 restored the linc-AhRA-mediated reduction of I-IFN expression (**Figure 7J**) and the degradation of TBK1 (**Figure 7K**). Moreover, the results for the fluorescence intensity of reporter virus EGFP-HSV-1 suggested that knockdown of TRIM27 inhibited the effect of linc-AhRA on HSV-1 replication (**Figure 7L**) and the viral DNA copy numbers (**Figure 7M**). Collectively, linc-AhRA may regulate the innate antiviral response by modulating the TRIIM27-TBK1 interaction.

**Figure 8:**
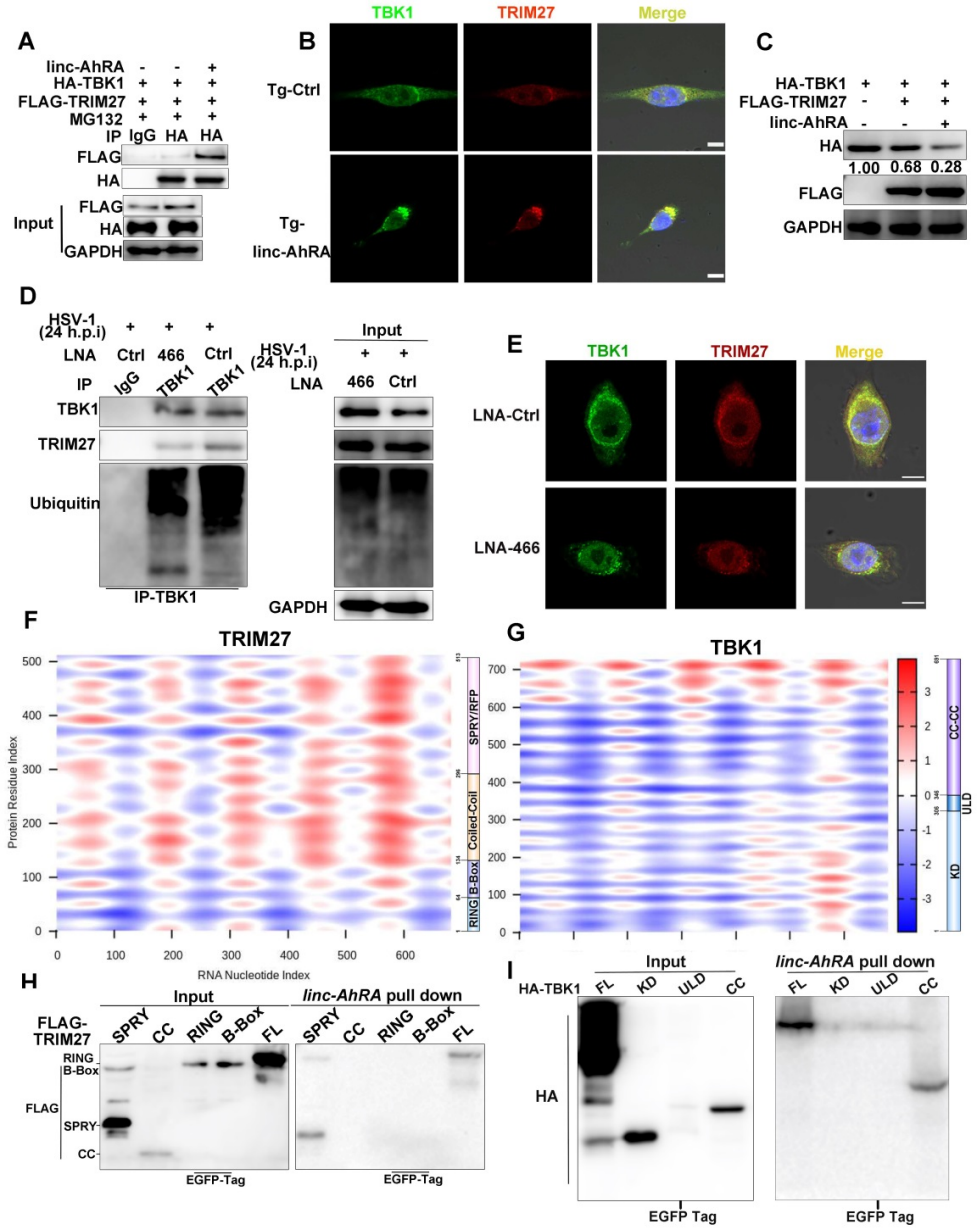
** linc-AhRA acts as a scaffold enhancing TBK1-TRIM27 interaction to facilitate the TRIM27-mediated degradation of TBK1. A,** Co-immunoprecipitation (Co-IP) analysis of the TBK1-TRIM27 interactions in HEK 293T cells co-transfected with FLAG-TRIM27, HA-TBK1, and linc-AhRA for 42 h, followed by MG-132 treatment (10 μM) for another 6 h. **B,** Confocal microscope-based fluorescence analysis of the co-localization of TBK1 (green) and TRIM27 (red) in BV2 cells stably expressing linc-AhRA with MG-132 treatment (10 μM) for 6 h. Nuclei were labeled with DAPI (blue). Scale bars, 10 μm. n = 30 cells per group were subjected to analysis. **C,** Immunoblot analysis of HA and FLAG level in HEK 293T cells co-transfected with FLAG-TRIM27, HA-TBK1, and linc-AhRA for 48 h. **D,** Co-IP analysis of the TBK1-TRIM27 interaction and TBK1 ubiquitination in BV2 cells transfected with LNA-Ctrl or LNA-466 for 24 h and infected with HSV-1 (MOI 1) for 24 h.** E,** Confocal microscope-based fluorescence analysis of the co-localization of endogenous TBK1 (green) and TRIM27 (red) in BV2 cells transfected with LNA-Ctrl or LNA-466 for 24 h and infected with HSV-1 (MOI 1) for another 24 h in the presence of MG-132 (10 μM) for the final 6 h. Nuclei were labeled with DAPI (blue). Scale bars, 10 μm. n = 30 cells per group were subjected to analysis. **F,** Heatmap indicating the interaction propensity between linc-AhRA and TRIM27 analyzed by catRAPID expression. **G,** Heatmap indicating the interaction propensity between linc-AhRA and TBK1 analyzed by catRAPID expression. **H,** RNA pull-down assay of the binding of linc-AhRA to FLAG-tagged truncations of TRIM27 using the lysates of HEK 293T cells transfected with the corresponding plasmids as in (**B**) for 48 h. **I,** RNA pull-down assay of the binding of linc-AhRA to HA-tagged truncations of TBK1 using the lysates of HEK 293T cells transfected with the corresponding plasmids as in (**A**) for 48 h. Data are representative of three independent experiments (**A-E, H-I**).

We next investigated the effect of linc-AhRA on TBK1-TRIM27 interactions. We found that linc-AhRA overexpression strengthened TBK1-TRIM27 interactions (**Figure 8A**) without affecting the mRNA expression and HSV-1 infection-induced nuclear export of TRIM27 (**Figure S10H-I**). In addition, linc-AhRA did not affect the protein level of TRIM27 (**above**). The confocal images showed that linc-AhRA enhanced the co-localization of TBK1 and TRIM27 (**Figure 8B**). The TRIM27-mediated degradation of TBK1 was further enhanced by linc-AhRA (**Figure 8C**). Next, we examined whether HSV-1 infection-induced TBK1 degradation was associated with the linc-AhRA-mediated enhancement of TBK1-TRIM27 interaction. Knockdown of linc-AhRA by LNA reduced the HSV-1-induced ubiquitination of TBK1 and TBK1-TRIM27 interactions (**Figure 8D**). The confocal images also demonstrated that knockdown of linc-AhRA attenuated the HSV-1-induced co-localization of TBK1 with TRIM27 at the late stage of infection (**Figure 8E**). Together, linc-AhRA enhanced the TRIM27-TBK1 interaction, thereby facilitating the ubiquitination and degradation of TBK1.

To characterize the TBK1-TRIM27 interactions in detail, we constructed plasmids expressing an HA-tagged domain of TBK1 and a FLAG-tagged domain of TRIM27 (**Figure S11A-B, top**). Immunoprecipitation and immunoblot analyses showed that TRIM27-TBK1 interactions were mediated by their respective coiled-coil domains (**Figure S11A-B, bottom**), which is consistent with a previous study 10. Next, we used catRAPID expression to predict the propensity of interaction between TBK1 or TRIM27 and linc-AhRA. catRAPID expression is a tool for predicting the interaction propensity of a protein-RNA pair and reports the interaction score and the discriminative power 36, 37. The interaction matrix calculated by catRAPID expression indicated that TRIM27, as an RNA-binding protein (RBP) recently recognized 38, showed an excellent propensity for interacting with linc-AhRA in general (**Figure 8F**). In contrast, only the coiled-coil domain of TBK1 showed strong interaction propensity with linc-AhRA (**Figure 8G**). We next performed tRSA RNA pull-down assays using cell lysates containing various truncated fragments of TRIM27 or TBK1 to determine the region of interaction between TBK1 or TRIM27 and linc-AhRA. Partially consistent with the predictions obtained from catRAPID graphic, the RNA pull-down assay indicated that the binding of TRIM27 to linc-AhRA was mediated by the SPla/Ryanodine receptor (SPRY) domain of TRIM27 (**Figure 8H**)**.** In contrast, the coiled-coil domain of TBK1 mediated its interaction with linc-AhRA (**Figure 8I**), which is entirely consistent with the analysis results from catRAPID graphic.

### The conserved 117nt fragment is required for the enhancement of TRIM27-TBK1 interaction mediated by linc-AhRA

**Figure 9:**
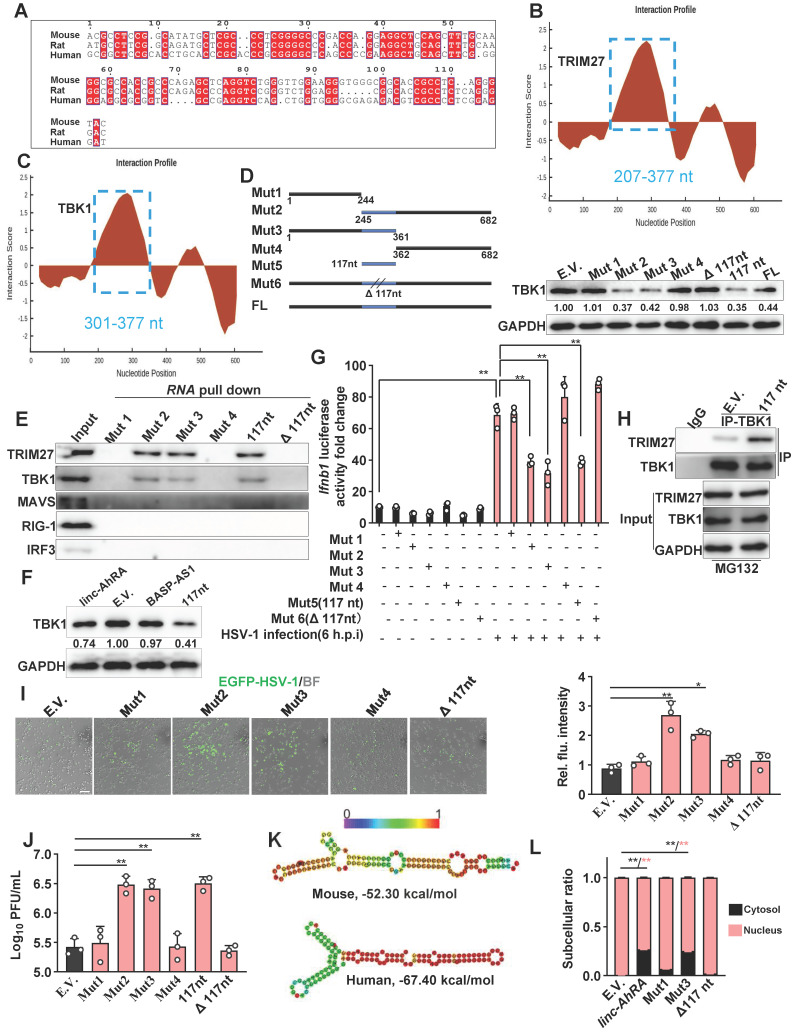
** The conserved 117nt fragment is required for the enhancement of TRIM27-TBK1 interaction mediated by linc-AhRA. A,** The sequence alignment of 117nt conserved fragment within linc-AhRA. **B-C**, The interaction profile for linc-AhRA and TRIM27 (**B**) or TBK1 (**C**) analyzed by catRAPID fragments (http://service.tartaglialab.com/page/catrapid_group).** D,** Schematic illustration of constructed deletion mutants of linc-AhRA (**left**) and immunoblot analysis of TBK1 level in BV2 cells transfected with the plasmids expressing the indicated deletion mutants of linc-AhRA for 48 h (**right**). **E,** Immunoblot analysis of the indicated factors in the pull-down enrichment of the corresponding tRSA-linc-AhRA deletion mutants. **F,** Immunoblot analysis of TBK1 level in HMC3 cells with a transfection of the plasmids expressing the indicated lncRNAs for 48 h. **G,** Dual luciferase analysis of IFN-β promoter activity in HEK 293T cells with the co-transfection of *linc-AhRA* deletion mutants expressing plasmids as indicated, a firefly luciferase reporter (IFN-β-Luc) and TK-renilla for 30 h and infected with HSV-1 (MOI 1) for another 6 h. **H,** Co-IP analysis of the TBK1-TRIM27 interaction in HEK 293T cells co-transfected with FLAG-TRIM27, HA-TBK1, and 117nt for 42 h, followed by MG-132 treatment (10 μM) for another 6 h. **I, Left:** Fluorescence microscopy images of viral replication (green) in BV2 cells with EGFP-HSV-1 (MOI 1) infection for 24 h, following a transfection of the indicated linc-AhRA deletion mutant or empty control for 48 h. Scale bar, 100 μm**. Right**: The quantitative result for relative EGFP fluorescence intensity obtained from three independent experiments. **J,** Determination of viral titers in BV2 cells transfected with plasmids expressing the indicated truncation of linc-AhRA for 48 h, followed by HSV-1 infection (MOI 1) for 24 h. **K,** Secondary structure of *linc-AhRA* conserved 117nt fragment (top) and human homology (bottom) with minimum free energy predicted by the Vienna RNA web server. The corresponding ensemble free energy is also labeled.** L,** qPCR analysis of cytoplasmic and nuclear ratios of *linc-AhRA* deletion mutants in BV2 cells with HSV-1 infection (MOI 1) for 24 h, following a transfection of the indicated linc-AhRA deletion mutant or empty control for 48 h. Data are representative of three independent experiments (**D[right]-F, H-I**), three independent experiments with n = 3 technical replicates (**G, J, L**), each symbol represents an individual technical replicate (**G, J**) or one independent experiment (**I[right]**)(shown as mean and s.d. in **G, I[right], J, L**), two-tailed unpaired Student's t-test (**G, I[right], J, L**).

To determine whether an equivalent lncRNA is located in the human genome corresponding to mouse linc-AhRA, we performed a conservation analysis for linc-AhRA. Although the Placental mammal Basewise Conservation track identified by PhyloP among 60 vertebrates indicated that linc-AhRA is not generally conserved, further analysis limited to mice, rats, and humans revealed a 117nt (245-361nt) conserved fragment within linc-AhRA (**Figure 9A** and **Figure S12A**). Based on this conserved fragment, we used the RACE assay to obtain an equivalent lncRNA named *BASP-AS1* located in the human genome corresponding to mouse *linc-AhRA* in HMC3 cells (**Figure S12B**). The results indicated that BASP-AS1 is a spliced transcript of 3327nt with a poly-A tail (**Table S4**). Although HSV-1 infection stimulated the expression of BASP-AS1 (**Figure S12C**), BASP-AS1 did not affect the microglial innate antiviral response or the virus titers in HMC3 cells (**Figure S12D-E**). However, the conserved 117nt fragment increased the HSV-1 titers (**Figure S12E**) and suppressed the expression of *IFNB1* in the HMC3 cells (**Figure S12F**). Moreover, GTEx (release version 6) indicated that brain tissues had the highest abundance of BASP-AS1 among all human tissues (**Figure S12G**), suggesting a potential implication of BASP-AS1 in CNS.

To characterize the region within linc-AhRA that interacts with TBK1 and TRIM27 in detail, we used catRAPID fragments to calculate the interaction propensities of linc-AhRA with TBK1 and TRIM27. catRAPID fragments predict RNA-protein interaction propensities based on a procedure that involves the division of polypeptide and nucleotide sequences into fragments. The results showed that a fragment (207-377nt) within linc-AhRA had the highest score for binding to TRIM27 (**Figure 9B**) and TBK1 (**Figure 9C**) (**Table S5** and **Table S6**). Of note, this region contained the 117nt conserved fragments (245-361nt). Based on the 117nt conserved fragment, we constructed a series of deletion mutant fragments for linc-AhRA, specifically Mut 1, Mut 2, Mut 3, Mut 4, 117nt, and Δ117nt (**Figure 9D** and **Figure S13**). We found that only the linc-AhRA truncated fragments containing the 117nt conserved fragment could degrade TBK1 in murine microglia (**Figure 9D**). Further, RNA pull-down analysis revealed that linc-AhRA mutants lacking the conserved 117nt fragment could no longer bind to TBK1 and TRIM27 and degrade TBK1 (**Figure 9E**). Interestingly, both linc-AhRA and the 117nt conserved fragment, but not BASP-AS1, can degrade TBK1 in human microglia-like HMC3 cells (**Figure 9F**). Further, the linc-AhRA mutants lacking the conserved 117nt fragment could not inhibit HSV-1-infection-activated IFN-β luciferase, whereas linc-AhRA fragments containing the conserved 117nt fragment retained this function (**Figure 9G**).

Indeed, the 117nt conserved fragment of linc-AhRA was sufficient to enhance the interactions between TBK1 and TRIM27 in murine microglia (**Figure 9H**). Moreover, the fragments containing the 117nt region, but not those without the 117nt fragment, facilitated HSV-1 infection in BV2 cells, confirmed by the EGFP-reporter virus fluorescence intensity (**Figure 9I**) and viral titers (**Figure 9J**)**.** Given the difference in function between *linc-AhRA* and* BASP-AS1*, we next determined whether the conserved 117nt fragment is related to this difference. Based on their secondary structure under the minimum free energy, it is noteworthy that the 117nt fragment is hidden in the secondary structure of BASP-AS1, leading to a failure of exposure and the formation of two continuous loops (**Figure S14A**). In contrast, the 117nt fragment within linc-AhRA is completely exposed, resulting in the successful formation of two continuous loops (**Figure S14B**). This difference may lead to the functional difference between linc-AhRA and BASP-AS1. Moreover, the 117nt fragment of linc-AhRA and its human ortholog had highly similar structures in their “Y” forms (**Figure 9K**), implying their similar function. Interestingly, we also isolated the subcellular fraction RNA and unexpectedly found that loss of the 117nt fragment led to an impairment of the nuclear export of linc-AhRA (**Figure 9L**), suggesting the conserved 117nt fragment within linc-AhRA is crucial for their nuclear export.

### Microglial linc-AhRA KI mice are susceptible to HSV-1 infection and exhibit an impaired innate antiviral response

To explore the role of microglial linc-AhRA upon neurotropic virus infection* in vivo*, we established a mouse model with microglia-specific linc-AhRA knock-in (KI) at the Rosa26 locus (**Figure S15A**). *Cx3cr1*-CreERT2 mice, in which the *Cx3cr1* promoter drives the expression of Cre recombinase fused to an estrogen ligand-binding domain, were mated with Cre-dependent linc-AhRA KI mice to obtain tamoxifen (TAM)-induced microglia-specific linc-AhRA KI mice (**Figure S15B**). To determine the efficiency of linc-AhRA KI, we isolated the neurons, astrocytes, microglia, peritoneal macrophages, and bone marrow-derived macrophages (BMDMs). We found that both microglia (29-60-fold change) and BMDMs (2-5-fold change) from microglia-specific linc-AhRA KI mice expressed high levels of linc-AhRA following subcutaneous injection of TAM (**Figure S16A**). We also extracted the RNA from various tissues and found that only the brain expressed high levels of linc-AhRA following TAM treatment in microglial linc-AhRA KI mice (**Figure S16B**). In particular, the cortex and BS isolated from linc-AhRA KI mice showed higher levels of linc-AhRA than those from control mice (**Figure S16C**). We also investigated astrocytes in the CNS using histological immunofluorescence analysis. We found that linc-AhRA KI did not affect the morphology of glial fibrillary acidic protein (GFAP^+^) astrocytes in all of the brain regions investigated (**Figure S16D**). Flow cytometry analysis results showed comparable microglial clusters (CD11b^+^CD45^low^) in linc-AhRA KI and control mice (**Figure S16E**). We next performed a thorough histopathological analysis of different brain regions in microglia-specific linc-AhRA KI mice and the corresponding control counterparts. We found that the microglia-specific linc-AhRA KI mice exhibited no abnormalities in their CNS gross anatomy (**Figure S16F**). Histological analyses of the five principal organs showed no significant differences between the linc-AhRA KI and control mice (**Figure S16G**). To test whether other phenotypes existed in microglia-specific linc-AhRA KI mice, we compared the body weights and sizes and found no significant differences (**Figure S16H-I**).

**Figure 10:**
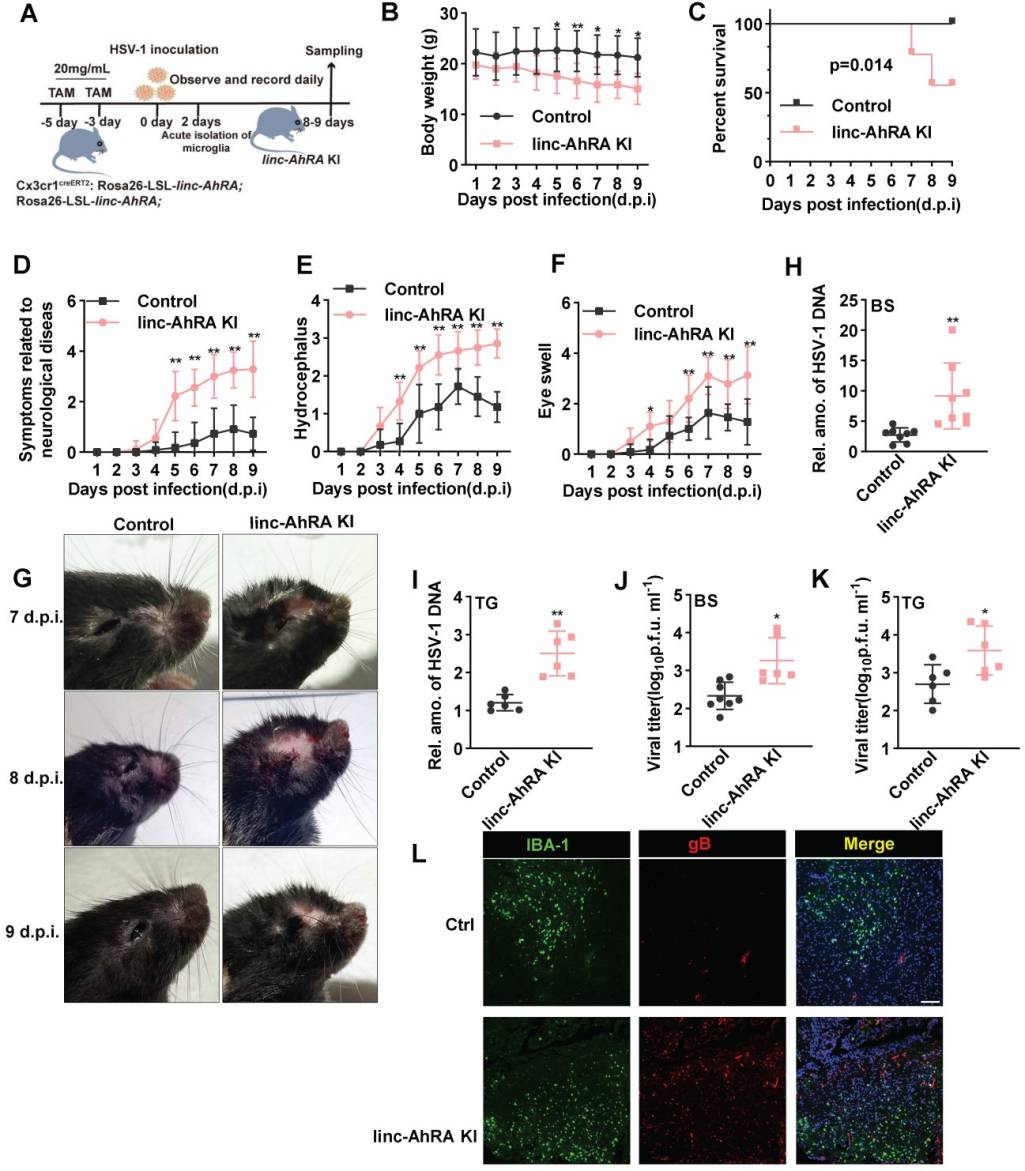
** Microglial linc-AhRA KI mice are susceptible to HSV-1 infection. A,** The schematic protocol for inducing microglial linc-AhRA KI and establishing the HSE mice model. Microglial linc-AhRA KI mice were intranasally inoculated with HSV-1 (1×10^7^ PFU/mouse). On subsequent days, the mice were weighed daily (**B**), survival was recorded daily (**C**), and symptoms related to neurological diseases (**D**), hydrocephalus (**E**), and eye swelling (**F**) were scored daily. n= 9-12 mice per group (**A-F**). **g,** Photographic records of the right eyes and brain symptoms from mice with corresponding d.p.i., n = 7-12 mice per group. **H-I,** Determination of viral DNA copies in homogenized brain stem (BS) (**H,** n = 8 mice per group) and trigeminal ganglia (TG) (**I,** n = 6 mice per group) at 9 d.p.i. using qPCR. Determination of HSV-1 titers in BS (**J**) and TG (**K**) at 9 d.p.i. using a plaque formation assay, n = 6 mice per group. **L**, Tissue sections from BS of control and microglial linc-AhRA KI mice with HSV-1 infection at 9 d.p.i were stained with HSV-1-gB and IBA-1, n = 7 mice per group. Scale bars, 100 μm. Data are representative of three independent experiments (**B-L**) and each symbol represents an individual mouse (**H-K**) (shown as mean and s.d. in **B-F, H-K**). Two-way ANOVA (**B, D-F**), two-tailed unpaired Student's t-test for **H-K**, log-rank (Mantel-Cox) test for **C**.

Next, we challenged the microglia-specific linc-AhRA KI mice and the corresponding control mice with HSV-1 in the brain to establish an HSE model (**Figure 10A**). Of note, the microglia-specific linc-AhRA KI mice lost weight rapidly after intranasal infection with HSV-1 (**Figure 10B**) and showed higher mortality than the control mice (**Figure 10C**). Moreover, we observed severe disease development in microglia-specific linc-AhRA KI mice, as demonstrated by disease scores reflecting neurological symptoms (**Figure 10D**), hydrocephalus (**Figure 10E**), and eye swelling (**Figure 10F**), as well as images showing the mice brain (**Figure 10G**). Given an enrichment of viral genomic DNA in the trigeminus (TG) and BS in HSV-1-infected mice (**Figure S17A**), which were in accordance with previous studies 39, 40, we focused on analyzing the viral load in these sections. The results indicated that the HSV-1 genomic DNA copy numbers were significantly higher in the BS (**Figure 10H**) and TG (**Figure 10I**) of microglia-specific linc-AhRA KI mice than in their control counterparts. Similarly, a higher virus titer was also observed in the BS (**Figure 10J**) and TG (**Figure 10K**) from microglia-specific linc-AhRA KI mice than in those from control mice. Immunofluorescence with an anti-HSV-1 gB indicated that microglia-specific linc-AhRA KI mice had more HSV-1 virions than the corresponding control mice in the BS, especially in the IBA-1^+^ microglia (**Figure 10L**).

**Figure 11:**
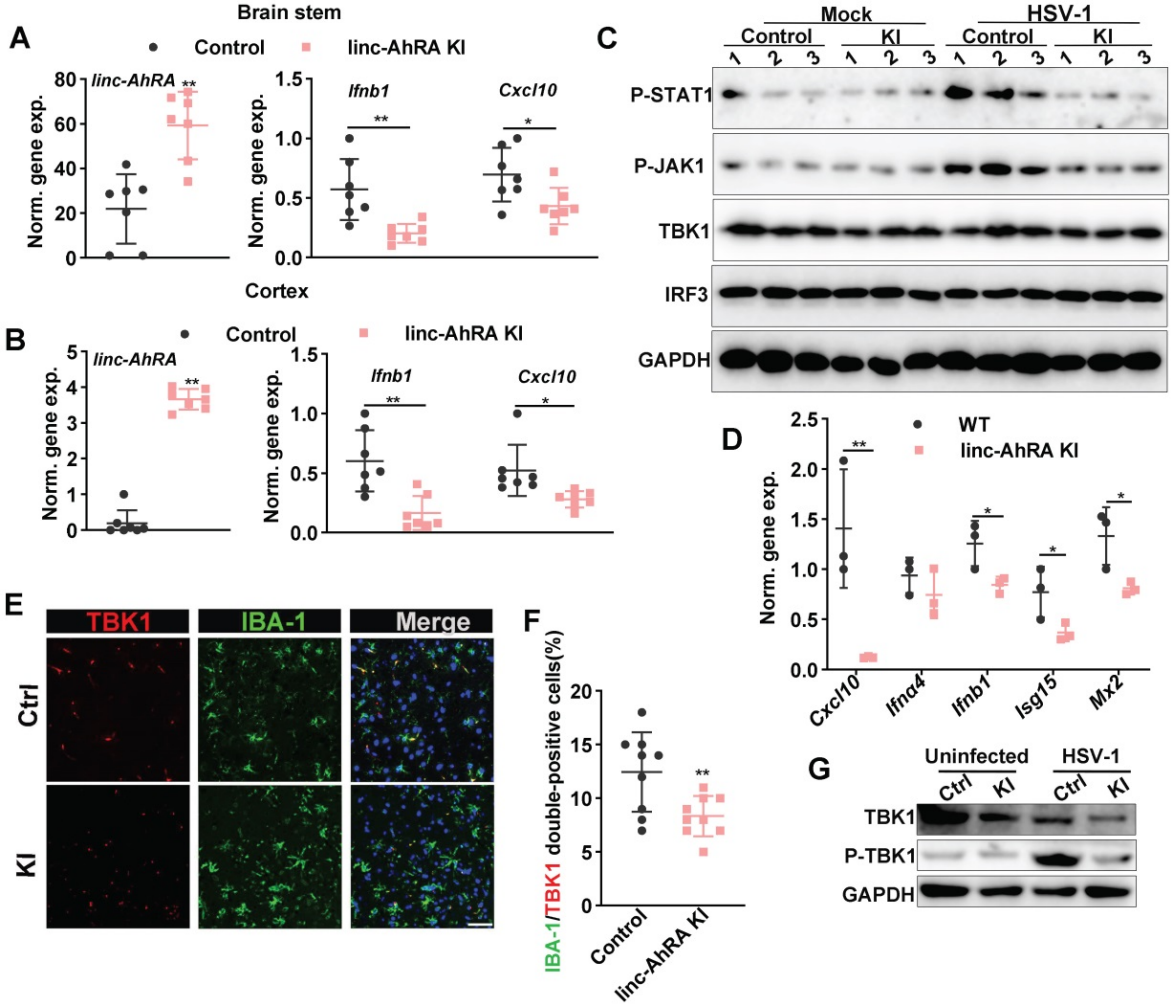
** Microglial linc-AhRA KI mice exhibited an impaired innate antiviral response upon HSV-1 challenge. A,** qPCR analysis of *linc-AhRA*, *Ifnb1*, and *Cxcl10* expression in BS isolated 8 days after infection with HSV-1, n = 7 mice per group. **B,** qPCR analysis of *linc-AhRA*, *Ifnb1*, and *Cxcl10* expression in cerebral cortex isolated 8 days after infection with HSV-1, n = 7 mice per group. **C,** BS with or without HSV-1 infection were isolated on day 8 after infection; homogenized and immunoblotted with the indicated antibodies. The typical results from three mice per group are shown. **D,** qPCR analysis of the expression of the indicated genes in microglia acutely isolated 2 days after infection with HSV-1, n = 3 mice per group. **E,** Tissue section from the BS of control and microglial linc-AhRA KI mice isolated 2 days after infection with HSV-1 were stained with TBK1 and a microglia-specific marker IBA-1 (n = 9 mice per group), white arrows indicate the IBA-1/TBK1 double-positive cells; Scale bars, 50 μm. **F**, Quantification of IBA-1/TBK1 double-positive cells. **G**, Immunoblotting analysis of the indicated genes in microglia acutely isolated 8 days after infection with HSV-1. Data are representative of three independent experiments (**A-G**), each symbol represents an individual mouse (**A-B, D, F**) (shown as mean and s.d. in **A-B, D, F**). Two-tailed unpaired Student's t-test (**A-B, D, F**).

We next determined the role of microglial linc-AhRA in the innate antiviral response of mice brains against HSV-1 infection. We first analyzed the activated morphology of microglia at different times points following HSV-1 infection. We found that an activated microglia morphology appears at 2 days post-infection (d.p.i) in wild-type (WT) mice with HSV-1 infection, and the activation is most evident at 8 d.p.i. (**Figure S17B**). Therefore, we isolated the brain tissue at 8 d.p.i. to analyze the level of I-IFN and ISGs. We found a reduction of* Ifnb1* and *Cxcl10* expression in the BS (**Figure 11A**) and cortex (**Figure 11B**) from microglia-specific linc-AhRA KI mice. The immunoblotting results indicated a reduced TBK1 and STAT1 phosphorylation level in BSs from microglia-specific linc-AhRA KI mice (**Figure 11C**)**.** To determine the effect of linc-AhRA on the microglial innate antiviral response *in vivo* at an early stage of infection, we also performed acute isolation of microglia at 2 d.p.i. to analyze the level of I-IFN and ISGs. As demonstrated by the qRT-PCR results, microglia acutely isolated from microglia-specific linc-AhRA KI mice showed a reduced level of *Ifnb1*, *Cxcl10*, *Isg15*, and *Mx2* against HSV-1 intranasal infection at 2 d.p.i (**Figure 11D**).

Further, the tissue immunofluorescence assay demonstrated that the microglial linc-AhRA KI mice showed a decreased level of TBK1 in IBA-1^+^ microglia in the BS, as reflected by the number of IBA-1 and TBK1 double-positive cells (**Figure 11E-F**). The microglia acutely isolated from microglia-specific linc-AhRA KI mice exhibited reduced phosphorylation of TBK1 and a level of TBK1 (**Figure 11G**). Together, microglial linc-AhRA negatively regulated the innate antiviral response in the CNS upon neurotropic HSV-1 infection, possibly due to the reduced TBK1 level in microglia.

## Discussion

Microglia originate from embryonic yolk sacs and are disparate from non-CNS macrophages and other CNS macrophages 4, suggesting a difference between microglia and other macrophages, as revealed by some studies 41. The current understanding of the innate antiviral response regulatory network is mainly focused on non-CNS macrophage cells. In contrast, the regulation mechanisms of innate antiviral response in microglia remain largely unknown. Identifying the key molecules involved in the innate antiviral response of microglia against neurotropic herpesvirus contributes to a better understanding of innate immune homeostasis in the CNS. We identified linc-AhRA as a novel and abundant lncRNA in cultured microglial cell lines and primary microglia. linc-AhRA was markedly upregulated in microglia upon neurotropic virus HSV-1 infection and influenza virus and CVB infection. The upregulation depended on virus-activated AhR signalling. linc-AhRA initiates a feedback loop that suppresses antiviral innate immune responses in microglia, facilitating the infection of neurotropic herpesvirus in murine microglia. Indeed, IFN-β is recently reported to activate AhR signalling 42. We found that numerous innate stimuli can induce the expression of linc-AhRA, suggesting linc-AhRA-mediated negative feedback is an intrinsic pathway inhibiting I-IFN initiated by the host but not a specific virus. Nevertheless, such a negative feedback loop is exploited by the virus to escape innate antiviral response. Indeed, AhR can be activated by numerous viruses, including SARS-CoV-2 and Zika virus 42-44. AhR signalling is involved in the innate antiviral response and viral pathogenesis 42-44. However, the role of AhR-activated non-coding transcripts in the innate antiviral response remains unknown. linc-AhRA expressed by neurons did not exhibit a similar function, suggesting that linc-AhRA has a specific function in microglia. Indeed, published single-cell RNA-sequencing indicated that microglia, but not neurons, expressed high levels of AhR 26, 45, which may lead to the loss of a phenotype for the AhR-activated non-coding gene in neurons during neuronal development. A low level of TRIM27 in neurons may also result in this phenotype. Nevertheless, I-IFNs generated by microglia orchestrate the innate antiviral response for neurons 2, 6, suggesting a potential implication of linc-AhRA in the innate antiviral response in the CNS.

The nuclear lncRNA usually modulates the expression of targeted genes in the nucleus 46. However, linc-AhRA transcripts obtained from *in vitro* transcription reduced the stability of TBK1 in the presence of a protein synthesis inhibitor CHX (**Figure 5F**). Moreover, the accumulated linc-AhRA induced by HSV-1 can export to cytosol at which linc-AhRA interacted with TBK1, a cytosolic factor (**Figure 2H-I, Figure 7, and Figure 9**). Further, the mutant lacking conserved 117nt fragment did not show a similar function with the entire length of linc-AhRA or locate efficiently at cytosol (**Figure 9**). The function of linc-AhRA in the innate antiviral response depended on its direct action with TBK1 but not its effect on the expression of targeted genes in the nucleus. However, the possibility that linc-AhRA regulates cGAS and STING is excluded because linc-AhRA cannot bind cGAS and STING and viral DNA amount bound by cGAS cannot be affected by linc-AhRA. Indeed, the mRNA is not an ideal level for the activity regulation of TBK1 as a kinase. linc-AhRA acts as a scaffold that enhances TRIM27-TBK1 interactions to increase the TRIM27-mediated ubiquitin modification of TBK1 and subsequent degradation in a proteasome-dependent manner to suppress the innate antiviral response of microglia. Notably, TRIM27 is a recently discovered RBP mediated by the SPRY domain 19, 38, and the role of its RNA-binding activity in the innate antiviral response, as well as its lncRNA interactors, has yet to be determined. Our study showed that linc-AhRA binds to the SPRY domain of TRIM27 and the coiled-coil domain of TBK1 in HSV-1-infected microglia. In the absence of neurotropic HSV-1 infection, linc-AhRA binds to TRIM27 but not TBK1. At the late stage of HSV-1 infection, linc-AhRA binds to both TBK1 and TRIM27. The knockdown of TRIM27 reduces the linc-AhRA-TBK1 interactions and the function of linc-AhRA in the microglial innate antiviral response, suggesting linc-AhRA-TBK1 interactions depend on TRIM27. Knockdown of linc-AhRA also restored the degradation of TBK1 induced by HSV-1 infection in the late stage, echoing the previous findings that HSV-1 facilitates the degradation of TBK1 in the late stage of infection 47.

Ubiquitination is ideal for regulating a biological process that requires a rapid response, such as virus infection. TBK1 is a crucial kinase in the signal transduction of the innate antiviral response 9 and has been reported to undergo ubiquitination, which precisely controls its activity 9-11. In detail, the E3 ligase DTX4 mediates the K48-linked polyubiquitination and degradation of TBK1 11. TRIM27 induces TBK1 degradation via K48-linked ubiquitination at Lys251 and Lys372 10. TRIP negatively regulates the antiviral response by promoting the proteasomal degradation of TBK1 48. LncRNAs are increasingly recognized as crucial factors in host-virus interaction via numerous mechanisms, especially in the innate antiviral response 19. However, relatively few lncRNAs target TBK1 49, and the role of lncRNAs in the ubiquitination-based degradation of the key factors of innate antiviral response remains obscure. Given that lncRNA also participates in the ubiquitination-mediated degradation of its targeted protein 50, it is not unexpected that lncRNAs are involved in the ubiquitination-based stability modulation of innate antiviral factors. How does linc-AhRA serve as a scaffold to place TRIM27 and TBK1 in the proper positions? Are there specific sequences or secondary structures that determine the translocation of these proteins or the binding of linc-AhRA to specific proteins? We can exclude the possibility that the nuclear export of TRIM27 and the TBK1-TRIM27 interaction depend on linc-AhRA for the following reasons: 1) TRIM27 interacted with TBK1 in HEK 293T cells without linc-AhRA, but linc-AhRA overexpression significantly strengthened the interaction; 2) linc-AhRA did not affect the nuclear export of TRIM27 induced by HSV-1 infection. Further studies are necessary to determine whether linc-AhRA serves as a scaffold and functions via conformational changes. The structural characterization of protein-RNA complexes is a promising approach that should uncover detailed information regarding this interaction.

Although the human homologous lncRNA of linc-AhRA cannot modulate the innate antiviral response of microglia, we identified a 117nt conserved functional fragment within linc-AhRA in murine microglia. Indeed, numerous studies have demonstrated that the RNA-binding domain SPRY is required for the function of ubiquitination of most TRIM family members 38, 51, 52. Based on this perspective, it is fascinating to investigate whether human endogenous lncRNAs mediate TBK1 degradation, similar to mouse linc-AhRA. RIP sequencing for TBK1 or TRIM27 immunoprecipitation would identify human lncRNAs that can be recognized by TBK1 or TRIM27. The 117nt conserved fragment mediates the function of linc-AhRA, supported by the following results: 1) The deletion mutants containing the 117nt fragment, but not those lacking 117nt, could inhibit the innate antiviral response of murine microglia, thereby facilitating neurotropic virus HSV-1 replication; 2) The deletion mutants containing the 117nt fragment, but not those lacking 117nt, could bind both TBK1 and TRIM27 as well as lead to TBK1 degradation; 3) The 117nt fragment is sufficient to enhance TRIM27-TBK1 interaction; 4) Human and mouse 117nt fragments have a highly similar secondary structure with a “Y” form; 5) The human ortholog 117nt within BASP-AS1 cannot be exposed as demonstrated by the secondary structure, leading to a failure of BASP-AS1 function in the innate antiviral response of human microglia. Indeed, the catRAPID prediction indicated that the fragment within linc-AhRA with a strong propensity for TBK1 and TRIM27 interaction contained the conserved 117nt region. Although numerous evolutionarily conserved lncRNAs have been identified, only a few lncRNAs contain conserved functional fragments 20, 21. Identifying the functional fragment that endows lncRNAs with cellular activities would be beneficial for the development of nucleic acid-based therapeutics 53-55. Therefore, the 117nt-based drugs would be ideal candidates for treating autoimmune diseases characterized by high levels of I-IFNs, such as systemic lupus erythematosus (SLE) and Aicardi-Goutières syndrome 56. Further, although linc-AhRA is a nuclear-resident lncRNA in the steady-state, we found that HSV-1 infection induced the nuclear export of linc-AhRA at the phase when linc-AhRA is highly expressed. Indeed, the increased linc-AhRA expression from plasmids is also partially distributed to the cytoplasm. The linc-AhRA deletion mutants lacking the 117nt functional fragment could not be exported to the cytoplasm. Therefore, the 117nt conserved fragment directs the nuclear export of linc-AhRA and determines the binding capacity with TBK1 and TRIM27. However, although linc-AhRA binds to TRIM27, linc-AhRA cannot affect the nuclear export of TRIM27, possibly because not all linc-AhRAs interact with TRIM27 or not all TRIM27 can bind to linc-AhRA. Nevertheless, the cytoplasmic translocation of linc-AhRA is required for the regulation of the innate antiviral response. Indeed, although nuclear lncRNAs are overall more abundant, they are less stable than their cytoplasmic counterparts 57. However, the precise mechanism of linc-AhRA cytoplasm translocation has yet to be determined. In summary, our study provides novel insights into the mechanism underlying the negative regulation of I-IFN production in microglia by a lncRNA, which viruses may exploit for immune evasion.

## Materials and Methods

### Key resources

Detailed information regarding viruses, cell lines, antibodies, chemicals (TargetMol, Selleck, Macklin, and InvivoGen), and software can be obtained from **Table S7**. Mouse IFN-β ELISA Kit was purchased from 4A Biotech Co., Ltd (Cat #CME0116).

### RNA isolation and quantitative real-time PCR (qPCR)

Total RNA from cultured cells with the indicated treatments was isolated using TRIzol Reagent (TIANGEN, #DP405). One microgram of RNA per sample was used for cDNA synthesis with the PrimeScript RT Reagent using the gDNA Eraser Kit (Takara, #RR047A). qPCR assays were performed in a CFX96 Touch Real-Time PCR Detection System (Bio-Rad) using a TB Green Premix Ex Taq II Kit (Takara, #RR820A) following the manufacturer's instructions. The gene expression levels were normalized to the internal housekeeping gene* Gapdh.* All qPCR procedures, including the design of the primers, validation of PCR conditions, and quantification, were performed according to the MIQE guidelines 58. The gene-specific primers are listed in **Table S8**. For an absolute quantification of *linc-AhRA*, the known copies of linc-AhRA obtained from *in vitro* transcription were subjected to six 10-fold serial dilutions to create a standard curve to monitor RNA purification and amplification. The amplified transcripts of linc-AhRA were quantified using the comparative Ct method.

### High-throughput lncRNA-seq and data analysis

Total RNA of primary microglia with and without HSV-1 infection was isolated with the TRIzol Reagent. RNA degradation and contamination were monitored by using 1% agarose gels. The RNA purity and integrity were checked using the NanoPhotometer® spectrophotometer (IMPLEN, CA, USA) and the RNA Nano 6000 Assay Kit with the Bioanalyzer 2100 system (Agilent Technologies, CA, USA), respectively. The RNA concentration was measured using the Qubit® RNA Assay Kit in Qubit® 2.0 Flurometer (Life Technologies, CA, USA). Three micrograms of RNA per sample was used as the input material and ribosomal RNA (rRNA) was removed using the Epicentre Ribo-zero rRNA Removal Kit (Epicentre, USA). Afterward, the rRNA-free residues were cleaned further using ethanol precipitation. Subsequently, sequence libraries were established using the NEBNext Ultra Directional RNA Library Prep Kit for Illumina (NEB, Ipswich, MA, USA) according to the manufacturer's instructions and purified using the AMPure XP system. The quality of the sequence libraries was assessed on the Agilent Bioanalyzer 2100 system. The clustering of index-coded samples was performed on a cBot Cluster Generation System using the TruSeq PE Cluster Kit v3-cBot-HS (Illumina) following the manufacturer's recommendations. After cluster generation, the libraries were sequenced on an Illumina Hiseq 4000 platform (Novogene, Beijing, China) with 150-bp paired-end reads. Cuffdiff (v2.1.1) was used to calculate the FPKMs for each sample's lncRNAs and coding genes 59. Cuffdiff provides statistical routines for determining differential expression in digital transcript or gene expression data using a model based on a negative binomial distribution 59. The genes that exhibited a fold change > 4 with adjusted P <0.05 were filtered as differentially expressed genes. All raw data were uploaded to GEO (GSE167015).

### Plasmids, siRNA, and transfection

All plasmids were constructed for this study, unless otherwise stated. In detail, the coding sequences of cGAS, STING, TBK1, IRF3, MAVS, and TRIM27 were amplified from the cDNAs of BV2 cells infected with HSV-1 for 12 h and inserted into pCMV-HA vector with the ClonExpress Ultra One Step Cloning Kit (Vazyme, #C115-01), except for TRM27, which was cloned into p3XFLAG-CMV10 plasmids. The mimic activated IRF3(D) form was amplified using HA-IRF3 plasmids and then cloned into pCMV-HA plasmids with the Mut Express® MultiS Fast Mutagenesis Kit V2 (Vazyme, #C215-01). All lncRNA-expressing plasmids were constructed by TSINGKE (Beijing, China). The deletion mutants were amplified from full-length expression plasmids and cloned into the corresponding plasmid using the ClonExpress Ultra One Step Cloning Kit. The primers for the plasmids constructed in this study are listed in **Table S9**. The validated siRNAs were obtained from Sigma (https://www.sigmaaldrich.com/singapore.html) and synthesized by GenePharma (Shanghai, China). Detailed information regarding the siRNAs is presented in **Table S10.** The transfection of plasmids in most cells was performed with jetPEI reagents (Polyplus, #PT-114-15) except for the transfection of difficult cells, such as BV2 cells, which was performed using the TransIT-Jurkat reagent (Mirus, #2120). For siRNA transfection, the INTERFERin reagent (Polypus Transfection, #PT-409-10) was used following the manufacturer's instructions.

### Dual-luciferase reporter (DLR) assay

Consistent with our prior study 60, the DLR assay was performed according to the manufacturer's instructions for the dual-luciferase assay Kit (Promega, #E1910). Briefly, the *Ifnb1-*minimal promoter and linc-AhRA promoter were amplified from the genome of BV2 then cloned into the promoter-less vector pGL4.11[luc2p] encoding the firefly luciferase to generate the corresponding promoter-driven luciferase reporter plasmids. Next, HEK 293T or BV2 cells cultured in 24-well plates were transfected with the corresponding factor expression plasmids and the reporter plasmid pGL4.11[luc2p]-*Ifnb1* promoter or pGL4.11[luc2p]-*linc-AhRA* promoter as well as the internal control vector pRL-TK-Renilla luciferase. The luciferase activity was tested at 36 h post transfection using the DLR Assay System and GloMax 20/20 luminometer (Promega). The relative luciferase activity (RLA) was calculated as the ratio of firefly luciferase activity to *Renilla* luciferase activity and presented as the fold change relative to the RLA in basic vector-transfected cells. The primers used to construct the reporter plasmid are presented in **Table S11**.

### Rapid amplification of cDNA ends and idedntification of full-length lncRNA

5' and 3' RACE assays were performed using the SMARTer RACE 5'/3' Kit (Clontech, #634858) according to the manufacturer's instructions. Briefly, total RNA isolated from HSV-1-infected primary microglia was used to synthesize the first-strand cDNA. Given that linc-AhRA lacks a polyadenylated tail, a poly(A) tail was added using Poly(A) Polymerase (Takara, #2180A) for the 3'-first-strand cDNA synthesis. The synthesis proceeded using the protocol provided in the manufacturer's instructions. The 5'-first-strand cDNA was synthesized with random primers. Primers used for the linc-AhRA RACE assay were designed based on the known sequence obtained from the RNA-sequence results. Based on the conserved fragment within linc-AhRA, we also designed primers for the BASP-AS1 RACE assay to obtain full-length BASP-AS1. Primers for the RACE assay are presented in **Table S12**.

### Viral titer determination, DNA purification, and quantification

Viral titers were determined using the plaque assay 60. Briefly, Vero cells were seeded onto 24-well plates then infected with media containing HSV-1 at a series of dilutions for 2 h. Subsequently, the virus inoculum was removed, added overlay medium containing 1% methylcellulose, and the cells were incubated for 72 h. The final samples were harvested and fixed with 4% paraformaldehyde (PFA) then stained with 1% crystal violet. Plaque numbers were counted and recorded. Viral DNA isolation and quantification were performed based on our prior study 60. Briefly, cells with the corresponding treatment were repeated frozen at -80 °C and thawed three times. Afterward, the viral DNA was isolated using the TIANamp Virus DNA/RNA kit (Transgene, Beijing, China, #ER201-01). The purified viral DNA were quantified using a qRT-PCR assay.

### Identification of conserved fragment

Highly conserved fragments within linc-AhRA in different species were obtained from UCSC (http://genome.ucsc.edu), Multiple Sequence Alignment by CLUSTALW (https://www.genome.jp/tools-bin/clustalw), and ESPript 3.0 (https://espript.ibcp.fr/ESPript/cgi-bin/ESPript.cgi). In detail, the linc-AhRAs were subjected to placental mammal basewise conservation analysis among 60 vertebrates using the PhyloP tool in UCSC. Next, we checked the alignment block and obtained the detailed sequence information regarding the respective alignment block with the highest score. The alignment blocks in mice, rats, and humans were subjected to multiple sequence alignment with CLUSTALW and ESPript to identify the conserved fragment.

### RNA subcellular isolation

RNA subcellular isolation was performed based on our previous study with minor modifications 60. Briefly, cells were harvested and washed with ice-cold phosphate-buffered saline (PBS) twice. After centrifugation at 1000 g for 5 min, removed the supernatant. The cell pellets were resuspended using 100 μL 0.1% v/v NP40 in RNase-free water containing 10 mM Ribonucleoside Vanadyl Complex (RVC) (NEB, #S1402S) and a protease and phosphatase inhibitor cocktail (Beyotime, #P1045) by pipetting gently. After centrifugation at 5000 × g for 30 sec, the supernatant was collected and labelled as the cytoplasmic fraction. The pellet was washed five times with 200 μL ice-cold 0.1% NP40-PBS with centrifugation at 1000 × g for 5 min each time. The cells were centrifuged at 5000 × g for 30 s for the last time, discarded the supernatant, and labelled the pellet as the nucleus. Next, RNA was extracted from the nucleus and cytoplasm fractions using the EasyPure RNA Kit (Transgen, #ER101) following the manufacturer's instructions.

### Mouse primary microglia isolation and culture

Mouse primary microglia were purified according to the protocol previously described with minor modifications 8, 61, 62. In brief, mice brain was dissected from neonatal mice (1-3 days) and washed three times with 5 mL ice-cold wash media (low-glucose Dulbecco's Modified Eagle Medium (DMEM) supplemented with 1% penicillin-streptomycin) to remove blood. Next, both the olfactory bulb and cerebellum were removed. The meningeal layer was carefully stripped to avoid contamination of the monocytes in the blood vessels and damage to the cortices. The cortices were digested with 3 mL 0.25% trypsin-ethylenediaminetetraacetic acid (EDTA; Life Technologies, #25200072) for 20 min and dispersed to a single-cell level by passing through a cell strainer (70 µm) following the stop digestion by DMEM containing 10% FBS. The cell suspension was centrifuged at 500 × g for 5 min at 4 °C and resuspended with growth medium then cultured at 37 °C in humidified 5% CO_2_ and 95% air on poly-D-lysine (10 µg/mL) (Beyotime, #C0312)-precoated 75 cm^2^ cell culture flasks. The medium was half-replaced every 4-5 days. After the cells reached confluence (8-10 days), the astrocytes and microglia were isolated by mild trypsinization with 0.05% Trypsin-EDTA (Life Technologies, #25300054) for 5 min 8, 61. In detail, treatment of the confluent mixed glial cultures with 0.05% Trypsin-EDTA resulted in the detachment of an intact layer of cells containing almost all of the microglia and leaving behind highly enriched astrocytes. Over 95% of the microglia cultures obtained by the digestion of trypsinization were positive for IBA-1. The cellular yield is 4.0×10^5^ microglia/neonatal mouse. More than 85% of the remaining cells were astrocytes, as determined by staining with GFAP (not shown). After the isolation procedure, the attached microglia were allowed to recover for 24 h, and the cells were further plated as required for the specific experiments. To obtain sufficient primary cells, we collected the primary cells from five mice as a mixture to seed plate.

### Acute isolation of microglia from adult mice

Mononuclear cells were isolated from the CNS as previously described with minor optimizations 8, 63-65. Antibody-coupled microbeads (Miltenyi Biotec, #130-093-634) were used for magnetic affinity cell sorting to isolate CD11b^+^ microglia following previous studies 61, 66, 67. Briefly, murine cerebral cortices were isolated, cut into pieces no smaller than 1 mm^3^ and incubated in 4 mL Hanks' balanced salt solution (HBSS; GIBCO, #C14175500BT) containing 0.05% (w/v) collagenase type IV (Worthington-Biochemical, #LS004186), 0.5% (w/v) dispase II (Worthington-Biochemical, #LS02100), 40 µg/mL DNAse I (TIANGEN, #RT411), and 20 mM 4-(2-hydroxyethyl)-1-piperazineethanesulfonic acid (HEPES) for 30 min at 37 °C. Enzymes were inactivated with 4 mL of Ca^2+^- and Mg^2+^-free HBSS containing 2 mM EDTA and 20 mM HEPES. The digested product was gently passed through a P1000 pipette to obtain a homogeneous cell suspension. The contents of a petri dish containing the digest medium and brain pieces were poured onto a 70 µm strainer, and pieces of the brain were pushed through the filter using the plunger of a sterile 5 mL syringe in a grinding motion until there was no more tissue visible. Next, washed the filter with the wash media and the cell strainer was continuously topped up during this process to wash through any cells trapped in the filter. The cell suspension was centrifuged at 500 × g for 5 min at 4 °C. The obtained cells were resuspended in 30% isotonic Percoll (GE, #17089102) containing 40 µg/mL DNAse I in DMEM and underlaid with 70% isotonic Percoll in HBSS before centrifugation at 600× g for 30 min at 25 °C. Interface cells were collected for incubation with 9 mL HBSS followed by centrifugation at 900 × g for 20 min to remove the density gradient medium. Aspirate supernatant and resuspend with 1mL growth medium and subsequent PBS (Ca^2+^ and Mg^2+^-free) containing 2% FBS and 1 mM EDTA to remove the growth medium. The cells were incubated with CD11b-coupled magnetic beads for 20 min at 4 °C. Subsequently, the cells were washed with sorting buffer (Miltenyi Biotec, #130-091-376-1), then loaded onto MS columns and separated on a MidiMACS Separator. After washing using a sorting buffer three times, the targeted cells were washed using sorting buffer. The purity of the microglia was analyzed with a flow cytometer by staining with CD11b and CD45. This protocol results in a high purification of microglia as reflected by a percentage of CD11b^+^CD45^lo^ cells greater than 85%. Our protocol approximately generates a total number of 2.0×10^5^ to 4.0×10^5^ microglia cells per adult mice brain.

### CRISPR-Cas9-mediated knockout of AhR

To deplete AhR, we designed a single guide RNA (sgRNA) that targeted the exon 2 and positioned them in the sgRNA scaffold within P×459-SpCas9 plasmids. The sgRNA-targeting sequence was 5'- CGAAATCCTGACCTACGTGCAGG-3'. The cell clone was examined by genotyping PCR using the following primers: F1, 5'-GTTGCTGTTGCTCTAGTTGCAG-3' coupled with R1, 5'- GATATCAGAAGCATGCAGAACG-3'.

### RNA Fluorescence *in situ* hybridization and immunofluorescence microscopy

RNA-FISH was performed according to the protocol provided in the RNA FISH Kit (GenePharma, #F04401). In brief, cells were cultured onto confocal dishes the day before the experiment. After performing the corresponding treatment for each group, the samples were washed three times with PBS for 3 min followed by fixing with 4% PFA for 15 min at room temperature (RT) before permeabilization with 100 μL precooled 0.1% Buffer A for 5 min. After washing twice with 100 μL PBS for 5 min each time, 100 μL 2× Buffer C was added to each sample and incubated in a 37 °C cell culture incubator for 30 min. Buffer C was removed and each sample was incubated successively with 100 μL 70%, 85% ethanol, and 100% ethanol for 3 min at RT. After drying at RT, 100 μL diluted 3'-CY3-conjugated linc-AhRA probes (50 μg/mL) in 1× Buffer E (Buffer E was preincubated at 73℃ for 30 min) was added to each sample. Next, the incubated samples were denatured at 73 °C for 5 min and then placed in a 37℃ incubator overnight. After washing with 100 μL 0.1% Buffer F, 2× Buffer C, and 1× Buffer C for 5 min, the cell nucleus was labelled with 4′,6-diamidino-2-phenylindole (DAPI, included in the kit) at RT protected from light. After washing twice with PBS for 5 min each time, the samples were observed under a Zeiss LSM510 Meta confocal system equipped with a 63× oil-immersion objective lens (Carl Zeiss, Oberkochen, Germany). The fluorescence intensity was quantified using Image J software. The probe sequence was CC^+^TAAAACCAGCGGA^+^TATCT (5'-3'). The “+” label indicates an LNA modification at the subsequent base.

### Western Blotting

Western blotting for this study was similar to the procedure in our previous study 60. Briefly, the cultured cells with the indicated treatment were harvested and lysed in cell lysis buffer (Beyotime, #P0013B) containing a protease and phosphatase inhibitor cocktail (Beyotime, #P1045). The lysates were normalized to equal amounts of protein using the bicinchoninic acid (BCA) Protein Assay Kit (Beyotime, #P0011). The proteins were separated by sodium dodecyl sulfate polyacrylamide gel electrophoresis (SDS-PAGE, 8%-12% acrylamide) then transferred to polyvinylidene difluoride membranes (Millipore, #ISEQ00010) followed by blocking with 5% skimmed milk. The membranes were incubated with primary antibodies overnight, followed by incubation with anti-mouse (1:6000 dilution), anti-rabbit (1:8000 dilution), or anti-goat (1:5000 dilution) horseradish peroxidase (HRP)-conjugated secondary antibodies. The blots were visualized with enhanced chemiluminescence (ThermoFisher Scientific, #34580) and imaged with a Tanon 5200 image analysis system (Tanon, Shanghai, China). The protein bands were quantified with the ImageJ software (Bio-Rad).

### Northern Blotting

The biotin-labeled single-stranded RNA probe for linc-AhRA was designed and synthesized by SaiCheng Biotechnology Company (Guangzhou). Subsequent hybridization was performed using the NorthernMax kit (Thermo Fisher Scientific) following the manufacturer's protocol. The probe sequence for linc-AhRA was CGGAUAUCUGUCUUGAUGGUUUCAAGGGAGGCAUCGCACCCCAGGCUCACUGCCUACGUGAUAGCAGAAUCUAAG.

### tRSA RNA Pull-down Assay, silver staining, mass spectrometry analysis, and western blotting identification

Given that the addition of the tRNA scaffold to tRSA captures RNA-interacting proteins more efficiently than the biotinylated transcripts, the tRSA RNA pull-down assay was performed in this study in accordance with prior studies with minor modifications 29, 68. The reagents for this experiment were obtained from the Pierce Magnetic RNA-Protein Pull-Down Kit (Pierce, #20164) unless otherwise stated. Briefly, linc-AhRA was cloned into a pcDNA3.1(+) plasmid with the tRSA tag at the 5' end. The plasmids were linearized as a template for the* in vitro* transcription of tRSA or tRSA-linc-AhRA using the Takara *in vitro* Transcription T7 Kit (Takara, #6140). The RNA product was digested using DNase (TIANGEN, #RT411) purified using the RNAclean Kit (TIANGEN, #DP412). Ten micrograms of purified RNAs per reaction was denatured for 5 min at 65℃ in RNA structure buffer (10 mM HEPES, 10 mM MgCl_2_, pH 7.0) and slowly cooled to RT. Afterwards, the fold RNAs were incubated with 50 μL of Streptavidin Dynabeads for 20 min at RT in the presence of RVC (10 mM) (NEB, #S1402S). BV2 cells (2.0×10^6^) were harvested in 300 μL lysis buffer (Beyotime, #P0013) with 1 mM phenylmethylsulfonyl fluoride (PMSF; Beyotime, #ST505) and protease inhibitor (Beyotime, #P1008) by sonicating five times for 10 s with an interval of 1 min on ice and then centrifuged at 12,000 g for 10 min at 4 °C. The supernatant was pre-cleared by incubation with 50 μL Streptavidin Dynabeads for 20 min at 4 °C. Afterwards, the pre-cleared lysate was incubated with the folded RNAs for 2 h at 4 °C in Protein-RNA Binding Buffer. After washing four times (5 mins each) with Wash Buffer containing 10 mM RVC, 40 μL Elution Buffer was added to the magnetic beads and incubated for 30 min at 37 °C to obtain pull-down enriched proteins. Subsequently, added SDS loading buffer to the enriched proteins followed by separation with SDS-PAGE gel (Beyotime, #P0012A). Finally, the gel was stained using the Fast Silver Stain Kit (Beyotime, #P0017S) according to the manufacturer's instructions. The specific bands in tRSA-*linc-AhRA* compared to those in tRSA were cut, and the targeted protein was identified using MS. The pull-down enriched proteins were confirmed by western blotting. The primers for constructing plasmids for the RNA pull-down assay are presented in **Table S13**.

### RNA immunoprecipitation assay

The RNA immunoprecipitation assay was performed using the Magna RIP RNA-Binding Protein Immunoprecipitation Kit (Millipore, #17-701) following the manufacturer's instructions with minor optimizations. In brief, 115 μL RIP Lysis Buffer containing RNase inhibitor and protease inhibitor was added to each 15 cm plate with 2.0 × 10^7^ BV2 cells and incubated on ice for 5 min to increase the size of the cells. The cell lysates were collected and frozen once at -80 °C. Then, 50 μL of magnetic beads was mixed with 5 μg primary antibody or negative control IgG and incubated for 30 min at RT with rotation. Subsequently, the lysates were quickly thawed and centrifuged at 14,000 g for 10 min at 4 °C. Afterwards, 100 μL of the supernatant was transferred to antibody-coupled beads and incubated overnight at 4 °C with rotation. In addition, 10 µL of the RIP lysate supernatant was removed, placed in a new tube, and marked as “10% input”. Next, the beads were washed with RIP Wash Buffer six times, and the samples were digested with proteinase K at 55 °C for 30 min. Finally, the RNA was purified with phenol: chloroform: isoamyl alcohol (125:24:1, pH = 4.3) (Aladdin, #P120621) and the enrichment of specific RNAs was analyzed with the One Step TB Green® PrimeScript™ RT-PCR Kit II (TAKAR, #RR086A). The amount of immunoprecipitated RNAs was represented as the fold change of the amount of IgG enriched RNA. All the materials were included in the RIP Kit unless otherwise stated.

### Cleavage under targets and tagmentation (CUT&Tag)

Given that only a small sample of primary microglia was acutely isolated from the mice brain, we performed the enzyme-tethering strategy known as CUT&Tag 69 to confirm that AhR binds to the promoter of linc-AhRA *in vivo* following the instructions for the Novo CUT&Tag High-Sensitivity Kit (Novoprotein, #N259-YH0) with minor optimizations. Briefly, cells were harvested, counted, and centrifuged for 3 min at 600 × g at RT. Next, cells were washed twice in 1.2 mL Wash Buffer containing a protease inhibitor cocktail by gentle pipetting and collected by minor centrifugation at 300 × g for 3 min. Of note, removed the supernatant, and the cells were resuspended in 800 µL pre-cooled antibody buffer containing 1.5 μg primary antibody. Primary antibody incubation was performed on a rotating platform for 2 h at RT. A corresponding secondary antibody was diluted 1:100 in 100 µL of Dig-Wash buffer and incubated with the cells at RT for 30 min to increase the number of Protein A binding sites for each bound antibody. Then, we prepared a 1:250 dilution of pA-Tn5 adapter complex in Dig-300 Buffer. After removing the supernatant by centrifugation at 300 × g for 3 min, 100 µL was added to the cells with gentle vortexing and incubated with pA-Tn5 at RT for 1 h. The cells were washed three times for 10 mins upside down in 0.8 mL Dig-med Buffer to remove unbound pA-Tn5 protein. Next, the cells were resuspended in 300 µL tagmentation buffer and incubated at 37 °C for 1 h. For an interruption of tagmentation, 10 µL of 0.5 M EDTA, 3 µL of 10% SDS, and 2.5 µL of 20 mg/mL Proteinase K was added to 300 µL of the sample, which was incubated at 50 °C for 1 h to deactivate Proteinase K. Afterwards, added 300 µL Phenol-Chloroform-Isoamyl Alcohol (25:24:1) (BioFlux, #BSA03M1) to each tube followed by vortexing. The sample was transferred to a phase-lock tube for centrifugation at 16000 × g for 3 min. Next, 300 µL chloroform was added to each sample, inverted 10 times, and centrifuged at 16000 × g for 3 min at RT. The aqueous phase from each tube was separately pipetted into a 1.5 ml tube containing 750 μl of 100% ethanol and mixed well with a pipette tip. After cooling on ice, the samples were centrifuged at 16000 x g for 15 min at 4℃. Removed the supernatant and 1 mL 100% ethanol was used to wash each sample by centrifuging at 16000 × g for 1 min. After drying, added 30 µL TE-RA buffer to each sample to resolve the DNA. The final products were subjected to qPCR analysis. The primers for CUT&Tag detection are provided in **Table S14**.

### Immunoprecipitation

Immunoprecipitation assays were performed following our previous studies with optimization 60. In brief, cells were harvested then lysed using IP lysis buffer (Beyotime, # P0013) containing 1 mM PMSF and a proteasome inhibitor cocktail (Beyotime, #P1008). Pre-cleared cell lysis was performed by incubation with agarose-coupled IgG antibodies to remove non-specific binding factors. Pre-cleared lysates were added to primary antibodies or antibody-coupled agarose and incubated with rotation overnight at 4 °C. The sample was centrifuged for 30 seconds at 4 °C, and the pellet was washed three times using pre-cold lysis buffer containing 1 mM PMSF. The washed sample was centrifuged at 500 g and 4 °C for 30 sec. The last pellet was centrifuged at 500 g and 4 °C for 2 min. After aspirating the supernatant, the pellet was resuspended in 50 μL 1×SDS loading buffer and vortexed. The sample was heated to 100 °C for 5 min and micro centrifuged for 1 min at 14,000 × g. The supernatant was then subjected to immunoblotting analysis. To reduce the appearance of denatured IgG heavy chains on the indicated bands, secondary antibodies including IPkine HRP AffiniPure Goat Anti-Mouse IgG Light Chain (Abbkine, #A25022) and IPkine HRP AffiniPure Mouse Anti-Rabbit IgG Light Chain (Abbkine, #A25012) were used according to the manufacturer's instructions.

### Immunofluorescence assay

The immunofluorescence assay was performed following our previous study with minor modifications 60. In brief, cells were harvested following the corresponding treatment and washed with PBS. Afterwards, the cells were fixed with 4% PFA, followed by washing with PBS. Next, the samples were permeabilized with 0.1% NP-40 for 4 min and washed with PBS. After being blocked with 5% bovine serum albumin (BSA) for 90 min, the samples were washed and subsequently stained with primary antibody overnight at 4 °C followed by incubation with the Alexa Fluor conjugated secondary antibody (Life Technologies) at RT. The nuclei were labelled using DAPI (Beyotime, #C1005). Fluorescence images were captured using a Zeiss LSM510 Meta confocal system (Carl Zeiss, Oberkochen, Germany) to visualize the co-localization of TRIM27 and TBK1. To analyze the replication of the EGFP reporter virus, we harvested the samples and observed them using the NIS-Elements Viewer (Nikon, Japan) and quantified them using Image J software.

### Antisense LNA long RNA GapmeRs design and application

Antisense LNA GapmeR targeting linc-AhRA was produced according to a custom design generated by a proprietary design software for optimal performance 70, 71 (https://geneglobe.qiagen.com/us/customize/rna-silencing/antisense-lna-gapmers). All potential LNA GapmeRs were ranked according to their design score, with 1 being the best score. The GapmeRs with a score in the top 3 and negative control LNA were selected for synthesis by QIAGEN. 5' and 3' modifications were indicated within the product sequence (**Table S15**). The antisense LNA GapmeRs contains phosphonothioate backbone modifications indicated by “*” in the product sequence, and the position of the LNA modification is not shown. The jetPEI transfection reagent (Polyplus, #PT-114-15) was used to obtain LNA oligonucleotides (60 pM), and the cells were maintained in the medium used for transfection for 24 h. Afterwards, the transfection medium was replaced with a fresh medium to maximize cell viability. After transfecting for 2 h, the knockdown efficiency of the LNA GapmeRs against linc-AhRA was assessed using qRT-PCR. The LNAs with high efficiency after knocking down linc-AhRA were selected for subsequent experiments.

### Mice generation

Our mice model was generated using a CRISPR-Cas9 system with a C57BL/6J mouse background by the Shanghai Model Organisms Center. Briefly, a CAG promoter and a promoter-less linc-AhRA gene were introduced into the well-defined Rosa26 locus by homology recombination. The donor vector containing a 3.3 kb 5′ homology arm, a linc-AhRA gene expression cassette with floxed-STOP-floxed (CAG-LSL-linc-AhRA-WPRE-polyA), and a 3.3 kb 3′ homology arm was cloned. Cas9 mRNA was transcribed *in vitro* with an mMESSAGE mMACHINE T7 Ultra Kit (Ambion) and subsequently purified using a MEGAclear Kit (Thermo Fisher). 5′-GGGGACACACTAAGGGAGCT-3′ was chosen as the single guide RNA targeting Rosa26, *in vitro* transcribed using a MEGAshortscript Kit (Thermo Fisher), and purified using a MEGAclear Kit. A donor vector with guide RNA and Cas9 mRNA was microinjected into C57BL/6J fertilized eggs. Mice (F0 generation) positive for homologous recombination were identified using long PCR. The genomic DNA for genotyping (FOREGENE, #TP-01341) was prepared from mouse tails. The primers used for genotyping are shown in **Table S16 (linc-AhRA)** and **Table S17 (Cre)**. The F0 mice were crossed with C57BL/6J mice to obtain heterozygous Cre-dependent linc-AhRA KI mice (F1 generation). Positive (**#**3, 4, 5, 6, 7) mice (F1 generation) were again identified with long PCR. We crossed Cre-dependent linc-AhRA KI mice with *Cx3cr1*^creERT2^ mice to obtain TAM-induced microglia-specific linc-AhRA KI (Cx3cr1^creERT2^:Rosa26-LSL-*linc-AhRA*) mice.

### Mice model of HSV-1 brain infection

We used microglia linc-AhRA KI mice to assess the role of microglial linc-AhRA in the CNS innate antiviral response. To initiate the expression of linc-AhRA, 6-7-week-old male mice were injected subcutaneously with 4 mg TAM in 200 µl warm corn oil at two time points 48 h apart. Homozygous Cre-dependent microglial linc-AhRA KI male mice were Cx3cr1^creERT2^-negative (Cx3cr1*^creERT2^*-negative Rosa26-LSL-*linc-AhRA*) were used as littermate controls. The mice were randomly allocated into different experimental groups and the investigators were blinded to mice allocation during the experiments. The microglia-specific linc-AhRA KI and control mice were intranasally inoculated with HSV-1 (1×10^7^ PFU/mouse) and the weights of all mice were recorded daily along with their HSE symptoms. The scoring rules for HSE symptoms were based on previous studies 2, 72 with minor modifications: hair loss (0: none, 1: minimal periocular hair loss, 2: moderate periocular hair loss, 3: severe hair loss limited to the periocular region, 4: severe and extensive hair loss); hydrocephalus (0: none, 1: minor bump, 2: moderate bump, 3: large bump); symptoms related to neurological disease (0: normal, 1: jumpy, 2: uncoordinated, 3: hunched/lethargic, 4: unresponsive/no movement); eye swelling/lesions (0: none, 1: one eye with minor swelling, 2: one eye with moderate swelling, 3: one eye with severe swelling and skin lesions, 4: two eyes with swelling). The mice were sacrificed for histological analysis and virus quantification when a body-weight reduction of 30% occurred. For histological analysis, mice were perfused with cold PBS and in succession with 4% PFA. Subsequently, the whole brain was dissected, fixed in 4% PFA solution, embedded in paraffin, sectioned, stained with hematoxylin-eosin solution or anti-HSV-1 antibody and examined under light microscopy. The levels of TBK-1 in the microglia were analyzed by IBA-1 and TBK1 staining in the BS section followed by observation using a fluorescence microscope to determine the ratio of IBA-1^+^TBK-1^+^ positive cells. For virus quantification, tissues were dissected and prepared in a 10% DMEM-based solution. After these samples were repeatedly frozen three times, the HSV-1 abundance was determined using plaque formation assays and qPCR-based HSV-1 genomic DNA copy numbers. To determine the effect of microglial linc-AhRA on the innate antiviral response in the CNS, the tissue RNAs were extracted using TRIzol reagent. The levels of I-IFN-associated genes were analyzed. Protein samples of the BS were also prepared in RIPA buffer to analyze I-IFN signaling activation in the mice brain. We also investigated the effect of linc-AhRA on the innate antiviral response of microglia upon HSV-1 infection at the early stage. The microglia in the BS of all groups at 2 d.p.i. were sorted using anti-CD11b^+^ antibody-coupled microbeads following prior studies with minor modifications 61, 66, 67. Total RNA from microglia was extracted using the RNAprep Pure Micro Kit (TIANGEN, #DP420). The mRNA expression of the corresponding innate antiviral genes was determined using qRT-PCR. Kept all mice under a 12-h day and light cycle. All mice experiments were performed under the guidelines for Laboratory Animal Science at Jinan University. No data were excluded.

### Statistical analysis

Detailed information regarding the statistical analysis for the data are described in corresponding figure legends. We used GraphPad Prism version 8.0 to perform statistical analyses. P values less than 0.05 were considered statistically significant. n.s, not significant, * P < 0.05, ** P < 0.01.

## Supplementary Material

Supplementary figures and tables.
