# Supplementary material for: A novel lncRNA linc-AhRA negatively regulates innate antiviral response in murine microglia upon neurotropic herpesvirus infection
Source: Theranostics. 2021 Sep 21;11(19):9623–51. doi: 10.7150/thno.64880 (PMC8490526; doi:10.7150/thno.64880)
Supplement: Supplementary file 1 — Supplementary figures and tables. [file thnov11p9623s1.pdf]

# Supplementary materials for

**A novel lncRNA linc-AhRA negatively regulates innate antiviral response in murine microglia upon neurotropic herpesvirus infection**

Yiliang Wang[1, 2, 3, 4], Weisheng Luo[1, 2, 3, 4], Lianzhou Huang[1, 2, 3, 4], Ji Xiao[1, 2, 3, 4], Xiaowei Song[1, 2, 3, 4], Feng Li[1, 2, 3, 4], Yuying Ma[1, 2, 3, 4], Xiaohui Wang[1, 2, 3, 4], Fujun Jin[1, 2, 3, 4], Ping Liu[1, 2, 3, 4], Yexuan Zhu[1, 2, 3, 4], Kaio Kitazato[5, *], Yifei Wang[1, 2, 3, 4, *], Zhe Ren[1, 2, 3, 4, *]

Correspondence to: **kkholi@nagasaki-u.ac.jp** (Kaio Kitazato); **rz62@163.com** (Zhe Ren); **twang-yf@163.com** (Yifei Wang)

## Supplemental Figure legends

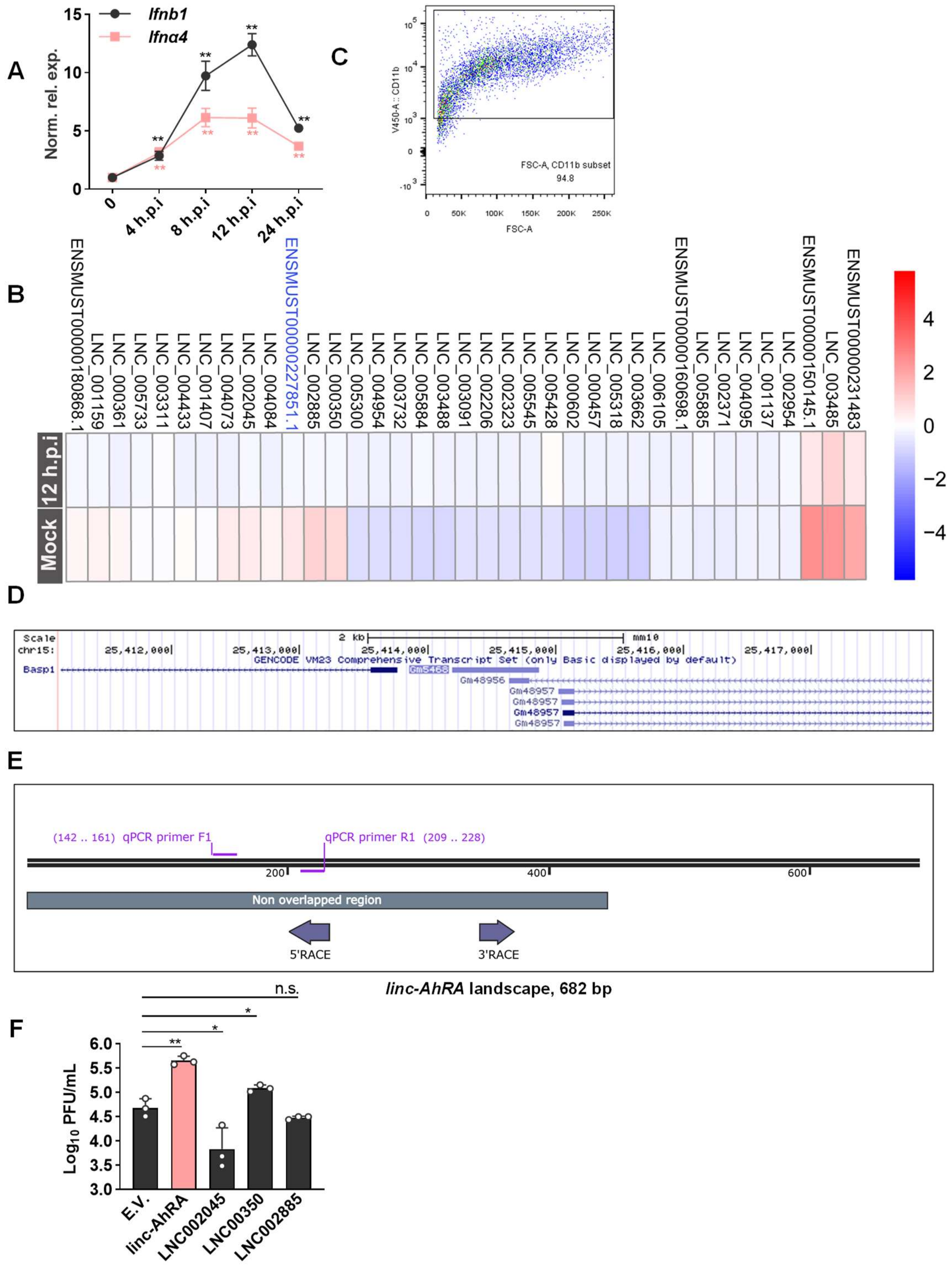

**Figure S1. A,** qPCR analysis of the expression of I-IFNs in primary microglia with HSV-1 infection (MOI 1) for the indicated hours. **B,** The cluster heat map shows upregulated DELs with expression fold change > 16 from lncRNA-sequencing data. The colors represent transcripts above (red) or below (blue) the global median scaled to the corresponding fold activation or repression respectively as shown in the scale bar. **C,** Flow cytometry analysis of microglia digested from mixed glial cultures. The ratio of the $CD11b^{+}$ subset was presented. **D,** The genomic architecture of linc-AhRA

(GM5468) and its neighbor genes. **E,** Primers used in RACE assay (labeled with arrows) and qPCR analysis (labeled with purple stub) for linc-AhRA. The non-overlapped region with GM48956 was also labeled. **F,** Determination of HSV-1 titers in culture medium supernatants of BV2 cells transfected with plasmids expressing the indicated lncRNAs for 48 h followed by HSV-1 infection (MOI 1) for 12 h using a plaque formation assay. Data are representative of three independent experiments with n = 2 technical replicates (**A**) or n = 3 technical replicates (**F**), three independent experiments (**C**), or the average of three technical replicates as a mixture (**B**), each symbol represents a technical replicate (**F**) (shown as mean and s.d. in **A, F**), One-way ANOVA (and nonparametric) (**A**), Cuffdiff was used for differential expression analysis (**B**), two-tailed unpaired Student's t-test (**F**).

**A**

| Name | Fickett Score | pI | Label |
|---|---|---|---|
| *linc-AhRA* | 0.33538 | 10.85 | noncoding |
| *Neat1* | 0.27682 | 7.98 | noncoding |

**C**

| Label | Start | Stop | Length (nt \| aa) |
|---|---|---|---|
| ORF1 | 175 | 642 | 468\|155 |
| ORF4 | 258 | 470 | 213\|70 |
| ORF3 | 63 | 134 | 72\|23 |

**B**

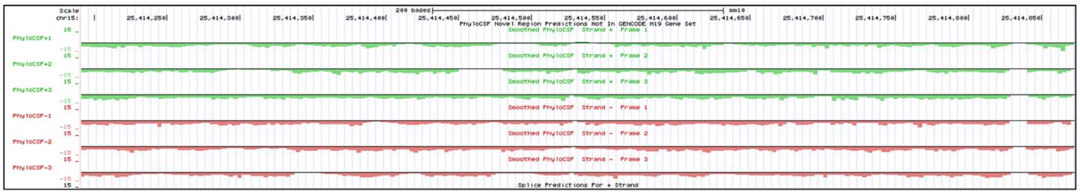

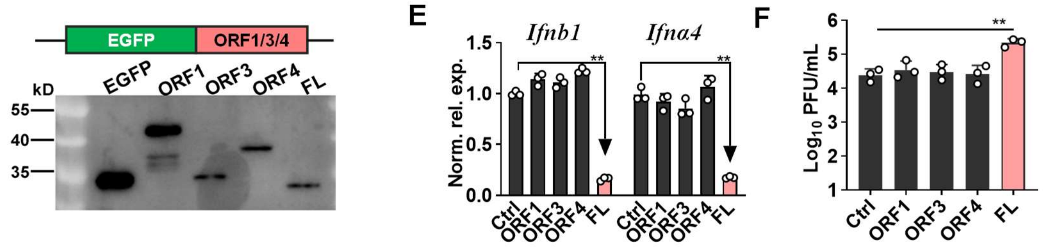


**Figure S2. A**, Coding Potential Calculator analysis of linc-AhRA and Neat1 presented according to the Fickett Score and pI, as well as the coding properties**.** Neat1 was present as a positive control non-coding RNA. **B,** Protein coding potential analysis of *linc-AhRA* using PhyloCSF. **C,** ORFs with a length longer than 20 amino acids within linc-AhRA. **D, Top**: Scheme diagram for constructing plasmids expressing EGFP-ORF. **Bottom**: Immunoblotting results for the indicated ORF-EGFP production in HEK 293T cells. **E,** qPCR analysis of the indicated RNA level following a 6 h infection with HSV-1 in BV2 cells in the presence of the indicated small peptides (10 μg/mL) or plasmids expressing linc-AhRA with an EGFP indicator. **F,** Determination of HSV-1 titers in culture medium supernatants of BV2 cells with a 12 h HSV-1 infection following a treatment of the indicated small peptides (10 μg/mL) for the first 6 h or a transfection of plasmids expressing linc-AhRA with an EGFP indicator. Data are representative of three independent experiments (**D, bottom**), three independent experiments with n = 3 technical replicates (**E-F**), each symbol represents an individual technical replicate (**E-F**) (shown as mean and s.d. in **E-F**), two-tailed unpaired Student's t-test (**E-F**).

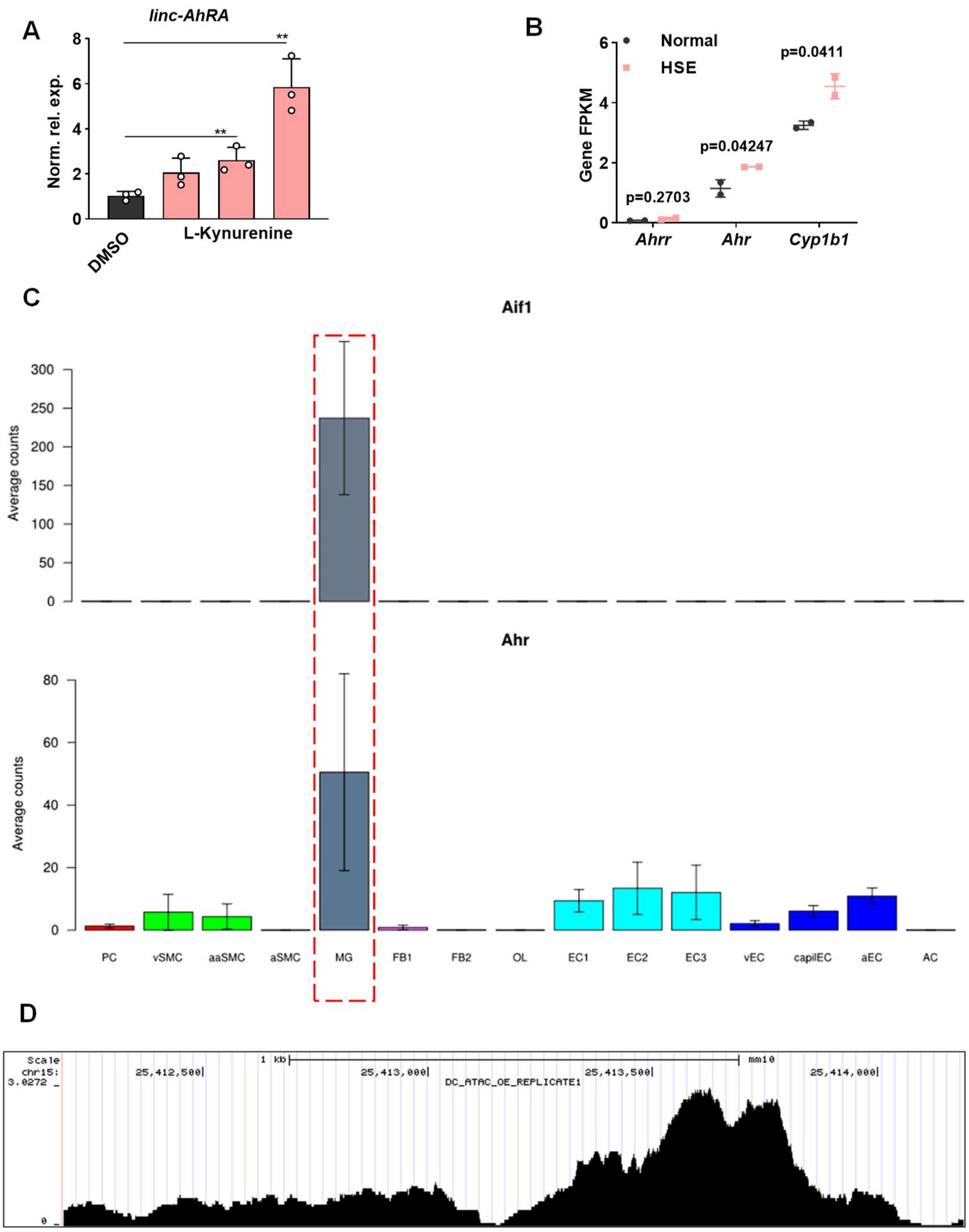


**Figure S3. A,** qPCR analysis of linc-AhRA expression in BV2 cells with a stimulation of L-Kynurenine (100, 200, and 400 μM) for 6 h. Data are normalized relative to DMSO-treated BV2 cells. **B,** The level of AhR-activated genes reflected by the average FPKM value in RNA-sequencing results for olfactory bulb isolated from mice with an infection of $2.0\times10^6$ PFU for 9 days, n = 2 mice per group. **C,** The level of AhR in mouse microglia using the published single-cell RNA-sequencing data for the main CNS cell population (http://betsholtzlab.org/VascularSingleCells/database.html). Abbreviations: PC, Pericytes; SMC, Smooth muscle cells; MG, Microglia; FB, Vascular fibroblast-like cells; OL, Oligodendrocytes; EC, Endothelial cells; AC, Astrocytes; v, venous; capil, capillary; a, arterial; aa, arteriolar; 1,2,3, subtypes. **D,** Binding landscape of AhR within linc-AhRA promoter in published ChIP data obtained from mouse macrophages with LPS (100 ng/mL) stimulation for 2

h using Cistrome Data Browser analysis (http://cistrome.org/db/#/). Data are representative of three independent experiments with n = 3 technical replicates (**A**) or the average of one experiment with n = 2 mice (**B**), each symbol represents an individual technical replicate or one mouse (**A-B**) (shown as mean and s.d. in **A-B**), One-way ANOVA (and nonparametric) (**A**), Cuffdiff differential expression analysis (**B**).

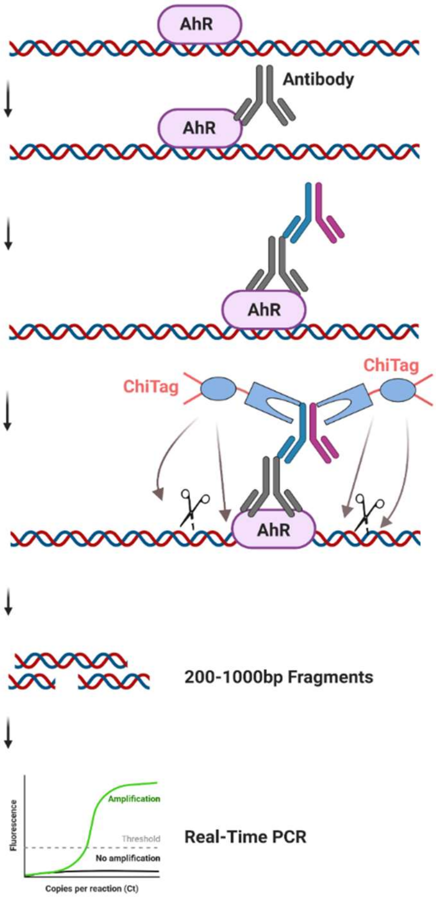


**Figure S4.** Workflow for detecting the enrichment of AhR within linc-AhRA promoter using AhR CUT&Tag assay.

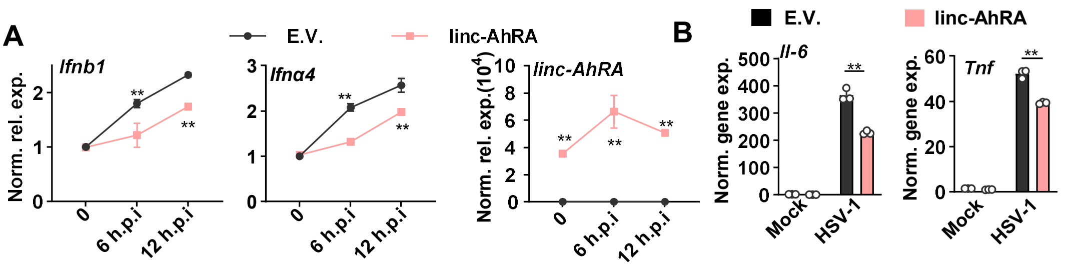


C

| Gene | FPKM | |
|---|---|---|
| | Tg-Ctrl | Tg-linc-AhRA |
| *Ifnb1(**)* | 29.37 | 16.79 |
| *Isg15 (**)* | 822.63 | 662.67 |
| *Cxcl10 (**)* | 575.13 | 378.35 |
| *Isg20 (**)* | 109.38 | 105.08 |
| *Ifna4 (*)* | 0.13 | 0 |

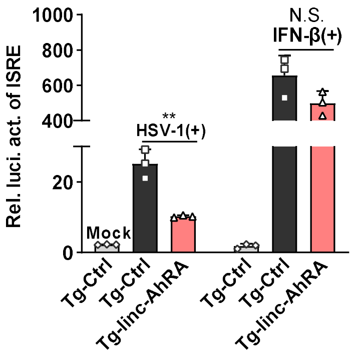


**Figure S5. A,** qPCR analysis of the expression of the indicated genes in primary microglia with the transfection of linc-AhRA-expressing and E.V. plasmids for 48 h and subsequent HSV-1 infection (MOI 1) for the indicated hours. **B,** qPCR analysis of the expression of the inflammatory factors in BV2 cells transfected with linc-AhRA-expressing and E.V. plasmids, followed 48 h later by HSV-1 infection (MOI 1) for 12 h. **C,** Determination of the average FPKM of I-IFNs and ISGs in BV2 cells stably expressing linc-AhRA with HSV-1 infection (MOI 1) for 6 h using RNA-sequencing. **D,** Dual luciferase analysis of ISRE activity in linc-AhRA-expressing stable BV2 cells 24 h after co-transfecting a firefly luciferase reporter (ISRE-Luc) and TK-renilla plasmids, followed by HSV-1 (MOI 1) or stimulation with IFN-β (100 pg/mL) for another 12 h. Data are three independent experiments with n = 3 technical replicates (**A-B, D**), or the average of three technical replicates as a mixture (**C**), each symbol represents an individual technical replicate (**B, D**) (shown as mean and s.d. in **A-B, D**), two-way ANOVA (**A**), two-tailed unpaired Student's t-test (**B, D**), Cuffdiff was used for differential expression analysis (**C**).

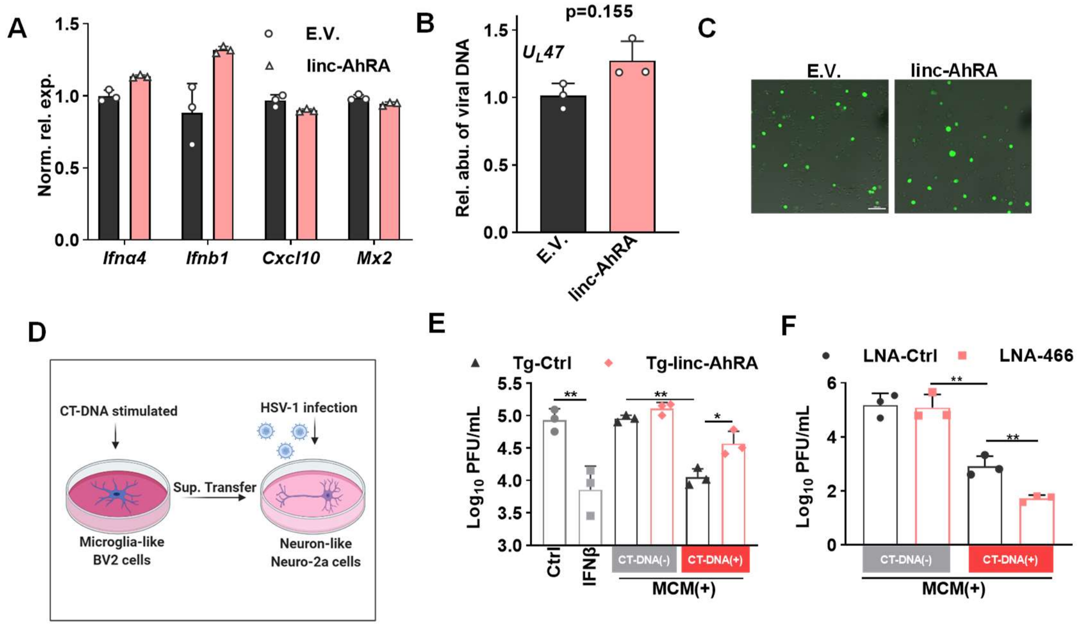


**Figure S6. A,** qPCR analysis of the expression of the indicated genes in Neuro-2a cells with the transfection of linc-AhRA-expressing and E.V. plasmids for 48 h and subsequent HSV-1 infection (MOI 1) for 6 h. **B,** Determination of viral DNA abundance in Neuro-2a cells with the transfection of linc-AhRA-expressing and E.V. plasmids for 48 h and subsequent HSV-1 infection (MOI 1) for 24 h. **C,** Fluorescence microscopy images of viral replication (green) in Neuro-2a cells with EGFP-HSV-1 infection (MOI 1) for 24 h, following the transfection of plasmids expressing linc-AhRA for 48 h, scale bar, 100 µm. **D,** Schematic illustration of a cellular model used to study intercellular communication between microglia and neuron. Supernatant from microglia stably expressing linc-AhRA (**E**) or microglia with linc-AhRA knockdown (**F**) treated for 24 h with jetPRIME buffer or CT-DNA (1µg/mL) was transferred to neuron-like Neuron-2a cells, followed by HSV-1 infection (MOI 1) for 36 h. Supernatants were harvested and the HSV-1 titers was measured using a plaque formation assay. The medium and IFN-β (100 pg/mL) were used as the negative and positive controls, respectively. MCM, microglia conditioned medium. Data are representative of three independent experiments with n = 3 technical replicates (**A-B, E-F**), three independent experiments (**C**), each symbol represents an individual technical replicate (**A-B, E-F**) (shown as mean and s.d. in **A-B, E-F**), two-tailed unpaired Student's t-test (**A-B, E-F**).

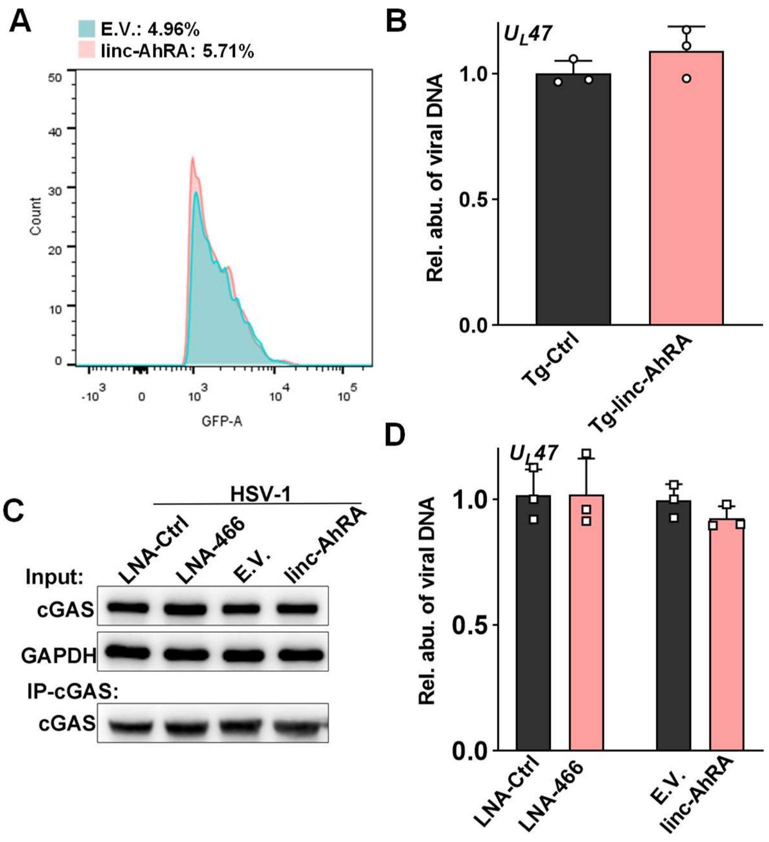


**Figure S7. A,** Flow cytometry analysis of EGFP in BV2 cell with EGFP-HSV-1 (MOI 3) infection for 90 min in the context of linc-AhRA overexpression after removing the free viral particle. **B,** qPCR-based determination of the amount of viral DNA in BV2 cells stably expressing linc-AhRA with HSV-1 infection (MOI 3) for 90 min and subsequent removal of the free virus. **C,** BV-2 cells with indicated expression of linc-AhRA were infected with HSV-1(MOI 3) for 2 h. Lysates were immunoprecipitated with anti-cGAS and the eluate was analyzed by immunoblotting to determine the efficiency of the precipitation (**C**) or qPCR to detect viral DNA after isolating DNA (**D**) (data are shown as relative abundance). Data are representative of three independent experiments (**A, C**), three independent experiments with n = 3 technical replicates (**B, D**), each symbol represents an individual technical replicate (**B, D**) (shown as mean and s.d. in **B, D**), two-tailed unpaired student's t-test (**B, D**).

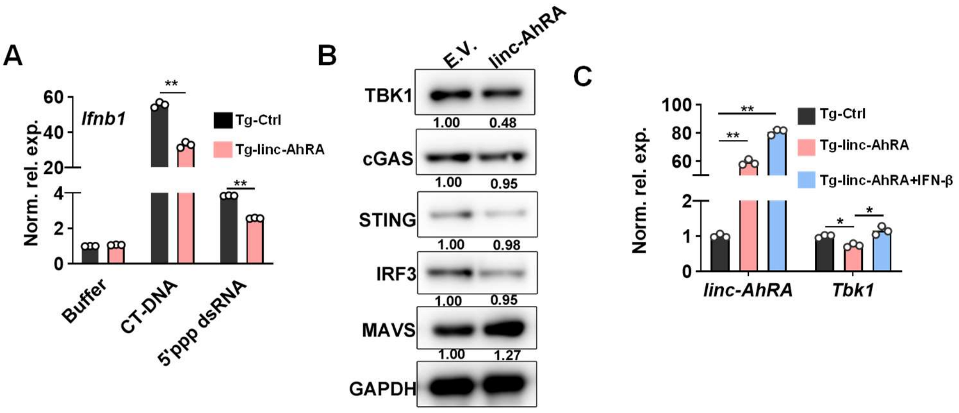


**Figure S8. A,** qPCR analysis of the expression of the indicated genes in BV2 cells stably expressing

linc-AhRA, followed by the transfection of CT-DNA (1 μg/mL) or 5'ppp-dsRNA (1 μg/mL) for 6 h. Data are normalized relative to buffer-stimulated control BV2 cells. **B,** Immunoblot analysis of the indicated factors in primary microglia following the transfection of linc-AhRA expression plasmids for 48 h. **C,** qPCR analysis of the expression of the indicated genes in BV2 cells stably expressing linc-AhRA, followed by IFN-β stimulation (100 pg/mL) for 6 hours. Data are representative of three independent experiments with n = 3 technical replicates (**A, C**), three independent experiments (**B**), each symbol represents an individual technical replicate (**A, C**) (shown as mean and s.d. in **A, C**), two-tailed unpaired Student's t-test (**A, C**).

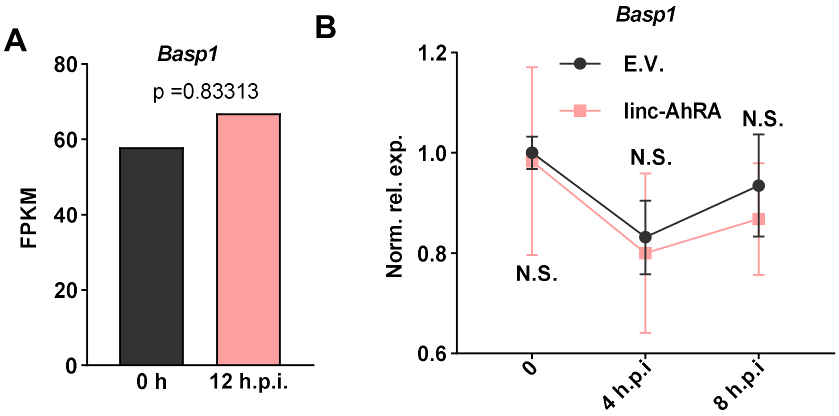


**Figure S9. A,** The average FPKM of Basp1 determined by analyzing the RNA-sequencing results for samples as illustrated in **Figure 1** (**A**). **B,** qPCR analysis of *Basp1* expression in BV2 cells with the transfection of linc-AhRA-expressing and E.V. plasmids for 48 h and subsequent HSV-1 infection (MOI 1) for the indicated hours. Data are representative of three independent experiments with n = 3 technical replicates (**A**) (shown as mean and s.d. in **B**). Data are the average of three technical replicates as a mixture (**A**). Cuffdiff was used for differential expression analysis (**A**), two-way ANOVA (**B**).

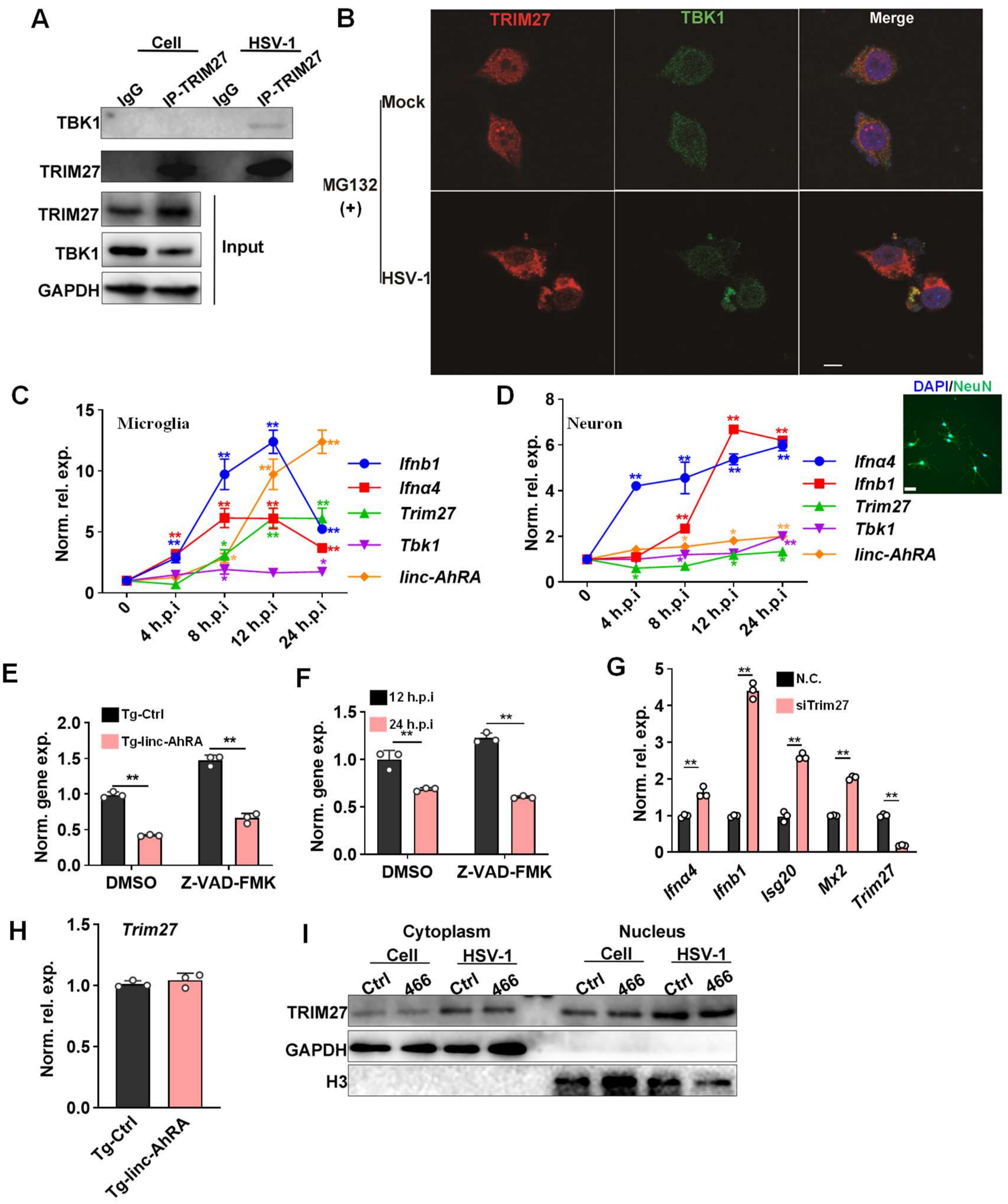


**Figure S10. A,** Co-immunoprecipitation (Co-IP) analysis of the TBK1-TRIM27 interaction in BV2 cells with HSV-1 infection (MOI 1) for 24 h. **B,** Confocal microscope-based fluorescence analysis of the co-localization of endogenous TBK1 (green) and TRIM27 (red) in BV2 cells with HSV-1 infection (MOI 1) for 18 h and subsequent treatment with MG-132 (10 μM) for 6 h. Nuclei were labeled with DAPI (blue). Scale bars, 10 μm. **c,** qPCR analysis of the expression of indicated genes in primary microglia with HSV-1 infection (MOI 1) for the indicated hours as in **Figure S1A**. **D,** qPCR analysis of the expression of indicated genes in primary neuron with HSV-1 infection (MOI 1) for the indicated hours. **E,** qPCR analysis of the expression of *Ifnb1* following a 24 h infection with HSV-1 (MOI 1) in BV2 cells stably expressing linc-AhRA with an incubation of Z-VAD-FMK (50 μM) for final 6 h. **F,** qPCR analysis of the expression of *Ifnb1* following a indicated hours infection with HSV-1 (MOI 1) in BV2 cells with an incubation of Z-VAD-FMK (50 μM) for final 6 h. **G,** qPCR analysis of the expression of the indicated genes in BV2 cells 6 h after HSV-1 infection

(MOI 1) following the transfection of siRNA targeting *Trim27* for 42 h. **H,** qPCR analysis of *Trim27* expression in BV2 cells stably expressing linc-AhRA. **I,** Immunoblotting analysis of TRIM27 in subcellular-fraction of BV2 cells 24 h after LNA transfection followed by HSV-1 infection (MOI 1) for another 24 h. Data are representative of three independent experiments (**A-B, I**), three independent experiments with n = 2 technical replicates (**C-D**) or with n = 3 technical replicates (**E-H**), each symbol represents an individual technical replicate (**E-H**) (shown as mean and s.d. in **C-H**). One-way ANOVA (and nonparametric analysis) (**C-D**), two-tailed unpaired Student's t-test (**E-H**).

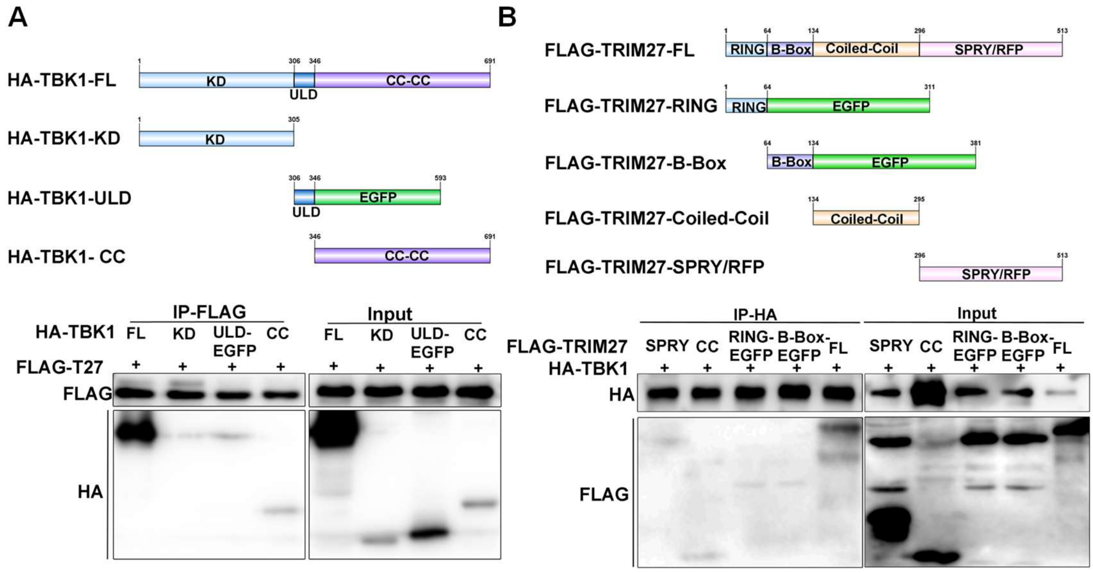


**Figure S11. A, Top:** Schematic illustration of HA-tagged indicated domain of TBK1. FL, full length; KD, kinase domain; ULD, ubiquitin-like domain; EGFP, enhanced Green Fluorescent Protein; CC, coiled-coil domain. Numbers above indicate the positions of amino acids; **bottom:** Co-IP and immunoblot analysis of HEK 293T cells transfected with the indicated domain of TBK1 along with FLAG-TRIM27. **B, Top:** Schematic illustration of FLAG-tagged indicated domain of TRIM27.SPRY, SPla/Ryanodine receptor. Numbers above indicate the positions of amino acids; **bottom:** Co-IP and immunoblot analysis of HEK 293T cells transfected with the indicated domain of FLAG-TRIM27 along with HA-TBK1. Data are representative of three independent experiments (**A-B[bottom]**).

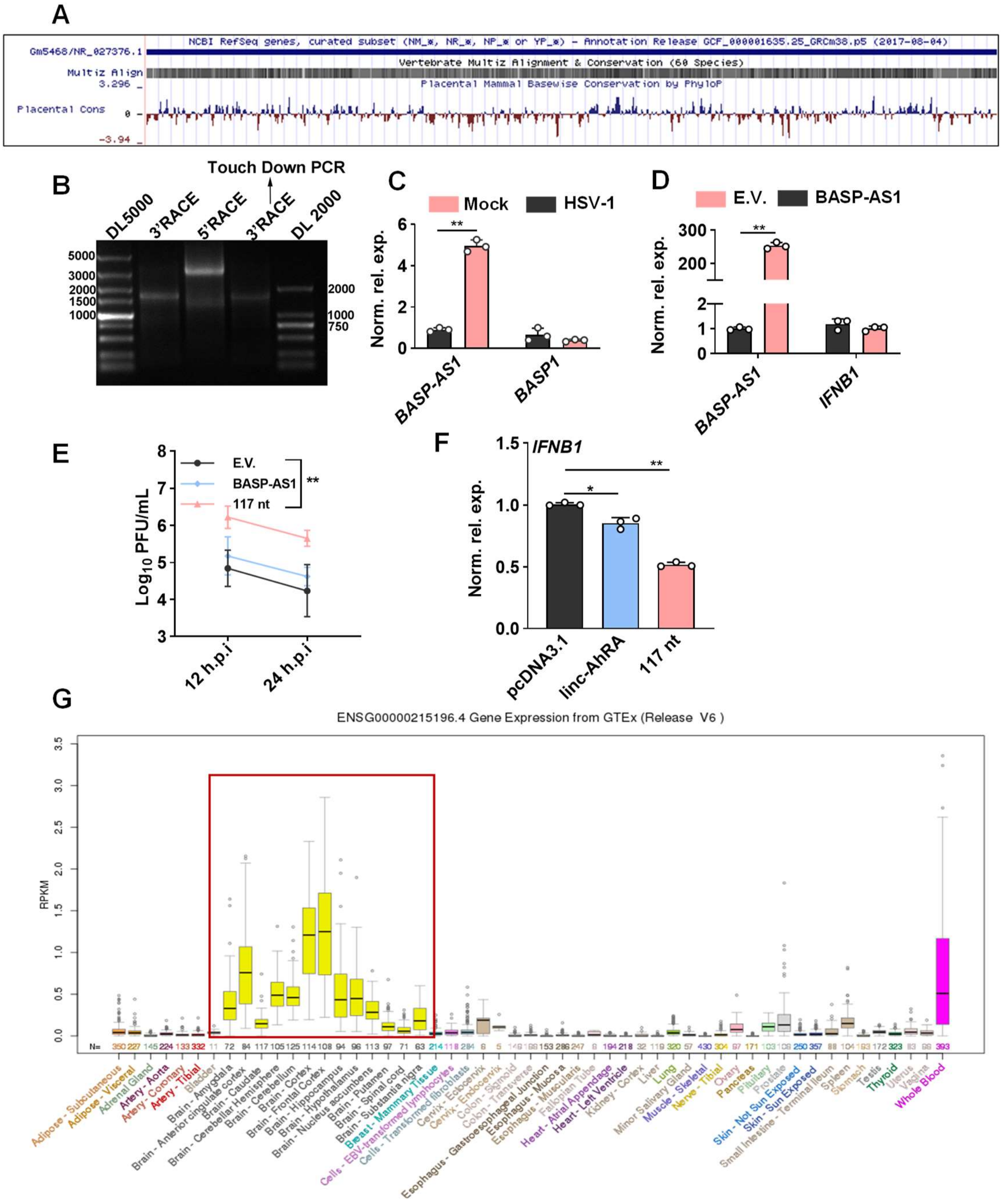


**Figure S12. A,** The vertebrate multiz alignment and conservation (60 species)-based analysis of linc-AhRA conservation and Phylop-based placental mammal basewise conservation. **B,** 5’RACE and 3’RACE results for RNA from HMC3 cells with HSV-1 infection for 12 h to obtain the 5’ and 3’ end sequences for BASP-AS1. **C,** qPCR analysis of *BASP-AS1* and *BASP1* expression in HMC3 cells following a 12-h infection with HSV-1 (MOI 1). **D,** qPCR analysis of *IFNB1* and *BASP-AS1* levels in BASP-AS1-expressing and E.V. plasmids-transfected BV2 cells with HSV-1 infection (MOI 1) for 6 h; **E,** Determination of HSV-1 titers in culture medium supernatants from HMC3 cells transfected with linc-AhRA- or conserved 117nt-expressing plasmids followed 48 h later by HSV-1 infection (MOI 1) for the indicated duration using a plaque formation assay. **F,** qPCR analysis of *IFNB1* levels in HMC3 cells transfected with linc-AhRA or conserved 117nt-expressing plasmids followed 48 h later by HSV-1 infection (MOI 1) for 12 h. **G,** The expression of *BASP-AS1* in human

tissues as presented in GTEx (**version 6.0**). Data are representative of three independent experiments (**B**) or three independent experiments with n = 3 technical replicates **(C-F),** each symbol represents an individual technical replicate (**C-D, F**) (shown as mean and s.d. in **C-F**), two-tailed unpaired Student's t-test (**C-D, F**), two-way ANOVA (**E**).

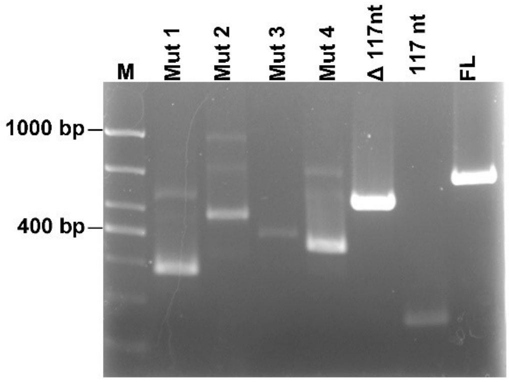


**Figure S13**. PCR analysis of indicated linc-AhRA deletion mutants amplified from corresponding plasmids. Data are representative of three independent experiments.

A

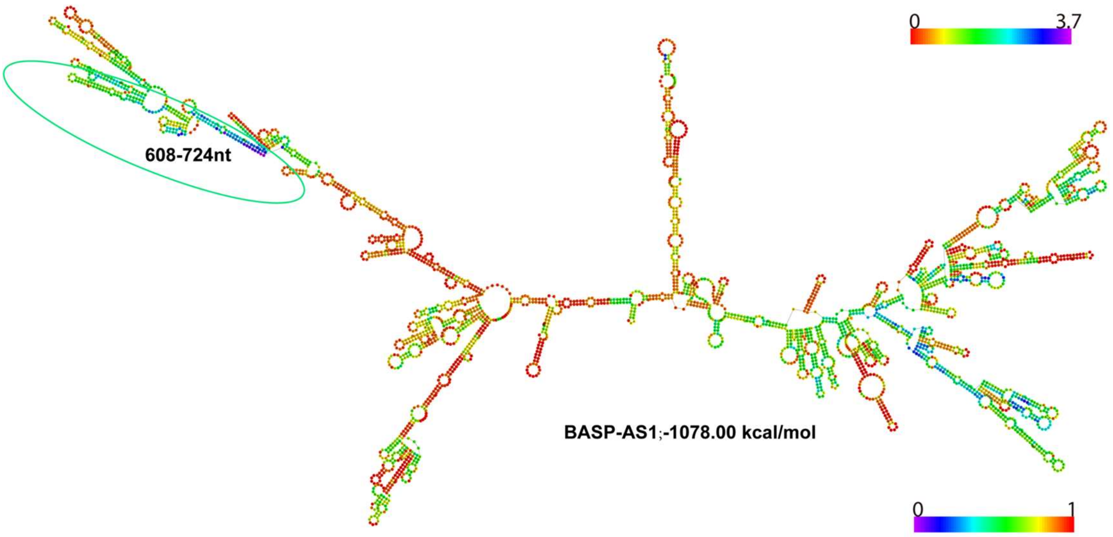


B

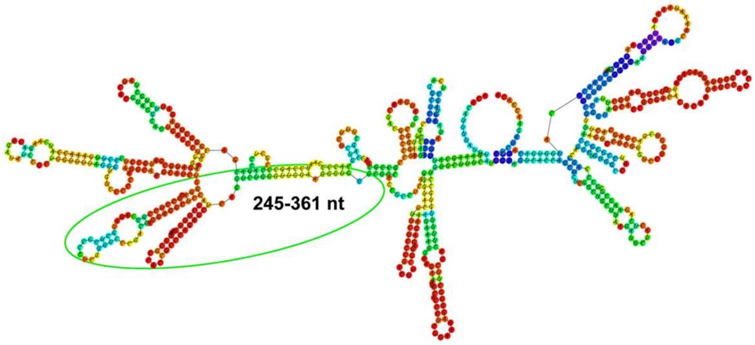


**Figure S14.** Secondary structure of *BASP-AS1 (***A***)* and linc-AhRA (**B**) with minimum free energy predicted by the Vienna RNA web server. The ensemble free energy for them is also labeled.

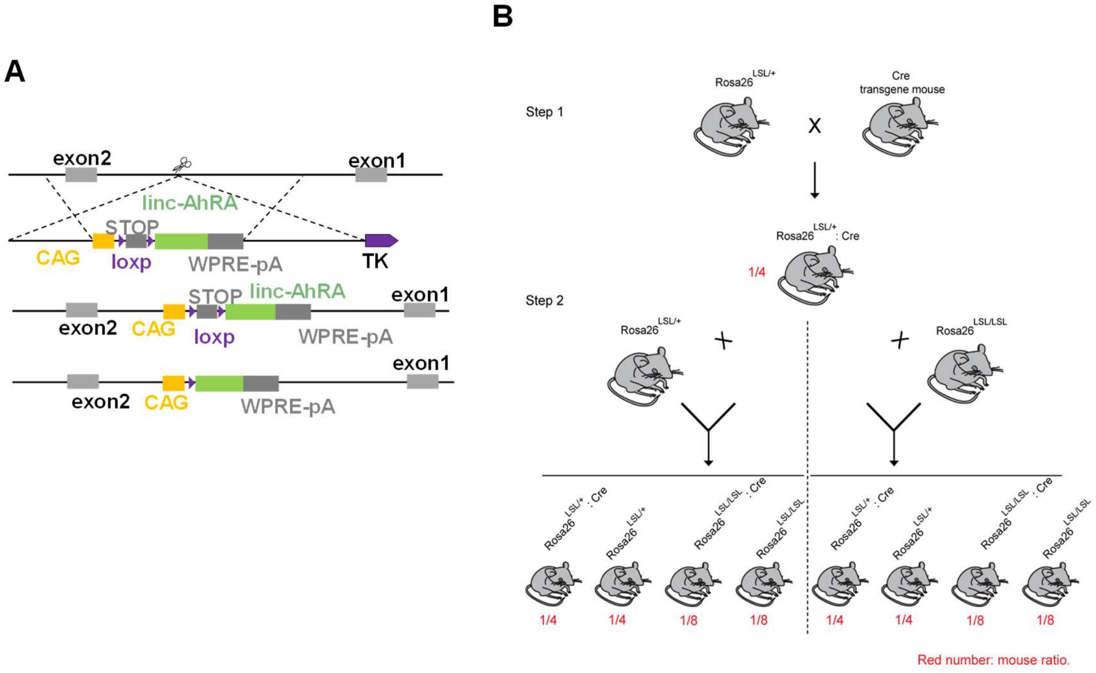


**Figure S15. A,** Strategy for establishing Rosa26-LSL-*linc-AhRA* mice. CAG, CAG promoter; WPRE, woodchuck hepatitis virus post-transcriptional regulatory element; pA, PolyA. **B,** Breeding scheme for generating control and inducible microglial linc-AhRA KI mice.

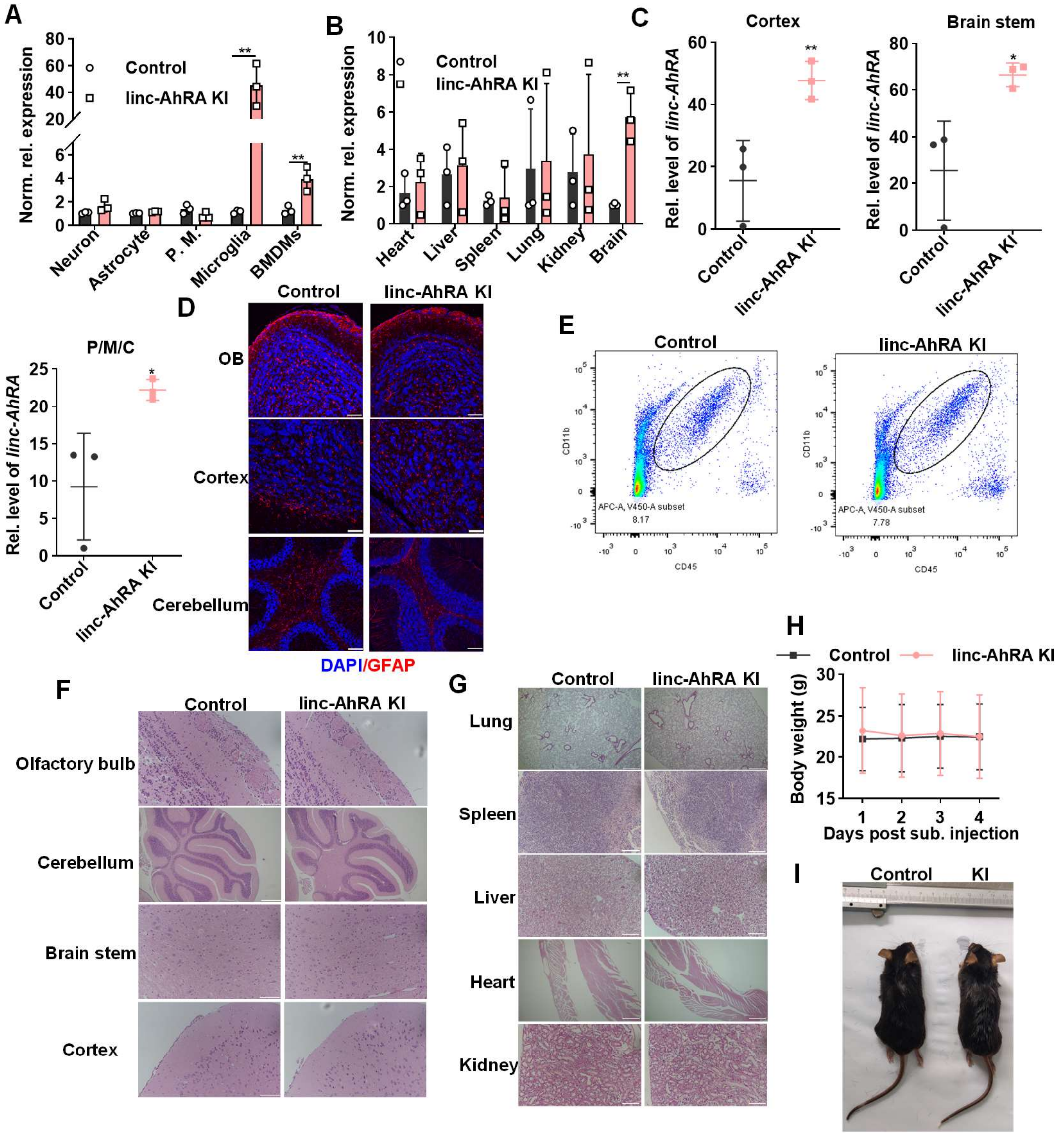


**Figure S16. A,** qPCR analysis of the expression of linc-AhRA in the indicated primary cells isolated from control and microglial linc-AhRA KI mice. All linc-AhRA KI mice described in this figure represented those mice have been received a treatment of TAM. P.M., peritoneal macrophages. BMDMs, bone marrow derived macrophages. n = 3 mice per group. **B,** qPCR analysis of the expression of linc-AhRA in the indicated tissues of control and microglial linc-AhRA KI mice. n = 3 mice per group. **C,** qPCR analysis of the expression of linc-AhRA in the indicated brain sections of control and microglial linc-AhRA KI mice. n = 3 mice per group. **D,** Brain histology of several brain regions that were subjected to immunofluorescence for GFAP to detect astrocytes. Scale bars represent 100 μm. n = 3 mice per group. **E,** Flow cytometry analysis of microglia in single-cell suspension generated with the digestion of brain tissue from control and microglial linc-AhRA KI mice. The ratio of the $CD11b^{+}CD45^{low}$ subset is also labeled. n = 3 mice per group. **F,** Brain histology for several brain regions that were stained with H&E. Scale bars represent 100 μm. n = 3 mice per group. **G,** The H&E stain-based histology of the indicated tissues isolated from WT and microglial linc-AhRA KI mice, n = 3 mice per group. Scale bars represent 500 μm (lung) and 100 μm (spleen, liver, heart, and kidney). **H,** The weight changes in control and microglial linc-AhRA KI mice (n = 4-6 mice per group, 8-9 weeks old) over a period of 4 days. **I,** Photograph of control

and linc-AhRA KI mice aged 8-9 weeks to record body size. Data are representative of three independent experiments (**A-G**) and each symbol represents an individual mouse (**A-C**). Two-tailed unpaired Student's t-test for (**A-C**), two-way ANOVA for **H**.

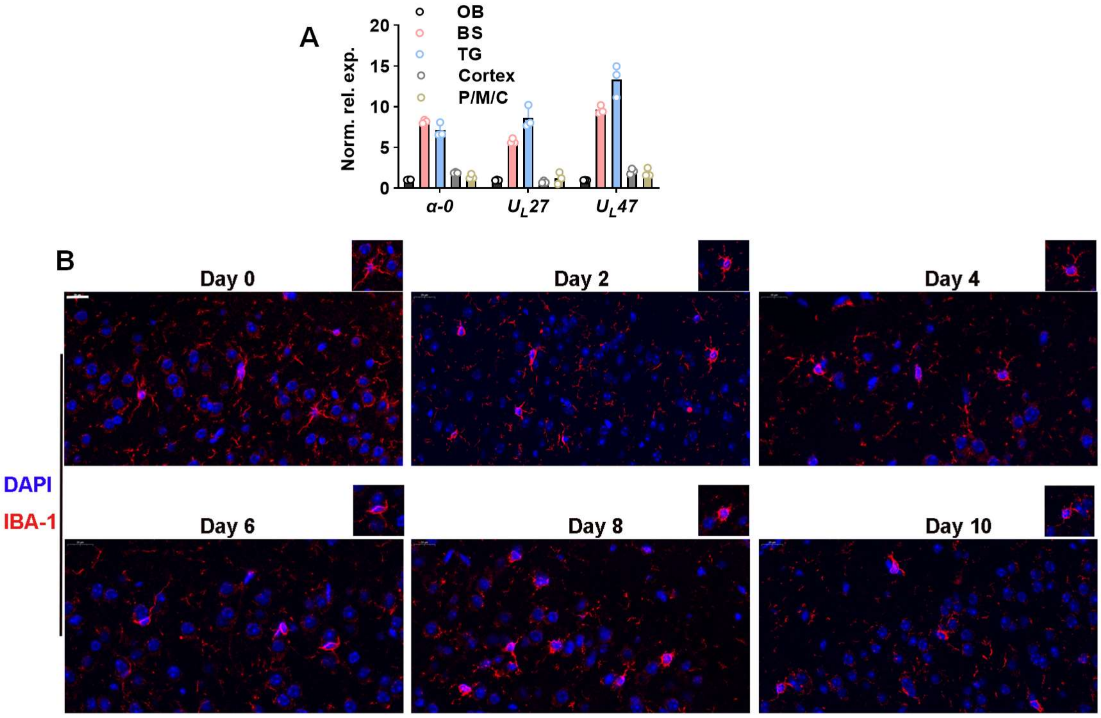


**Figure S17. A,** Determination of viral DNA copies in homogenized tissues of several brain sections from WT mice at 9 days after intranasal inoculation with HSV-1 ($1\times10^7$ PFU/mouse) using qPCR, n = 3 mice, each symbol represents an individual technical replicate. **B,** Tissue sections of BS from mice infected intranasally with HSV-1 ($1\times10^7$ PFU/mouse) for the indicated days were stained with an antibody against IBA-1, n = 3 mice per group. Scale bars represent 20 μm. Data are representative of three independent experiments with n = 3 technical replicates (**A**), three independent experiments (**B**) (shown as mean and s.d. in **A**).

## Supplemental tables

**Table S1. Top15 DELs ranked by basic FPKM and $FPKM_{HSV-1}/FPKM_{Cell}$.**

| Top15 DELs ranked by $FPKM_{HSV-1}/FPKM_{Cells}$ | | Top15 DELs ranked by basic FPKM | |
|---|---|---|---|
| **Transcript ID** | **Log2(fold change)** | **Transcript ID** | **Cell FPKM** |
| LNC_000361 | 8.842804802 | LNC_003485 | 1.549412 |
| LNC_004084 | 8.03634865 | ENSMUST00000150145.1 | 0.896818 |
| LNC_003732 | 7.992301174 | ENSMUST00000231483 | 0.816353 |
| LNC_004073 | 7.426886355 | LNC_005428 | 0.291923 |
| LNC_004433 | 7.330950785 | LNC_003311 | 0.145579 |
| LNC_000350 | 7.272670066 | LNC_002885 | 0.144511 |
| LNC_001407 | 7.245165248 | ENSMUST00000180868.1 | 0.129002 |
| LNC_005318 | 7.206946765 | LNC_001159 | 0.125979 |

| | | | |
|---|---|---|---|
| linc-AhRA | 6.945560733 | LNC_005733 | 0.099695 |
| LNC_000602 | 6.812333042 | LNC_002954 | 0.091674 |
| ENSMUST00000160698.1 | 6.781017341 | LNC_000350 | 0.07221 |
| LNC_002045 | 6.707534069 | linc-AhRA | 0.065611 |
| LNC_003091 | 6.637491442 | LNC_002045 | 0.064985 |
| LNC_002206 | 6.447464883 | LNC_005885 | 0.05853 |
| LNC_002885 | 6.404661921 | LNC_005884 | 0.054507 |

**Table S2. Full-length sequence of linc-AhRA as determined by RACE**

ACACATTTTATTTGAAAGCATTGAATAAGTAGCTCAGGCTCTCGCTCAGACTTAAGG
GGCTGATGAACTACGCCCCCGCCGCAACCAGCTTTCTCTGCACCCCAATCCAGCCCC
GCTCCAGACGACGGGCTTAGATTCTGCTATCACGTAGGCAGTGAGCCTGGGGAAGA
TGCGATGCCTCCCTTGAAACCATCAAGACAGATATCCGCTGGTTTTAGGGTGTCCCC
CAAGGCAAGCATCTGGGACGCCTCCGGCATATGCTCGCCCTCGGGGCCCGACCAGG
AGGCTCCAGCTTTGCAAGGCGCCACCGCCCAGAGCTCAGGTCTGGGTTGGAAGGGT
GGGCGGCACCGCCTCAGGGTACGTTCTCCCGACTTCGGAGAGAAAGAAAGGAAGA
AATGCACCCTAGGAAAAGGGGCTTCGACTTTTTGTGCCTGTGCTTTCATGGAAGTCA
CCAGCTCTCGGCTGCTTAAGATGCTACTCAGAAGCGCGCCCAGCCAGGCTGTGGCC
ATCCGCGCCAAGTCCTCGTGTCCTGGTCGACGAGCACTTTGGGTAGCACCGCGACA
CACAATAGCCACCACGGTTAGGAGATATGCTCGCTATGCTTACCTGTTGGCAAAAATA
AAATGCAAAATAAAAATTTAAATTTGGAAACTAACACATGGTGACATCTTCTAGAAC
CACA

**Table S3. Potential transcription factors (TFs) regulating the expression of linc-AhRA**

| Name | Score | Start | End | Sequence | Mutant |
|---|---|---|---|---|---|
| Ahr::Arnt | 8.06995 | 314 | 319 | agcgtg | atcata |
| Ahr::Arnt | 8.06995 | 1494 | 1499 | agcgtg | atcata |
| Arid3b | 7.60394 | 496 | 506 | cttttaatggt | |
| Ar | 7.14583 | 809 | 825 | ccgaacctcgtgttcct | |
| Arid3a | 6.85157 | 708 | 713 | ataaaa | |
| Arid3a | 6.85157 | 713 | 718 | ataaaa | |
| Arid3b | 6.15987 | 201 | 211 | gttttattcta | |
| Ahr::Arnt | 6.15805 | 451 | 456 | tgtgtg | tatatc |
| Arid3a | 6.02799 | 421 | 426 | atgaaa | |
| Arid3b | 6.01764 | 776 | 786 | gctttaatctg | |

**Table S4. Full-length sequence of BASP-AS1 as determined by RACE**

CTGCTCCCCTCCCCTCCTCCGGCGCAGACCCTCCCCTCTCCCCTCCAGCCTGGACAC
GCCCGCCTCCCCTTGACTCCCCCCAGCTCTGGGCCCCCACCTCCCCTCCCCTCCAGC
ACAGTCACCCCCATTTCTCTCCTATCCGCCATCCTGGTTCCTCCCTTCCCCCCACCTC
CCAACTCTGTGCCCCGCCAACGTTTCCTAAATGCCCTCTATTCAGATCCCCCCTCCGC
CTCCCCTCTCCTCTCCTCCATTCCTGCGTCCCCCTTCCCCCGCCGCGCCGCCTGGGCT
GTCCGTGGACTTCTCCCACTCTCTCACTCTCTCACTCACTCTCTCTCTCTCTCTCT
CTCTCACTCTCTCTCTCTCTCTCCCCCTCATTTATTTGGAACCGTTGGATAAGAAGTG
CTCGGGCTCTCGCTCAGACTTAGGGAGCTGCCTCGAGGTGATGAATGACACCCCCT
GGCACCAGCTACCCTTCTCAGACCCCAGTCCAGCCCGCTCCCGACGTCGACTACGAT
TCCGCTACCTCGGCTGGCAGCGAGGTTGGGGTGAGCCCCAGCTGCAGGCGCGTCTG
GGCTGCGCCGCTGCAAACGAGTTGCGCACCTTGGGCGGCTCCGCACCTGCACCCGC
ACCCGCGGGGCTCAGCCCCGAAGGCTGCAGCTTCGGGGGAGGCGCGGTCGCCGAG
GTCCAGCTGGTGGGGCGAGAGACGTCGCCCCTCGGAGGATGCTCTCGGAACTTGGG
AGAGGAAGGAGGGAAGAGAAGAGGGGAAAGGGGCCGTCGATGTTTTTGATGTCTG
TGCTTTAATGGAGGCCACCAATATTGAGAAGACGGGGTTGGCCGAGGCAGCCCGCA
CGCTGCTGCTTGCGAGCGCTCGAGTCAAAGCTAGGGCCAACCGCGGCTTGTCCGGG
TGCCCTAAGGGGGCGGACACTTGGTTTAGCACCGGGACACAGAATAGCCACCGGGG
TAGGAAGATGCGTTCACTTTGCTTACCTGTTGGCAAGAGGGACATACAAAAATAACG
TAACGTGACATCGTTGACAACGGTAGCTCTTTGATTACACAAAAGCCAATTTTACCTT
CCCGCAAAAGCCAGTTGACGCCTTTGGAACTTTTATTTGCGGCATTTTGGCGCCCTC
TGGCTGTGTTTGGATCGCTTTCATGCTCGCCTGCGTCCCAGCCAAGAAAAAATCGAT
GGAGCTGCAGGTTGTCCTCAGGATGGTTCTGCCCTCAGGACCTGGGCGTGAATTCA
GGGACAGGGTGGCCCTCCAGAACCGGAGTGACAAACTGTAACCATACTTAGGGAGG
CAGACGTCAAAGGCAAGTACATCTGTATTCAACTGGGTAAAGCCATGTGAAGACGT
GCCTGCCTCCCTGTTACCTTCCACCATGATTGTAAGCTTCCTGAGGCCTCCCCAGCCA
TGCCTGCTGTATAGCCTGCAGAACAGTTTCAGTCGATTTGATTATAAAGCAAAGGTTT
GGGAAGAAAGACTCATATTCCTCCTGAGATGCAACCTCAAGTCTTAGGAAAAGAAA
GTTTTGAACTCAGGTTCCGGGAATGTGGAAGAGGAAGCTCTTAAAGGGCAAGTAGA
CTTTGCAACATGCTCGTTTTCAGGATTCTCCTCCTTTGTCCTCAACCCTGCCCAGTGT

TCCCCTAACTCCACCTAAGCCACTCCATAAAGTTAGGTCCCATTCCTCTATTCCTTCA
CTACTAATGACAGCTAATAGAGCTAAACAGAATACCACAATAAGCCAGAAGCTGCCT
TTTTTTATTATTATTATTATTCATTGCCCAATTTGTAGAACTCTCTGAATTCAATTAAA
GTTTGGCAAAGCCTGTGCAAATGAAACCAAGTACCTATTTTTTGCTCATCATAAACC
CAAAAGTTTTCTAGAGAACTAACTGAAAGAGATTTTCACCAAATCTTTTTATTTTTTT
AATCTAGAGAATACAATTGAGAACCAAATCAATAAATATCCTCAACTGTTACCTTTGT
TATAAGGGAACTACGATGAACCGTGCTTGCCCCACATTTACCCTAGCAGCAACTATG
CTTTTTCTATCTCTGGCCTTACCCTGCCTTCCTGCCTCCAGAGTCTGAGATGGAGAAA
GGCAAAGTCAGATGGAGGATAGAGCTGGGCAGGGAGTTGCTGCCAGCAACAATTG
GAGTTGCTGGTTTGCTTTCAACACTGACCCCACTTTATTGGCCATGAGTTAAGGCAG
TCAAATGGGCATCTAGGAAACATGACCAAGATCTGCATTAGGGAAGCAAAGCAGAT
TAAAAGGCACAATTGCTGGCCAGGCATGGTGGCTCACACCTCTAATCCTAGTGAGAG
GTGAAGCCAGTTGGACTTCCTGGGTCGAGTGGGGTGGGGTCTTGGAGAAATTTTCT
ATCTAGCTAGAGGATTATAAATGCACCAATCAGCTCTGTGTCTAGCTAAAGGTTTGTA
AACGCACCAATCAGCACTCTGTAAAAACGCACCAATCAATGCTCTGTGTCTAGCTGA
AGGTTTGTAAATGCACCAATCAGCACTCCGTAAAACGGACTGATCAGTGCTTTGTAA
AATGGACCAATCAGCAGGATGTAGGCAGGGCCAAATAAGGGAAAAAAAGCTGGCA
CCCAAGCCAACAGGGGCAACCTGCTTGAGTCCCTTTCCACATTGTGGAAGCTTTGTT
CCTTCACTCTTCATGATAAATCTTGCTGCTGCTCACTCTTTGGGTCCGCACTACCTTT
ATGAGCTGTAACACTCACCACGAGGGTCTGTGGCTTCATTCCTTAAGTTAGCAAGAC
CACGAACCCACAGGGAGGAACAAACAACTCCGGGCACACCACCTTTAAGAGCTATA
ACACTCACTGCGAAGGTCTGTGGCTTCACTCCTGTAGTCAGCAAAACCACAAACCC
ACCGGAAGGAAGAAACTCCAGACACATCTAAACATCTGAAGGAACAAACTCTGGA
CACACCATCTTTAAGAACTGTAACACTCACCGTGAGGGTCCGTGGCTTCATTCTTGA
AGTCAGGGAGACCAAGAACCCACCGGAAGGAACAAACTCCGGACACACTAGCACT
TACAGGAGGCCCAGGTGGGAAGATCACTTGAGGCCAGGAGTTTGAGACCAGCCTG
GGCAACGTAGTCAGACCCCATCTCTACAAAAAAATTTAAAAAATTAGCCGAGCATGG
TGGCAAGTGCCAGTAATCCCAGCTACTCTTGGAGGCTGAGGCAGGAAGAGCCCTTG
AGCCCAGGAGCTGGAGGCTGCAGTGAACTATGATCAAACCACTGTAGTCCAGCCT
GAGTGACAGAGTGAGACCCTGCCTCTAAAAAAAAAAAAAA

**Table S5. The interaction propensity between linc-AhRA and TBK1**

| # | Protein region | RNA region | Interaction Propensity | Discriminative Power | Normalized Score |
|---|---|---|---|---|---|
| 1 | 54-105 | 301-352 | 13.92 | 37 | 2.92 |
| 2 | 476-527 | 301-352 | 13.81 | 37 | 2.89 |
| 3 | 651-702 | 301-352 | 13.22 | 37 | 2.76 |
| 4 | 54-105 | 307-358 | 12.55 | 35 | 2.6 |
| 5 | 476-527 | 307-358 | 12.43 | 35 | 2.58 |
| 6 | 654-705 | 301-352 | 12.36 | 35 | 2.56 |
| 7 | 604-655 | 301-352 | 12.04 | 35 | 2.49 |
| 8 | 154-205 | 301-352 | 11.93 | 33 | 2.46 |
| 9 | 651-702 | 307-358 | 11.67 | 33 | 2.41 |

| | | | | | |
|---|---|---|---|---|---|
| 10 | 326-377 | 301-352 | 11.25 | 33 | 2.31 |
| 11 | 301-352 | 301-352 | 11.03 | 33 | 2.26 |
| 12 | 401-452 | 301-352 | 11.02 | 33 | 2.26 |
| 13 | 654-705 | 307-358 | 10.81 | 32 | 2.21 |
| 14 | 651-702 | 326-377 | 10.81 | 32 | 2.21 |
| 15 | 554-605 | 301-352 | 10.68 | 32 | 2.18 |
| 16 | 604-655 | 307-358 | 10.65 | 32 | 2.17 |
| 17 | 154-205 | 307-358 | 10.63 | 32 | 2.17 |
| 18 | 576-627 | 301-352 | 10.59 | 32 | 2.16 |
| 19 | 104-155 | 301-352 | 10.56 | 32 | 2.15 |
| 20 | 51-102 | 301-352 | 10.45 | 32 | 2.13 |

**Table S6. The interaction propensity between linc-AhRA and TRIM27**

| # | Protein region | RNA region | Interaction Propensity | Discriminative Power | Normalized Score |
|---|---|---|---|---|---|
| 1 | 188-239 | 301-352 | 19.28 | 52 | 4.03 |
| 2 | 188-239 | 307-358 | 17.46 | 47 | 3.63 |
| 3 | 188-239 | 326-377 | 15.52 | 42 | 3.21 |
| 4 | 438-489 | 301-352 | 14.3 | 40 | 2.94 |
| 5 | 188-239 | 226-277 | 13.9 | 37 | 2.85 |
| 6 | 126-177 | 301-352 | 13.55 | 37 | 2.77 |
| 7 | 188-239 | 282-333 | 13.39 | 37 | 2.74 |
| 8 | 438-489 | 326-377 | 13.14 | 37 | 2.68 |
| 9 | 188-239 | 232-283 | 13.01 | 37 | 2.65 |
| 10 | 438-489 | 307-358 | 12.94 | 35 | 2.64 |
| 11 | 88-139 | 301-352 | 12.92 | 35 | 2.63 |
| 12 | 188-239 | 251-302 | 12.75 | 35 | 2.59 |
| 13 | 288-339 | 301-352 | 12.66 | 35 | 2.58 |
| 14 | 188-239 | 207-258 | 12.64 | 35 | 2.57 |
| 15 | 326-377 | 301-352 | 12.58 | 35 | 2.56 |
| 16 | 226-277 | 301-352 | 12.18 | 35 | 2.47 |
| 17 | 126-177 | 307-358 | 12.1 | 35 | 2.45 |
| 18 | 88-139 | 307-358 | 11.57 | 33 | 2.33 |
| 19 | 288-339 | 307-358 | 11.37 | 33 | 2.29 |
| 20 | 326-377 | 307-358 | 11.33 | 33 | 2.28 |

## Table S7

| REAGENT or RESOURCE | SOURCE | IDENTIFIER |
|---|---|---|
| **Cell lines (All cell lines were negative for mycoplasma contamination)** | | |
| BV2 | National Infrastructure of Cell Line Resource | 3111C0001CCC000063 |

| | | |
|---|---|---|
| HMC3 | American Type Culture Collection | CRL-3304 |
| L929 | American Type Culture Collection | N/A |
| HEK 293T | American Type Culture Collection | N/A |
| Neuro-2a | American Type Culture Collection | CCL-131 |
| Vero | American Type Culture Collection | CCL81 |
| **Virus Strains** | | |
| HSV-1 F strain | Prior study (1) | N/A |
| HSV-1 EGFP reporter strain | Prior study (1) | N/A |
| Influenza A virus PR8(strain A/Puerto Rico/8/1934 H1N1) | Prior study (2) | N/A |
| CVB3 | Prior study (3) | N/A |
| **Antibodies** | | |
| cGAS (For Human) | Cell Signaling Technology | Cat#15102S |
| cGAS (Mouse specific) | Cell Signaling Technology | Cat#31659S |
| STING | Cell Signaling Technology | Cat#13647S |
| TBK1 | Cell Signaling Technology | Cat# 3504S |
| Phospho-TBK1 (Ser172) | Cell Signaling Technology | Cat# 5483S |
| IRF3 | Cell Signaling Technology | Cat#4302S |
| Phospho-IRF3(Ser396) | Cell Signaling Technology | Cat#4947S |
| MAVS | Santa Cruz | Cat#SC-166583 |
| RIG-1 | Cell Signaling Technology | Cat#4200S |
| DDX19A | Sigma-Aldrich | Cat#HPA045252 |
| DTX4 | Proteintech | Cat#25222-1-AP |
| TRAIP | Proteintech | Cat#10332-1-AP |
| STAT1 | Santa Cruz | Cat#sc-464 |
| Phospho-STAT1 | Santa Cruz | Cat#sc-8394 |
| Phospho-JAK1 | Cell Signaling Technology | Cat#3331S |
| GAPDH | Cell Signaling Technology | Cat #2118S |
| AhR | GeneTex | Cat#GTX22770 |
| TRIM27 | Proteintech | Cat#12205-1-AP |
| Ubiquitin | Cell Signaling Technology | Cat#3936 |
| NeuN | Proteintech | Cat#26975-1-AP |
| GFAP | Proteintech | Cat#16825-1-AP |
| FLAG | Beyotime | Cat # AF5051 |
| HA | Cell Signaling Technology | Cat #3724 |
| IBA1 | Proteintech | Cat #10904-1-AP |
| Alexa Fluor Secondary Antibodies | Thermo Scientific | Cat #A11001; A21206; A21203; A21442; Z25308 |
| APC anti-mouse CD11b(M1/70) | BioLegend | Cat #101211 |
| APC/Cyanine7 anti-mouse CD45 | BioLegend | Cat #103116 |
| Mouse IgG | Cell Signaling Technology | Cat #3420S |

| | | |
|---|---|---|
| (Sepharose® Bead Conjugate) | | |
| Rabbit IgG (Sepharose® Bead Conjugate) | Cell Signaling Technology | Cat #3423S |
| Mouse HRP-conjugated | Cell Signaling Technology | Cat #7076S |
| Rabbit HRP-conjugated | Cell Signaling Technology | Cat #7074S |
| **Chemicals and Recombinant proteins** | | |
| 5'ppp-dsRNA | InvivoGen | Cat #tlrl-3prna |
| 3'3'-cGAMP | InvivoGen | Cat #tlrl-nacga |
| 2'3'-c-di-AM(PS)2 (Rp,Rp) | InvivoGen | Cat #tlrl-nacda2r-01 |
| Indirubin | TargetMol | Cat #T6169 |
| Darolutamide (ODM-201) | TargetMol | Cat #T6915 |
| CH 223191 | TargetMol | Cat #T2448 |
| MG132 | TargetMol | Cat #T2154 |
| Z-VAD-FMK | Beyotime | Cat #C1202 |
| Cycloheximide | 4A Biotech | Cat #FXP130 |
| L-Kynurenine | Selleck | Cat #s5839 |
| β-mercaptoethanol | Macklin | Cat #M6230 |
| Tert-Pentyl Alcohol | Macklin | Cat #A800282 |
| Mouse IFNβ | R&D Systems | Cat #8234-MB-010 |
| 3-MA | Sigma-Aldrich | Cat #M9281 |
| CT-DNA | Solarbio | Cat #D8020 |
| Poly(I:C) | Sigma-Aldrich | Cat #P1530- |
| Restricted enzymes | Takara | N/A |
| Tribromoethanol | Meryer Chemical Technology Company | Cat #M33110 |
| Tamoxifen | MedChemExpress | Cat #HY-13757A |
| Corn oil | YuanyeBio | Cat #S50856 |
| DMSO | Sigma-Aldrich | Cat #D2650 |
| **Main kits** | | |
| EndoFree Maxi Plasmid Kit | TIANGEN | Cat #DP120 |
| Mouse IFN-β ELISA Kit | 4A Biotech | Cat #CME0116 |
| RNAprep Pure Micro Kit | TIANGEN | Cat #DP420 |
| RIP Kit | Millipore | Cat #17-701 |
| RNA pull-down Kit | Pierce | Cat #20164 |
| Silver stain Kit | Beyotime | Cat #P0017S |
| SMARTer RACE 5’/3’kit | Takara | Cat #634858 |
| CUT & Tag kit | Novoprotein | Cat #N259-YH0 |
| EasyPure® Viral DNA/RNA Kit | TransGen Biotech | Cat #ER201 |
| BCA Protein Assay Ki | Beyotime | Cat #P0011 |
| Mouse T ail Direct PCR Kit-UNG | FORGENE | Cat #TP-01341 |

| | | |
|---|---|---|
| **Software** | | |
| Prism version 8.0 | GraphPad Software | N/A |
| Coding Potential Calculator | Peking University | http://cpc.cbi.pku.edu.cn/programs/run_cpc.jsp |
| Multiple Alignments of 60 vertebrates | *UCSC* Genome Browser | http://genome.ucsc.edu/ |
| Adobe illustrator CC 2015 | Adobe Systems Incorporated | N/A |
| FlowJo™ | Becton, Dickinson and Company | N/A |
| Image J | National Institutes of Health | https://imagej.nih.gov/ij/ |
| catRAPID | RNA Systems Biology - Italian Institute of Technology (IIT) | http://service.tartaglialab.com/page/catrapid_group |
| UbiBrowser | Beijing Institute of Radiation Medicine | http://ubibrowser.ncpsb.org/ubibrowser/ |
| RNAfold web server | University of Basel | http://rna.tbi.univie.ac.at/cgi-bin/RNAWebSuite/RNAfold.cgi |
| Illustrator for Biological Sequences | Sun Yat-sen University | http://ibs.biocuckoo.org/ |
| ZEN 2 (blue editon) for Microscope | ZEISS | N/A |
| NIS viewer (version 4.5) for Microscope | Nikon | N/A |

**Table S8. Primers for qPCR analysis in this study**

| Genes | Forward (5'-3') | Reverse (5'-3') |
|---|---|---|
| m*Ifnb1* | ATGAGTGGTGGTTGCAGGC | TGACCTTTCAAATGCAGTAGATTCA |
| m*Trim27* | CTCACGCTACCTTCTGTGGG | CCCAACATGGCCAGAAAACC |
| m*Cxcl10* | CCAAGTGCTGCCGTCATTTTC | GGCTCGCAGGGATGATTTCAA |
| m*Ifnα4* | TGATGAGCTACTACTGGTCAGC | GATCTCTTAGCACAAGGATGGC |
| m*Isg15* | GATTGCCCAGAAGATTGGTG | TCTGCGTCAGAAAGACCTCA |
| *linc-AhRA* | TATCACGTAGGCAGTGAGCC | GGGGGACACCCTAAAACCAG |
| m*Il-6* | CACAGAGGATACCACTCCCAACA | TCCACGATTTCCCAGAGAACA |
| m*Tnf* | CATCTTCTCAAAATTCGAGTGACAA | CCAGCTGCTCCTCCACTTG |
| m*Mx2* | GAGGCTCTTCAGAATGAGCAAA | CTCTGCGGTCAGTCTCTCT |
| m*Gapdh* | TGTGTCCGTCGTGGATCTGA | CCTGCTTCACCACCTTCTTGA |
| m*Cgas* | CTGCGCAGAATGCAGAAACG | CTTGTAGCTCAATCCTGGGGA |
| m*Sting* | GCTGCTGATGCCATACTCCA | TGGATCCTTTGCCACCCAAA |
| m*Tbk1* | ATCAAGAAGGCACGCATCCA | GGCTCATTGCTTTTGTGGCA |
| *mIrf3* | AGGCTTGTGATGGTCAAGGT | AATAACCACCAGCCTAGACGC |
| *mU6* | GTGCTCGCTTCGGCAGCACATAT | AAAATATGGAACGCTTCACGAA |

| | | |
|---|---|---|
| *m18S* | CAGCCACCCGAGATTGAGCA | TAGTAGCGACGGGCGGTGT |
| *mNeat1* | TTGGGACAGTGGACGTGTGG | TCAAGTGCCAGCAGACAGCA |
| *mBasp1* | GAGAGAGAGAGCCTTTGCTGAG | CTTGCCTCCCATCTTGGAGTT |
| *mCyp1b1* | ACATCCCCAAGAATACGGTC | TAGACAGTTCCTCACCGATG |
| *mTiparp* | GCCAGACTGTGTAGTACAGCC | GGGTTCCAGTTCCCAATCTTTT |
| *hGAPDH* | CACCATCTTCCAGGAGCGAG | AGAGGGGGCAGAGATGATGA |
| *hBasp1* | TTCAGACTCAAAACCCGGCA | CCTTGGGTGTGGAACTAGG |
| *hBasp-as1* | CGAGTCAAAGCTAGGGCCAA | GAACGCATCTTCCTACCCCG |
| *hIfnb1* | TCTCCTGTTGTGCTTCTCCAC | GCCTCCCATTCAATTGCCAC |
| *$U_L47$* | ACGATGATGATGAGGTTCCC | CAGCTCCTCTAGGAACAGCG |
| *α-0* | CCCACTATCAGGTACACCAGCTT | CTGCGCTGCGACACCTT |
| HSV-1-120 | AGACGGTATATTTTTGCGTTATCA CTGTCCCCG | AAGTCCTCCAAAAAACCCGCCACAA ATAAAAAGG |

**Note:** m-prefix means mouse, while h-prefix means human.

**Table S9. Primers for constructing mammalian expression plasmids**

| Genes | Accession | Forward (5'-3') | Reverse (5'-3') |
|---|---|---|---|
| lincAhRA (antisense) | N/A | cttggtaccgagctcggatccTGTGG TTCTAGAAGATGTCACCAT G | tgctggatatctgcagaattcACACATTTTATTT GAAAGCATTGAATAA |
| m-cGAS(FL) | NM_17338 6.5 | ggccatggaggcccgaattcGGATG GAAGATCCGCGTAGAAGG | gcggccgcggtacctcgagaTCAAAGCTTGTC AAAAATTGGAAA |
| m-cGAS (C teriminal) | XM_00651 1006.3 | ggccatggaggcccgaattcGGATG GAAGATCCGCGTAGAAGG | gcggccgcggtacctcgagaTCACAAGATAG AAAGCACCTGTTTC |
| m-STING | XM_01731 7994.1 | ggccatggaggcccgaattcGGATG CCATACTCCAACCTGCATC | gcggccgcggtacctcgagaTCAGATGAGGTC AGTGCGGAG |
| m-TBK1 | NM_01978 6.4 | ggccatggaggcccgAATGCAGA GCACCTCCAACCATC | cgcggtacctcgagaCTAAAGACAGTCCAC ATTGCGA |
| m-IRF3 | NM_01684 9.4 | ggccatggaggcccgAATGGAAA CCCCGAAACCGCGGA | cgcggtacctcgagaTCAGATATTTCCAGTG GCCTGG |
| m-IRF3 (D) | N/A | aaaaccgtggacttgcacatcgacAAC AGCCAGCCTATCTCCCTT | gtgcaagtccacggttttcaggtcAGAGGCTCCC CCTTCCCG |
| m-IκKα | NM_00770 0.2 | ggccatggaggcccgAATGGAGC GGCCCCCGGGGCTGC | cgcggtacctcgagaTCATTCTGCTAACCA ACTCCAA |
| m-AhR | NM_00131 4027.1 | ggccatggaggcccgaattcGGATG CCATACTCCAACCTGCATC | gcggccgcggtacctcgagaTCAGATGAGGTC AGTGCGGAG |
| mTRIM27 | NM_00905 | ttgcggccgcgaattcatcgATGGCC | ctctagagtcgactggtaccTCACGGAGAGGT |

| | | | |
|---|---|---|---|
| | 4.3 | TCCGGGAGCGTG | CTCCATGG |

**Note:** linc-AhRA(antisense) was cloned into pcDNA3.1(+) plasmids, TRIM27 was cloned into p3×FLAG-CMV-10 plasmids. Other plasmids were cloned into pCMV-HA plasmids.

**Table S10. The sequence of siRNA used in this study**

| Genes | Sense(5'-3') | Antisense(5'-3') |
|---|---|---|
| Trim27-1 | CCACCUAAGAAGAGUGAAAGA | UCUUUCACUCUUCUUAGGUGG |
| Trim27-2 | GCUCAGUUAUACUCAGUUGAU | AUCAACUGAGUAUAACUGAGC |
| Arid3a-1 | CGGGAGCAACAGUAUUAGCAU | AUGCUAAUACUGUUGCUCCCG |
| Arid3a-2 | UGAGGGAGAUAGGCAUUUGAU | AUCAAAUGCCUAUCUCCCUCA |
| Arid3b-1 | CCACAGGGACAACAAACUAAA | UUUAGUUUGUUGUCCCUGUGG |
| Arid3b-2 | GCAUCAAUAUGUCUGUGGAUA | UAUCCACAGACAUAUUGAUGC |
| Ahr-1 | UUUAUGCAUCGGCAACAAUUG | CAAUUGUUGCCGAUGCAUAAA |
| Ahr-2 | GAGAUGCACAAGUACAGUUAU | AUAACUGUACUUGUGCAUCUC |
| Ar-1 | UGGAUGGGACUGAUGGUAUUU | AAAUACCAUCAGUCCCAUCCA |
| Ar-2 | ACGAUUGUACCAUUGAUAAAU | AUUUAUCAAUGGUACAAUCGU |
| N.C. | UUCUCCGAACGUGUCACGUTT | ACGUGACACGUUCGGAGAATT |

**Table S11. The primers for constructing plasmids used in dual luciferase assay**

| Plasmids backbone | Name | Forward (5'-3') | Reverse (5'-3') |
|---|---|---|---|
| pGL4.11-(luc2p) | *linc-AhRA prmoter*-luc. | ggatcttccagagatctcgagATGATG<br>GGGAGATCAGATGGG | gccgttcgacgatagatctGCGAGACAG<br>AGAGAGGAGAGAAAT |
| pGL4.11-(luc2p) | m*Ifnb1 promoter*-luc. | CTGTCTCGAG<br>TTTCTCTTATAGTACACT | CTGTAGATCT<br>GAGCTGCTTATAGTTGAT |
| pGL4.11-(luc2p) | *linc-AhRA promoter*-luciferase (AhR BSs mutant) | BS1-F: GCTCCTTTGAACatcataAAAATCACTGGGGCAGAGGC<br>BS1-R: tatgatGTTCAAAGGAGCACAGCGCA<br>BS2-F: CGCATtatatcCCATCCGATCCAGGGATTCT<br>BS2-R: CGGATGGgatataATGCGTGGCTGGGGACCG<br>BS3-F: CGG*atcata*GAGCGAGTTGGAGCGCTGC<br>BS3-R: AACTCGCTCtatgatCCGGGCTCCGCCGTCGAG | |
| Beyotime | ISRE-luc | Cat# D2179 | |

**Table S12. Primers for RACE assay**

| Gene | 5'RACE | 3'RACE |
|---|---|---|
| linc-AhRA | GATTACGCCAAGCTTCCTTGGG<br>GGACACCCTAAAACCAGCGGA<br>TAT | GATTACGCCAAGCTTCCGCCTCAGGGTA<br>CGTTCTCCCGACTT |

| | | |
|---|---|---|
| BASP-AS1 | GATTACGCCAAGCTTgggtgcgggt gcaggtgcggagc | GATTACGCCAAGCTTgcggctccgcacctgcaccc gcaccc |

**Table S13. Primers for constructing plasmids for tRSA RNA pull-down assay**

| Name | Forward (5'-3') | Reverse (5'-3') |
|---|---|---|
| tRSA-linc-AhRA | aaaaaaaagaattcggatccACACATTTTA TTTGAAAGCATTGAATAA | gctggatatctgcagaattcTGTGGTTCTAGAAGAT GTCACCATG |
| Mut 1 | ttggtaccgagctcggatccACACATTTTAT TTGAAAGCATTGAATAA | gctggatatctgcagaattcCCCAGATGCTTGCCTT GGG |
| Mut 2 | ttggtaccgagctcggatccACGCCTCCGG CATATGCT | gctggatatctgcagaattcTGTGGTTCTAGAAGAT GTCACCATG |
| Mut 3 | ttggtaccgagctcggatccACACATTTTAT TTGAAAGCATTGAATAA | gctggatatctgcagaattcGTACCCTGAGGCGGTG CC |
| Mut 4 | ttggtaccgagctcggatccGTTCTCCCGA CTTCGGAGAGA | gctggatatctgcagaattcTGTGGTTCTAGAAGAT GTCACCATG |
| Δ117nt | ccccaaggcaagcatctgggGTTCTCCCG ACTTCGGAGAGA | gctggatatctgcagaattcTGTGGTTCTAGAAGAT GTCACCATG |
| 117nt | ccaagctggctagttaagcttACGCCTCCG GCATATGCT | tgctggatatctgcagaattcGTACCCTGAGGCGGT GCC |
| pcDNA3.1 (+)-tRSA | Synthesized from Tsingke (Beijing, China) | |

**Table S14.Primers for CUT-&Tag qPCR analysis**

| Genes | Forward (5'-3') | Reverse (5'-3') |
|---|---|---|
| CUT&Tag-set 1 | CAAAATCCTGAAATCGCACCCAT GT | GTTTCCGCCTTGGAGCGAACTCGGT |
| CUT&Tag-set 2 | AAGCGTTCGATGAATATTCATGAA AAGAATGCGGT | AACCAGAATCCCTGGATCGGATGGC |

**Table S15. Targeted sites of LNA against linc-AhRA**

| # | Cat.no. | Targeted sites |
|---|---|---|
| LNA-N.C. | 339515LG00000001-DDA | G*C*T*C*C*C*T*T*C*A*A*T*C*C*A*A |
| LNA-466 | 339511LG00240466-DDA | A*G*A*A*A*G*C*T*G*G*T*T*G*C*G*G |
| LNA-467 | 339511LG00240467-DDA | A*T*C*A*G*C*C*C*C*T*T*A*A*G*T*C |

| | | |
|---|---|---|
| LNA-469 | 339511LG00240469-DDA | A*A*G*C*C*C*G*T*C*G*T*C*T*G*G*A |

**Table S16. Primers for linc-AhRA genotyping of mice**

| Primer | Sequence (5'→3') | Primer type |
|---|---|---|
| P1 | TCAGATTCTTTTATAGGGGACACA | Forward |
| P2 | TAAAGGCCACTCAATGCTCACTAA | Reverse |
| P3 | CTCGGCTGCTTAAGATGCTACTC | Forward |
| P4 | GACGATGATTTCCCCGACAAC | Reverse |

**Table S17. Primers for Cre genotyping of mice**

| Primer | Sequence (5'→3') | Primer type |
|---|---|---|
| P1 | AAGACTCACGTGGACCTGCT | Common Forward |
| P2 | CGGTTATTCAACTTGCACCA | Mutant Reverse |
| P3 | AGG ATGTTGACTTCCGAGTTG | Wild type Reverse |

## Reference


1. **Wang Y, Huang L, Wang Y, Luo W, Li F, Xiao J, Qin S, Wang Z, Song X, Wang Y, Jin F, Wang Y.** 2020. Single-cell RNA-sequencing analysis identifies host long noncoding RNA MAMDC2-AS1 as a co-factor for HSV-1 nuclear transport. Int J Biol Sci **16:**1586-1603.
2. **Ge H, Liu G, Xiang YF, Wang Y, Guo CW, Chen NH, Zhang YJ, Wang YF, Kitazato K, Xu J.** 2014. The mechanism of poly-galloyl-glucoses preventing Influenza A virus entry into host cells. PLoS One **9:**e94392.
3. **Wang X, Wang Y, Ren Z, Qian C, Li Y, Wang Q, Zhang Y, Zheng L, Jiang J, Yang C, Wang D, Zhang Y, Fan J, Wang Y.** 2012. Protective effects of 20(s)-protopanaxtriol on viral myocarditis infected by coxsackievirus B3. Pathobiology **79:**285-289.
